# Systems pharmacology identifies ajugol-mediated NF-κB/caspase-3 inhibition and isoacteoside-driven p62/mTOR-mediated autophagy as key mechanisms of Rehmanniae Radix and its processed form in Alzheimer’s treatment

**DOI:** 10.3389/fphar.2025.1644847

**Published:** 2025-08-29

**Authors:** Xiang Han, Xianglong Meng, Yuhui Wu, Wei Xia, Simin Xue, Xiaoqin Liu, Chenzi Lyu, Ziang Li, Xiaoning Yan, Hyo Won Jung, Shuosheng Zhang

**Affiliations:** ^1^ College of Chinese Materia Medica and Food Engineering, Shanxi University of Chinese Medicine, Jinzhong, Shanxi, China; ^2^ Shanxi Key Laboratory of Traditional Herbal Medicines Processing, Shanxi University of Chinese Medicine, Jinzhong, Shanxi, China; ^3^ Faculty of Chinese Medicine, Macau University of Science and Technology, Macau, China; ^4^ School of Chinese Materia Medica, Nanjing University of Chinese Medicine, Nanjing, China; ^5^ School of Pharmacy, Nanjing University of Chinese Medicine, Nanjing, China; ^6^ Shandong Modern University, College of Pharmacy, Jinan, China; ^7^ Department of Herbology, College of Korean Medicine, Dongguk University, Gyeongju, Republic of Korea

**Keywords:** *Rehmannia glutinosa* (Gaertn.) DC., ajugol, isoacteoside, anti-Alzheimer’s disease, serum pharmacochemistry

## Abstract

**Background:**

Alzheimer’s disease (AD) is a progressive neurodegenerative disorder characterized by the deposition of senile plaques, neurofibrillary tangles, and neuronal dysfunction, resulting in severe cognitive and memory decline. The root of the Scrophulariaceae plant *Rehmannia glutinosa* (Gaertn.) DC. (Rehmanniae radix; RR) and its product Rehmanniae radix praeparata (RRP) possess high nutritional and medicinal value. Both show therapeutic potential for AD in traditional medical settings. However, the differences in their bioactive components and the mechanisms of action underlying their anti-AD effects remain unclear.

**Methods:**

In this study, APP/PS1 mice were used as the animal model of AD. Ultra-high-performance liquid chromatography coupled with Q-Exactive tandem mass spectrometry (MS/MS) (UPLC–QE-MS/MS), network pharmacology, proteomics, molecular docking, and 16S rRNA sequencing were used to investigate the differences in the medicinal components of RR and RRP and their mechanisms of action in the treatment of AD. The mechanisms of action of two identified critical components, ajugol and isoacteoside, were further verified in the D-galactose/AlCl_3_-induced Institute of Cancer Research (ICR) mouse model of AD—with cognitive function evaluated using the Morris water maze and open-field tests—and the amyloid-beta (Aβ)-induced BV2 cell model of inflammation.

**Results:**

Ajugol and isoacteoside were identified as the key anti-AD bioactive compounds in RR and RRP, respectively, through UPLC–QE-MS/MS. Integrated network pharmacology, proteomics, and 16S rRNA sequencing implicated neuroinflammation, apoptosis, and autophagy as critical pathways for their anti-AD effects. Subsequently, *in vivo* and *in vitro* experiments demonstrated that ajugol exerted its effects mainly by modulating the TLR/NF-κB/NLRP3 and BCL-2/BAX/cytochrome C/caspase-3 pathways, while isoacteoside primarily acted *via* the LC3-Ⅱ/P62/p-mTOR/mTOR pathway. Ajugol and isoacteoside mitigated cognitive impairment in AD models, decreased Aβ plaque accumulation in hippocampal tissues, and attenuated inflammatory injury-induced cytotoxicity in BV2 microglia, thereby suppressing AD progression.

**Conclusion:**

In this work, we systematically elucidated the differential mechanisms underlying the anti-AD effects of ajugol and isoacteoside. We found that ajugol primarily acts *via* the TLR/NF-κB/NLRP3 and BCL-2/BAX/cytochrome C/caspase-3 pathways, while isoacteoside acts *via* the LC3-II/P62/p-mTOR/mTOR pathway. These findings establish a foundation for developing RRP-based complementary medicines and functional foods.

## 1 Introduction

Alzheimer’s disease (AD) is a neurodegenerative disease with a complex and unclear pathology. Currently, three possible pathological mechanisms have been confirmed for AD, namely, amyloid-β (Aβ) plaques (Ishii et al., 2024), tau hyperphosphorylation, leading to the formation of intracellular neurofibrillary tangles (Varesi et al., 2022), and the loss of neuronal function (Park et al., 2025). Clinically, AD manifests as memory decline and cognitive dysfunction (Cui et al., 2025). According to Alzheimer’s Disease International (ADI), a new case of AD is diagnosed globally every 3 seconds. Currently, more than 50 million elderly individuals worldwide suffer from dementia, a number projected to exceed 152 million by 2050, with AD accounting for 60%–70% of all cases (González et al., 2022). In China alone, at least 300,000 new AD cases are reported annually, representing approximately one-quarter of the global total, and both incidence and mortality continue to increase with population aging (Ji et al., 2024).

AD is a progressive pathological process, and early diagnosis and timely intervention are critical for its treatment. At present, clinical treatment primarily relies on donepezil, a cholinesterase inhibitor, and memantine, a glutamate receptor antagonist. Although these drugs can alleviate the symptoms or delay disease progression, they are associated with adverse effects. Consequently, there is an urgent need to identify novel, multi-target therapeutic agents with well-defined mechanisms of action (Sharma G. K. et al., 2024; Zhang et al., 2024).

AD is classified as a form of “dementia” in traditional Chinese medicine (TCM). Its etiology and pathogenesis are attributed to insufficient bodily nourishment, leading to “deficiencies” in solid organs such as the brain, kidney, and heart. This, in turn, results in neural malnutrition and comprehensive metabolic inflammation, manifesting as cognitive decline, loss of self-care, and physical and verbal aggression (Burton, 2010). Natural products, with their multi-target effects, offer advantages for addressing the complexity of AD pathology. Research on AD treatment has shown that compounds such as flavonoids (Siddiqui et al., 2024b), phenolic acids, stilbenes, coumarins (Hussain et al., 2024), terpenoids, and alkaloids have multi-target synergy, neuroprotective effects, and low toxicity, providing a promising approach for the treatment of AD (Siddiqui et al., 2024a; Tripathi et al., 2024; Hussain et al., 2025). The root of the plant *Rehmannia glutinosa* (Gaertn.) DC. (family: Scrophulariaceae) has high nutritional and medicinal value (Zhang et al., 2022; Ta et al., 2024). It was first recorded in *Shen Nong’s Classic of the Materia Medica* (25–220 C.E.) and was classified as a top-grade herb. Both the raw (Rehmanniae radix, RR) and processed (Rehmanniae radix praeparata, RRP; prepared by steaming RR with rice wine) forms of the root have long been used in TCM for the treatment of AD (Lee et al., 2011; Zhang et al., 2013; Su Y. et al., 2023). According to the TCM pharmaceutical theory, RR has a clearing nature, whereas RRP displays a tonifying nature. Recent studies have reported that the primary active compounds of RR, such as catalpol (Tian et al., 2025), verbascoside (Wang et al., 2020; Chen S. et al., 2022), and rehmannioside A (Fu et al., 2022), exert their AD-mitigating effects by mediating anti-inflammatory processes (Choi, 2019), downregulating pro-inflammatory mediators (Yang et al., 2020), protecting the integrity of the blood–brain barrier (Liu et al., 2018), and upregulating the levels of the neurotrophic factors in the central nervous system. The main active compounds of RRP, 5-hydroxymethylfurfural (5-HMF) and RRP polysaccharides, have been reported to treat AD by modulating immune responses (Zhou et al., 2021), enhancing the anti-oxidant ability of brain cells (Zhang W. et al., 2021), promoting the transformation of bone marrow mesenchymal stem cells into neuron-like cells, regulating synaptic function (Gong et al., 2019), and, ultimately, delaying aging (Feng et al., 2020; Gong et al., 2021). However, research on the differences in the bioactive compound composition of RR and RRP and their potential biological mechanisms of action in AD treatment remains limited, which hinders their rational clinical application and product development.

Given this knowledge gap, we adopted a research approach involving serum/tissue metabolomics using the APP/PS1 mouse model of AD. Ultra-high-performance liquid chromatography coupled with Q-Exactive tandem mass spectrometry (MS/MS) (UPLC–QE-MS/MS) was used to authenticate the bioactive constituents of RR and RRP and their bioactive compounds and metabolites in the serum and brain tissue. Integrated multi-omics analyses, including network pharmacology and proteomics, revealed the potential mechanisms through which RR and RRP ameliorate AD. 16S rRNA sequencing was used to investigate alterations in the composition of the gut microbiota after intragastric administration of RR and RRP, and correlations among bioactive compounds, differential proteins, and microbiota were analyzed. An *in vivo* D-galactose/AlCl_3_-induced model of AD was established in Institute of Cancer Research (ICR) mice, while an Aβ-induced model of inflammation was built *in vitro* using mouse BV2 microglial cells. These models were used to validate the differences in bioactive compound contents and the biological mechanisms of action of the two forms in the treatment of AD. In this study, employing serum pharmacochemistry and systems biology, we comprehensively clarified the differential composition of bioactive compounds in RR and RRP, along with the potential biological mechanisms by which they exert their anti-AD effects. Our findings lay the groundwork for harnessing the full therapeutic potential of RR and RRP and developing safer, more effective complementary AD treatments and functional foods.

## 2 Materials and methods

### 2.1 Extraction of aqueous solutions from RR and RRP

RR and RRP (Batch Nos: 220302 and 220401, Shanxi Yuanhetang Traditional Chinese Medicine Co., Ltd., Shanxi, China) were verified by Professor Shuosheng Zhang of Shanxi University of Chinese Medicine as being the dried tuberous roots of the Scrophulariaceae plant *R. glutinosa* (Gaertn.) DC. The sample specimens were stored in the herbarium of Shanxi University of Chinese Medicine under the specimen numbers SXTCM-Zhang-2022003 and SXTCM-Zhang-2022004.

Based on the previous work by our group, the optimal procedure for stewing RRP in wine involves adding 60 kg of rice wine to 100 kg of raw RR, allowing for 12 h of infiltration. The mixture is then simmered for 38 h and dried for 33 h at 76 °C (Meng et al., 2016; Wang et al., 2019). For both RR and RRP, 100 g of material was weighed. Distilled water (200 mL, equivalent to twice the material’s weight) was added, and the mixture was allowed to soak for 30 min. Subsequently, an additional 800 mL of distilled water (eight times the weight) was added, and the mixture was heated under reflux for 40 min before being filtered. The remaining residue was subjected to a second reflux extraction with 600 mL of distilled water (six times its weight) under heating for another 40 min, followed by filtration. The filtrates from both steps were combined, and the combined filtrate was concentrated under reduced pressure at 50 °C, yielding aqueous extracts of RR and RRP at a concentration of 1 g/mL (Meng et al., 2021). The extracts were stored at 4 °C for subsequent *in vivo* experiments. HPLC was used to determine the levels of catalpol (CAS No. 2415-24-9, Sigma-Aldrich, United States) and ajugol (CAS No. 52949-83-4, Sigma-Aldrich, United States) in the aqueous extract of RR, which were found to be 1.32 ± 0.14 and 0.27 ± 0.01 mg/g, respectively. The contents of 5-HMF (CAS No. 67-47-0, Sigma-Aldrich, United States) and isoacteoside (CAS No. 61303-13-7, Sigma-Aldrich, United States) in the aqueous extract of RRP were 0.89 ± 0.12 and 0.03 ± 0.01 mg/g, respectively.

### 2.2 Experimental animals and cells

For the analysis of the bioactive compounds of RR and RRP in the serum and brain tissue potentially associated with anti-AD effects, male C57BL/6J and APP/PS1 mice (9 months old, weighing 28 ± 3 g) of specific pathogen-free (SPF) grade were used. These mice were sourced from SPF (Beijing) Biotechnology Co., Ltd. (Beijing, China) under the Animal License Number SCXK (Beijing) 2019-0010.

For *in vivo* validation of the bioactive effects of RR and RRP and to investigate differences in their mechanisms of action relating to their anti-AD effects, male ICR mice (SPF grade, aged 4–5 weeks, and weighing 20 ± 3 g) were used. These mice were obtained from Charles River (Beijing) Laboratory Animal Technology Co., Ltd. (Beijing, China) under the Animal License Number SCXK (Beijing) 2021-0006.

Ethical approval for the experiments involving animals was obtained from the Ethics Committee of Shanxi University of Chinese Medicine (Approval No.: AWE202302025; effective from 16 June 2023 to 10 April 2024). The study adhered to the *Animal Research: Reporting of In Vivo Experiments* (ARRIVE) guidelines.

BV2 mouse microglial cells (Lot: IS95YCCGHJ, Procell Life Science and Technology Co., Ltd., Wuhan, Hubei, China) were cultured in Dulbecco’s modified Eagle’s medium (DMEM) (Cat. no. 8122691, Gibco) supplemented with 10% fetal bovine serum (FBS; Cat. no. 10099-141C, Gibco) and 1% penicillin–streptomycin (Lot: MA0110-Nov-10G, Dalian Meilun Biotechnology Co., Ltd., Dalian, Liaoning, China) at 37 °C in an incubator with 5% CO_2_. The BV2 cells were used for the *in vitro* validation of the bioactive compounds of RR and RRP and to study the differences in their mechanisms of action in AD treatment.

### 2.3 Establishment of the mouse models and the administration of RR and RRP

#### 2.3.1 Establishment of the APP/PS1 mouse model

The mice were allowed 1 week of adaptive feeding. APP/PS1 mice were randomly assigned to the model, model + RR (15 g/kg/day), and model + RRP (15 g/kg/day) groups, with 10 mice per group (15 g/kg/day in mice corresponds to 1,662 mg/kg in humans, or 116.4 g per 70 kg BW). Aqueous extracts of RR and RRP were administered *via* gavage for four consecutive weeks. The control and model animals were given an equal volume of purified water. C57BL/6J mice served as the controls. After the administration period, the mice were euthanized. Blood was collected from the abdominal aorta of the mice, centrifuged at 7,104 × *g* for 15 min at 4 °C, and the resulting serum was stored at −80 °C for later use. Brain tissues and feces were fixed in 4% formaldehyde and stored at −80 °C until use. These samples were used to study the chemical compounds of RR and RRP and their roles in preventing AD symptoms, identify their bioactive compounds n serum and brain tissue, and perform 16S rRNA sequencing and proteomic analysis.

#### 2.3.2 Establishment of the ICR mouse model

Mice were allowed 1 week of adaptive feeding. Subsequently, ICR mice were randomly categorized into the following groups, with *n* = 10/group: control, model, donepezil (0.65 mg/kg/day) (Zhang et al., 2019), ajugol-high/low (H/L) (100/50 mg/kg/day) (Zhang H. et al., 2021), isoacteoside-H/L (5/2.5 mg/kg/day) (Shiao et al., 2017), RR-H/L (15/5 g/kg/day), and RRP-H/L (15/5 g/kg/day). A rapid-aging AD model was established *via* induction with D-galactose (60 mg/kg/day; Cat. No. 59-23-4, Sigma-Aldrich, United States) and AlCl_3_ (100 mg/kg/day; Cat. No. 237051, Sigma-Aldrich, United States) (Impellizzeri et al., 2023) for 8 weeks. After 4 weeks of modeling, treatments were administered by gavage to each group once daily for 4 weeks, with an interval of more than 8 h between administrations. The general condition of the mice was monitored daily throughout the experimental period.

### 2.4 Identification of RR and RRP compounds in the serum and brain tissue by UPLC–QE-MS/MS

After centrifugation (13,400 × *g*, 15 min), the supernatants of the RR and RRP extracts were filtered through a 0.22 μm filter membrane for UPLC–QE-MS analysis.

Serum sample preparation: APP/PS1 mice were intragastrically administered RR and RRP at a dose of 15 g/kg. Serum was collected from the mice and used for subsequent analyses after sample pretreatment.

Preparation of brain tissue samples: A total of 0.2 g of APP/PS1 mouse brain tissue was transferred to a homogenization tube, to which 1 mL of 70% methanol solution was added. The tissues were homogenized and centrifuged (13,400 × *g*, 15 min). Subsequently, 800 μL of the supernatant was collected, and the residues were vacuum dried before 160 μL of a 40% methanol aqueous solution was added to the residue. After mixing by vortexing and centrifugation, the supernatant was collected (Liu et al., 2025; Qin et al., 2025).

LC–MS/MS analysis: The chemical constituents of RR and RRP, along with their compounds in the serum and brain tissues, were identified according to previously described methods (Chen Y. et al., 2022). Analysis was performed using a Thermo UPLC Vanquish system (Thermo Fisher Technology Co., Ltd., CA, United States) coupled with a Q-Exactive HF-X Hybrid Quadrupole-Orbitrap Mass Spectrometer (Thermo Fisher Technology Co., Ltd., United States). MS and LC–MS/MS data were also collected using a Q-Exactive Focus mass spectrometer. The instrument was controlled by Xcalibur (v.3.1) software and operated in IDA mode. Data from UPLC–QE-MS/MS were processed using Compound Discoverer (v.3.2) software (Thermo Fisher Scientific, Waltham, MA, United States).

### 2.5 Network pharmacology and compound–target molecular docking analysis

Based on the compounds identified in Section 2.4 from the serum and brain tissues following RR and RRP administration in APP/PS1 mice, a comparative analysis was conducted to evaluate the bioactive compounds of RR and RRP within the therapeutic network for AD treatment and identify the differences in their biological pathways (Subramanian et al., 2024; Yu et al., 2024). Potential compound targets were retrieved from the TCMSP (Ru et al., 2014), TCMID (Xue et al., 2013), and CTD (Davis et al., 2025) databases. Relevant AD-related targets were obtained from CTD using “AD” as the search keyword. All targets were standardized to the gene names *via* UniProt (Wang et al., 2021). Overlapping disease and compound targets were identified using Venn diagram analysis. Key disease-associated targets (gene degree > median) were further analyzed using the STRING database (Szklarczyk et al., 2021), and a protein–protein interaction (PPI) network was constructed. The top 50 targets were then visualized in a network diagram using NetworkX (Sharma P. et al., 2024) in Python.

GO (Ashburner et al., 2000) and KEGG (Kanehisa and Goto, 2000) enrichment analyses were conducted on the core targets of RR and RRP. The results were visualized using the R language. A “Key Component–Core Target–Pathway” network integrating the component–target data, the top 20 KEGG pathways, and the RR/RRP prototype components in the serum and brain tissue was built using Cytoscape (v.3.7.1). The therapeutic components and their differential biological pathways linked to anti-AD effects were comparatively analyzed.

Molecular docking (AutoDock Vina v.1.2.2) (Yang L. et al., 2024) was used to assess binding energy and interactions between the candidate compounds (ajugol and isoacteoside) and targets (TLR4/3VQ1, NFKBIA/6Y1J, NLRP3/7VBF, BCL-2/1NMQ, CASP3/6GL8, P62/2FAP, and MTOR/6KHZ). Compound structures were retrieved from PubChem (Wu D. et al., 2024), while the target structures were obtained from the Research Collaboratory for Structural Bioinformatics (RCSB) PDB. Proteins and ligands were converted to the PDBQT format, followed by water removal and polar hydrogen addition. A 30-Å grid box was used for docking (0.05-nm spacing). The optimal binding poses were selected based on energy and conformation.

### 2.6 Analysis of the gut microbiota in APP/PS1 mice

The V3–V4 hypervariable region of the bacterial 16S rRNA gene was PCR-amplified, and library preparation, along with quality control, was carried out using the TruSeq Nano DNA LT Library Prep Kit. Library sequencing was performed on the Illumina Nova high-throughput platform. The resulting sequences were analyzed using QIIME software (v1.8.0). High-quality reads were processed with UCLUST to remove chimeric sequences, after which de-duplicated sequences were clustered into operational taxonomic units (OTUs) at a 97% similarity threshold. Based on OTU clustering, the relative abundances of different taxonomic levels were determined. Furthermore, analyses of α-diversity, β-diversity, and OTU-based Venn diagrams were conducted to explore the microbial composition differences among samples. Functional gene profiles were inferred by comparing species compositions obtained from 16S rRNA sequencing data using Phylogenetic Investigation of Communities by Reconstruction of Unobserved States 2 (PICRUSt2) software (Liao et al., 2024; Zeng et al., 2024). This allowed for the assessment of functional differences between the groups.

### 2.7 Proteomic analysis of APP/PS1 mice

Peptides from the brain tissues of APP/PS1 mice were quantified using LC–MS analysis methods that were previously established by our group. Data analysis was performed based on both the fragment ion distribution for peptide scoring and the protein abundance ratios. Approximately 98.27% of the peptides achieved scores above 20, and most protein abundance ratios across the three groups of equally labeled samples were close to 1, indicating high identification accuracy and reliable experimental quality. Hierarchical clustering analysis was applied to all differentially expressed proteins, and corresponding volcano plots and heatmaps were generated. In addition, GO functional annotation and KEGG pathway analysis were conducted (Wu Q. et al., 2024; Sun et al., 2025).

### 2.8 Behavioral tests in ICR mice

Spatial memory in the various groups of ICR mice was estimated through the Morris water maze test. Using methods previously developed by our group, the time it took each mouse to find the hidden platform (escape latency), the number of times each mouse crossed the area where the platform had previously been located within 300 s, the time spent in the target quadrant, and the swimming velocity were calculated (Hu et al., 2024). All experimental results were recorded using a Morris water maze test system (WMT-200A; Chengdu Techman Software Co., Ltd., Chengdu, China).

The open-field test was used for estimating the spatial exploration capacity of the ICR mice in each group. A rectangular box measuring 50 cm (width) × 50 cm (length) × 38 cm (height) was divided into nine areas (one central and eight peripheral) for the open-field tests. During the tests, mice from each group were placed at the bottom center of the box, always at the same angle, followed by video recording and timing, with each mouse being tested for 300 s each time. Indicators included the total time spent moving in the open-field, the time spent in the corner areas, and the time spent in the central zone (Su S. et al., 2023). All the experimental results were recorded using a rat open-field test system (OFT-200A; Chengdu Techman Software Co., Ltd., Chengdu, China).

The ICR mice were euthanized following the completion of the behavioral experiments. Blood was collected through the abdominal aorta and centrifuged, and the resulting serum was stored at −80 °C. Excised hippocampal tissues (Meng et al., 2022) were fixed in 4% formaldehyde or stored at −80 °C for the differential analysis of bioactive compounds of RR and RRP and the potential biological mechanisms underlying their anti-AD activity.

### 2.9 Thioflavin-S staining of hippocampal tissue of ICR mice

Aβ plaque density in the hippocampal tissue was determined using thioflavin-S staining (Rajamohamedsait and Sigurdsson, 2012; Li et al., 2025). After fixing overnight in paraformaldehyde, hippocampal tissues were dehydrated in 30% sucrose solution, paraffin-embedded, cut into 20-μm sections, deparaffinized using xylene, and immersed in a series of ethanol solutions (100%, 95%, 80%, and 70% for 1 min each). Slices were placed in water for a few seconds and then stained with 1% thioflavin-S solution at 25 °C for 30 min. The sections were then immersed in ethanol solution (70%, 80%, 95%, and 100% for 1 min each time), cleared in xylene for 5 min, sealed in neutral balsam, and examined under a fluorescence microscope (Nikon Ti-S; Tokyo, Japan). Thioflavin-S staining was performed under ultraviolet light.

### 2.10 MALDI–MSI analysis of the brain hippocampus tissue in ICR mice

Hippocampal tissue sections (20 μm) from the control, model, ajugol-H, and isoacteoside-H groups (*n* = 3) were prepared using a Leica CM1950 cryostat and mounted on ITO slides for MALDI–MSI. Sections were vacuum-dried for 30 min and stored at −80 °C. A 5 mg/mL NEDC matrix solution (70% methanol and 3.4% ammonia) was applied using an M3 TM-sprayer under the following conditions: velocity, 1,200 mm/min; spacing, 3 mm; flow rate, 0.08 mL/min; temperature, 60 °C; N_2_ pressure, 10 psi. Imaging was performed on a timsTOF fleX MALDI 2 system in negative ion mode with the following settings: *m*/*z* range, 100–1,200; spatial resolution, 50 μm; laser energy, 70%; 200 shots per pixel.

MSI data were processed using SCiLS Lab 2021c with RMS normalization and matched against the Bruker MS-MetaboBASE 3.0 (*m*/*z* tolerance <10 ppm). Significant metabolites were identified based on ROC analysis (AUC >0.7 or <0.3) and the Student’s t-test with multiple testing correction (*p* < 0.01) (Wang et al., 2022).

### 2.11 Establishment of the BV2 microglial cell model of inflammation and the administration of various compounds

BV2 cells were seeded in 96-well plates at a density of 5 × 10^4^ cells/mL. Changes in BV2 cell viability at various compound concentrations (0.5, 1, 2, 5, 10, 20, and 40 µM) and under Aβ_1–42_ exposure (Ma et al., 2022) (CAS No. 107761-42-2, Macklin, Shanghai, China) were detected using the Cell Counting Kit (CCK)-8. Sampling was performed at 0, 12, 24, 36, 48, 60, and 72 h. In addition, the cell culture supernatant was collected, and the contents of COX-2, IL-1β, iNOS, and IL-4 (Nos F2356-A, F2040-A, F2454-A, and F2165-A; Kexing Trading Co., Ltd., Shanghai, China) were quantified using the enzyme-linked immunosorbent assay (ELISA) to determine the optimal Aβ_1–42_ concentration and modeling time.

For the administration of the various compounds, BV2 cells were seeded at the same density (5 × 10^4^ cells/mL) in 96-well plates. Different concentrations of ajugol (0, 5, 10, 25, 50, 100, 150, and 200 µM) and isoacteoside (0, 5, 10, 20, 40, 80, 100, and 150 µM) (Li et al., 2018) were administered 24 h before Aβ_1–42_ intervention. The cells were assigned to the control, model, ajugol-H, ajugol-L, isoacteoside-H, and isoacteoside-L groups. The control group received no treatment, whereas the model groups were exposed to Aβ_1–42_. The effects of different drug concentrations on the viability of BV2 cells *in vitro* were detected using the CCK-8 assay.

### 2.12 ELISA assays in BV2 microglial cells

The concentrations of IL-1β, IFN-γ, IL-4, NO, and G-CSF (Nos F2040-A, F2182-A, F2165-A, F30040-A, and F2186-A; Kexing Trading Co., Ltd., Shanghai, China) in the BV2 cell culture supernatant were estimated using ELISA kits following the provided instructions.

### 2.13 Immunofluorescence analysis of BV2 microglial cells

After treatment, BV2 cells from each group were fixed in 4% paraformaldehyde/phosphate-buffered saline (PBS) (pre-warmed to 37 °C) for 15 min, washed three times in PBS, permeabilized in 0.5% Triton X-100, blocked with 2% BSA for 1 h, and incubated overnight at 4 °C with primary antibodies targeting p-NF-κB (p65) (1:200), NF-κB (p65) (1:200), cytochrome C (1:200), and P62 (1:200) (Nos AP0475, A2547, A13430, and A19700; ABclonal Technology Co., Ltd., Wuhan, China) in blocking solution. After three washes with PBS, the cells were incubated with fluorescent secondary antibodies for 45 min at room temperature and then imaged using a confocal microscope (Leica Biosystems, Wetzlar, Germany) (Wu et al., 2023; Chen et al., 2025).

### 2.14 Western blotting analysis

Total protein was extracted from the hippocampal tissue of ICR mice and BV2 cells using RIPA lysis buffer. Protein concentrations were determined using the Bradford assay. After separation by SDS-PAGE, proteins were transferred to a PVDF membrane, blocked for 2 h at room temperature, and incubated overnight at 4 °C with primary antibodies against TLR4, p-NF-κB (p65), NF-κB (p65), p-IκBα, IκBα, NLRP3, caspase-1, cytochrome C, BAX, BCL-2, caspase-3, LC3-II, P62, p-mTOR, mTOR, and β-actin (Nos A5258, AP0475, A2547, AP0707, A19714, A5652, A0964, A1561, A0207, A0208, A2156, A5618, A19700, AP-0094, A2445, and AC038, respectively; ABclonal Technology Co., Ltd.). Following three washes with Tris-buffered saline containing Tween (TBST), the membranes were incubated with HRP-conjugated rabbit secondary antibodies (No. AS014; ABclonal Technology Co., Ltd.) at 25 °C for 3 h. The protein bands were imaged using the GeneGnome imaging system (GeneGnome XRQ, Gene, United States), and the relative expression levels were determined using ImageJ software (ImageJ2, NIH, United States) (Fu et al., 2025; Zheng et al., 2025).

### 2.15 Statistical analysis

All data were analyzed in GraphPad Prism (v.9.0; GraphPad Software, La Jolla, CA, United States) and are presented as the means ± standard error of the mean (SEM) from three separate experiments. For comparisons between two groups, the Student’s t-test was used to assess the significance of differences. When three or more groups were compared, one-way analysis of variance (ANOVA) was performed, followed by Tukey’s *post hoc* test. *P* < 0.05 was considered statistically significant.

## 3 Results

### 3.1 Differential analysis of prototype compounds in the serum and brain tissue of APP/PS1 mice treated with RR and RRP

UPLC–QE-MS/MS was used to authenticate the bioactive compounds in the serum and brain tissue and the metabolites of RR and RRP. Consistent with previous studies, the detected RR and RRP compounds included the iridoid glycosides such as catalpol and aucuboside, terpenoids, flavonoids, lignans, and various polysaccharides (Chen H. et al., 2022; Bian et al., 2023; Jia et al., 2023). In the base peak chromatogram (BPC) of RR, 26 chromatographic peaks were identified, with 15 peaks detected in positive ion mode and 11 in negative ion mode. Similarly, the BPC of RRP authenticated 31 chromatographic peaks, including 12 peaks detected in positive ion mode and 19 in negative ion mode (Supplementary Table S1, S2; Supplementary Figure S1(A)1–4).

After excluding the intrinsic serum compounds, chromatograms from RR- and RRP-treated mice were compared with those of the control group to identify chemical constituents that are potentially relevant for anti-AD treatment (Supplementary Figures S1(A)-5A,B). Compounds were selected based on the peak response intensity, with the criterion that the intensity in the model group increased more than threefold after administration compared to that during pre-administration.

Ajugol, mussaenosidic acid, 3-hydroxy-3-methyl-5-oxo-5-(((2R,3S,4S,5R,6R)-3,4,5-trihydroxy-6-(octan-3-yloxy)tetrahydro-2H-pyran-2-yl)methoxy)pentanoic acid, and *cis*-pinonic acid were detected in the serum of both RR- and RRP-treated mice. RR-specific compounds included L-1,2,3,4-tetrahydro-beta-carboline-3-carboxylic acid, sesamoside, epiligulyl oxide, tetrahydroharman-3-carboxylic acid, succinic acid, and hexylsuccinic acid. In contrast, RRP-specific compounds comprised melibiose, glutamic acid, ZINC67902872, [(2R,3R,4S,5R,6R)-6-[2-(3,4-dihydroxyphenyl)ethoxy]-3,5-dihydroxy-4-[(2R,3R,4R,5R,6S)-3,4,5-trihydroxy-6-methyloxan-2-yl]oxyoxan-2-yl]methyl (E)-3-(3,4-dihydroxyphenyl)prop-2-enoate, ketologanic acid, 5-(hydroxymethyl)-2-furoic acid, alpha-isopropylmalate, loganin, 3-methoxytyrosine, juniperoside III, isoacteoside, 3-(N-maleimidopropionyl)biocytin, and (9E,11Z)-8-hydroxyoctadeca-9,11-dienoic acid (Table 1).

**TABLE 1:** Comparison of the components of RR and RRP in the serum and brain tissue.

Item	*m*/*z*	RT/min	ppm	Compound name	RR	RRP
Identification of components in the serum	393.1404	2.79	1.1	Ajugol	In serum	In serum
	375.1297	3.19	0.4	Mussaenosidic acid	In serum	In serum
	435.2234	5	3	3-Hydroxy-3-methyl-5-oxo-5-(((2R,3S,4S,5R,6R)-3,4,5-trihydroxy-6-(octan-3-yloxy)tetrahydro-2H-pyran-2-yl)methoxy)pentanoic acid	In serum	In serum
	623.1984	5.45	0.4	ZINC67902872	In serum	In serum
	217.0971	3.91	16.8	L-1,2,3,4-Tetrahydro-beta-carboline-3-carboxylic acid	In serum	None
	385.1102	1.18	6.6	Sesamoside	In serum	None
	213.1234	2.8	19.2	Epiligulyl oxide	In serum	None
	217.0971	3.91	16.8	Tetrahydroharman-3-carboxylic acid	In serum	None
	183.1018	7.74	4.2	*cis*-Pinonic acid	In serum	None
	117.0182	1.36	9.4	Succinic acid	In serum	None
	183.102	5.2	4.2	Hexylsuccinic acid	None	In serum
	373.114	2.34	0.1	Ketologanic acid	None	In serum
	141.0182	2.54	6.1	5-(Hydroxymethyl)-2-furoic acid	None	In serum
	175.0601	3.92	4	Alpha-isopropylmalate	None	In serum
	435.1508	4.17	0.7	Loganin	None	In serum
	210.0767	4.44	0.5	3-Methoxytyrosine	None	In serum
	357.1192	4.63	0.4	Juniperoside III	None	In serum
	623.1982	5.21	0.6	Isoacteoside	None	In serum
	425.1948	5.36	19.5	3-(N-Maleimidopropionyl)biocytin	None	In serum
	183.1019	7.83	4	(9E,11Z)-8-Hydroxyoctadeca-9,11-dienoic acid	None	In serum
	365.105	1.27	0.7	Melibiose	None	In serum
	130.0498	1.96	0.7	Glutamic acid	None	In serum
Identification of components in the brain	393.1403	2.87	0.8	Ajugol	In the brain	In the brain
	623.1982	5.21	0.6	Isoacteoside	None	In the brain
	183.1018	7.74	4.2	cis-Pinonic acid	None	In the brain
	183.102	5.2	4.2	Hexylsuccinic acid	None	In the brain
	192.066	3.53	3	4-(Acetylmethylamino)benzoic acid	None	In the brain
	175.0601	3.92	4	Alpha-isopropylmalate	None	In the brain
	130.0498	1.96	0.7	Glutamic acid	None	In the brain
	435.2235	5.05	0.5	3-Hydroxy-3-methyl-5-oxo-5-(((2R,3S,4S,5R,6R)-3,4,5-trihydroxy-6-(octan-3-yloxy)tetrahydro-2H-pyran-2-yl)methoxy)pentanoic acid	In the brain	None
	623.1982	5.49	0.2	ZINC67902872	In the brain	None
	213.1234	2.8	19.2	Epiligulyl oxide	In the brain	None

A similar criterion was applied for brain tissue analysis, where a compound was considered relevant if its peak intensity increased more than threefold after administration in APP/PS1 mice compared to the pre-administration values. Ajugol was common for both RR and RRP in the brain. Epiligulyl oxide, cis-pinonic acid, and hexylsuccinic acid were unique to RR, while glutamic acid, 3-hydroxy-3-methyl-5-oxo-5-(((2R,3S,4S,5R,6R)-3,4,5-trihydroxy-6-(octan-3-yloxy)tetrahydro-2H-pyran-2-yl)methoxy)pentanoic acid, ZINC67902872, 4-(acetylmethylamino)benzoic acid, alpha-isopropylmalate, and isoacteoside were specific to RRP (Supplementary Figures S1(A)-5C, D; Table 1).

### 3.2 Construction of the “key compound–core target–pathway” differential network diagram between RR and RRP in AD treatment

**FIGURE 1:**
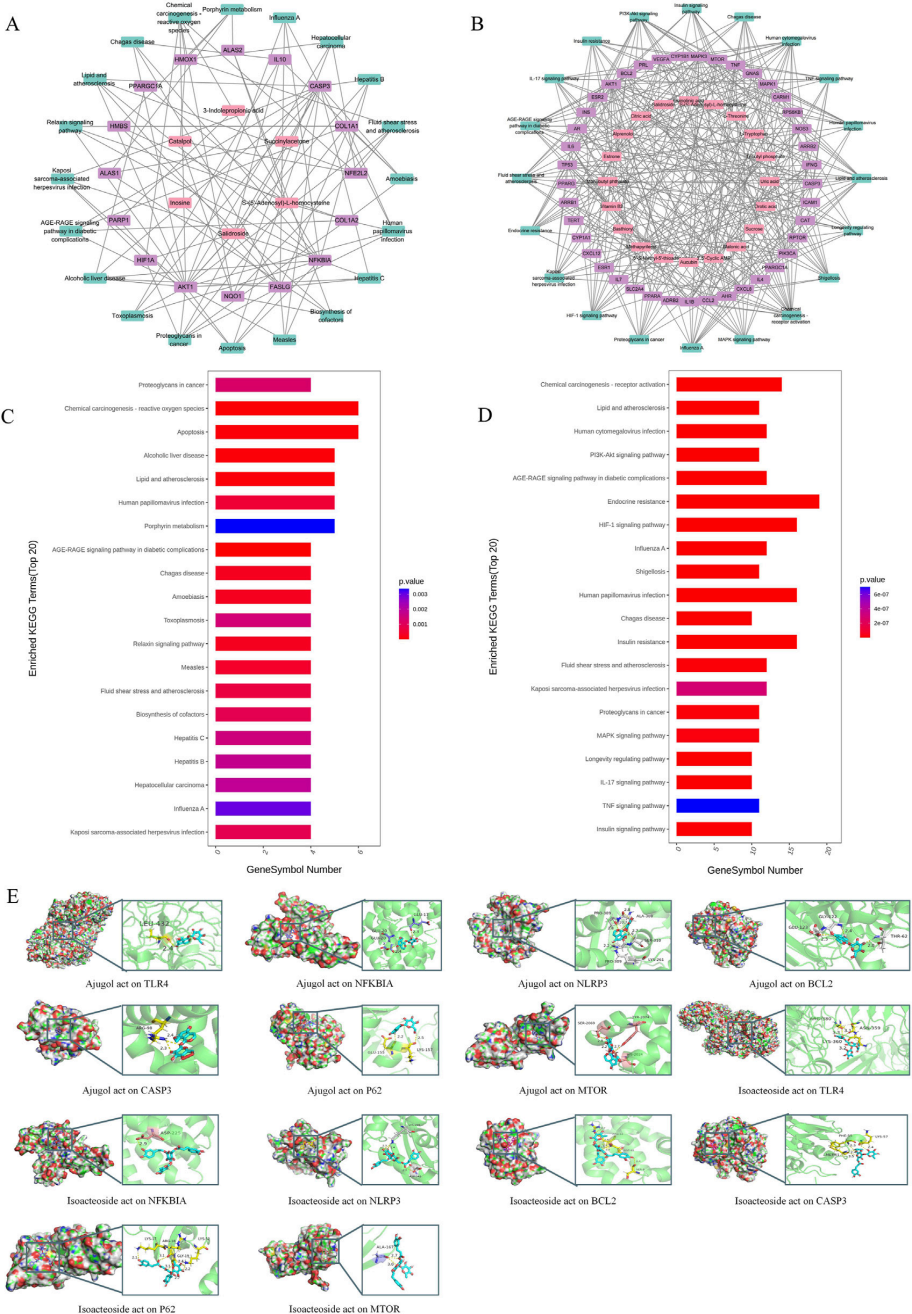
Network and molecular docking analysis of active compounds from RR and RRP in AD treatment. **(A)** Compound–target–pathway network (top 20) of RR in AD treatment. **(B)** Compound–target–pathway network (top 20) of RRP in AD treatment. **(C)** KEGG pathway enrichment map of the active compounds of RR in AD treatment. **(D)** KEGG pathway enrichment map of the active compounds of RRP in AD treatment. **(E)** Molecular docking results of active compounds of RR and RRP (ajugol and isoacteoside) with seven target proteins (TLR4, NFKBIA, NLRP3, BCL-2, CASP3, P62, and MTOR).

Based on the compounds of RR and RRP identified in the serum and brain tissue (Section 3.1), a comparative analysis was performed to evaluate the bioactive components of RR and RRP within the therapeutic network for AD treatment and identify the differences in the associated biological pathways. The network diagram revealed that the bioactive compound network of RRP in AD treatment was more complex than that of RR (Figures 1A, B).

Analysis of the top 20 pathways indicated that RR primarily exerted its effects through mechanisms involving neuronal apoptosis, oxidative stress, and neuroinflammation, including pathways such as chemical carcinogenesis–reactive oxygen species, toxoplasmosis, hepatitis C, and relaxin signaling. In contrast, RRP acted mainly through pathways associated with oxidative stress, immune inflammation, nutrient metabolism, and cell growth, including chemical carcinogenesis–reactive oxygen species, HIF-1 signaling, endocrine resistance, and insulin resistance, thereby influencing AD treatment (Figures 1C, D).

A molecular docking analysis was used to evaluate the affinities of the bioactive compounds of RR and RRP for their targets. The binding conformations and interactions of the two active compounds, ajugol and isoacteoside, with seven proteins (TLR4, NF-κB, NLRP3, BCL-2, caspase-3, P62, and mTOR) were determined using AutoDock Vina. The binding energy for each interaction was also obtained (Figure 1E; Supplementary Table S3), with a smaller binding energy in molecular docking correlating with a stronger affinity between the receptor and the ligand. The hydrophobic pockets of each target were successfully occupied by both candidate compounds. With NF-κB, ajugol demonstrated a low binding energy of −7.799 kcal/mol. For isoacteoside, its binding energies with TLR4, NFKBIA, and NLRP3 were −9.668, −9.031, and −9.668 kcal/mol, respectively, indicating that the interactions were highly stable (Supplementary Table S3). These results indicated that the effective compounds of RR and RRP stably bind to proteins involved in neuroinflammation, apoptosis, and autophagy, suggesting that they offer potential for further research.

### 3.3 Analysis of the effects of RR and RRP on the gut microbiota in APP/PS1 mice

**FIGURE 2:**
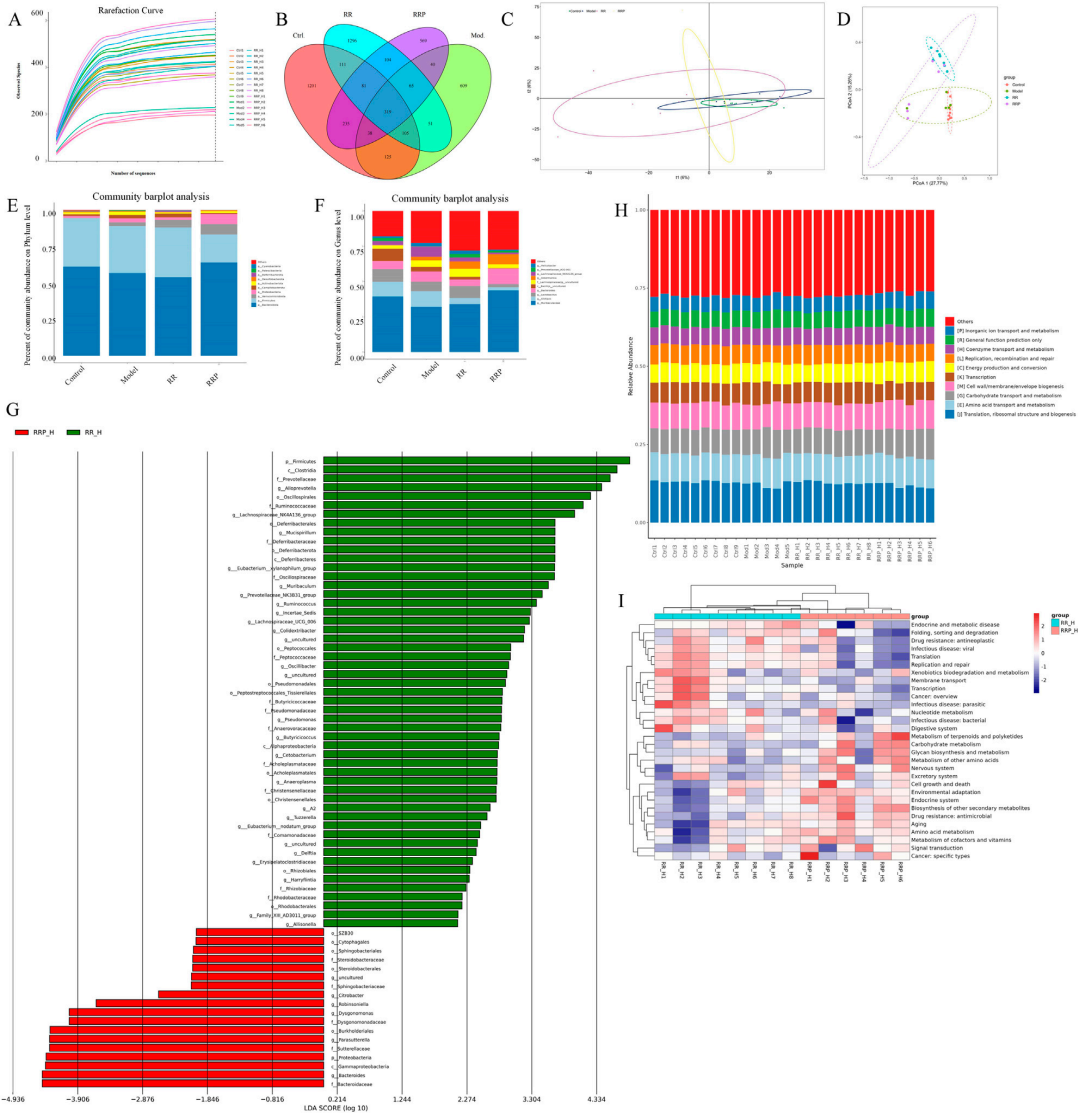
Effects of RR and RRP on the gut microbiota in APP/PS1 mice. **(A)** Rarefaction curve of each sample. **(B)** Venn diagram of the OTUs. **(C)** PCA analysis of each group. **(D)** PCoA analysis of each group. **(E)** Percent community abundance of each group at the phylum level. **(F)** Percent community abundance of each group at the genus level. **(G)** Linear discriminant analysis (LDA) of RR and RRP. **(H)** Bar chart of the relative abundance of COG functional categories. **(I)** KEGG heatmap analysis. The data are expressed as the means ± SEM. *n* = 3 per group.

In analyzing the effects of RR and RRP on the diversity of the intestinal flora in APP/PS1 mice, we observed that the rarefaction curve flattened as the number of reads increased, suggesting that the sequencing depth was adequate to estimate the microbiome composition in each sample. The samples showed a uniform species distribution, and the amount of sequencing data was considered suitable for subsequent analyses (Figure 2A). Sequence clustering at a 97% similarity threshold led to the identification of 4,949 OTUs. Venn diagram analysis indicated that 319 OTUs were common to all four groups (Figure 2B). Alpha-diversity analysis revealed that compared to the model group, the RRP group had a higher coverage index, while the RR group exhibited a higher PD_whole_tree index (Supplementary Figure S4A). Beta-diversity analysis results revealed that post-RR and -RRP administration, the weighted UniFrac, Bray–Curtis, and unweighted UniFrac indices were increased in the RR and RRP groups (Supplementary Figure S4B) relative to those in the model group. PCA (Figure 2C) and PCoA (Figure 2D) demonstrated that there was clear separation among the four groups.

At the phylum level, Verrucomicrobiota, Firmicutes, Bacteroidetes, Proteobacteria, and Campylobacterota accounted for >95% of the total bacteria. Compared with that in the model group, the relative abundance of Verrucomicrobiota was higher in the RR group, whereas that of Proteobacteria was lower. Meanwhile, the RRP group exhibited a higher relative abundance of Bacteroidetes and Verrucomicrobiota and a lower relative abundance of Firmicutes, Proteobacteria, and Campylobacterota than the model group (Figure 2E).

The genera collectively accounting for the top 95% of the relative abundance included *Muribaculum*, *Alistipes*, *Lactobacillus*, *Bacteroides*, *Bacillus*, *Lachnospira*, *Akkermansia*, and *Helicobacter*. Compared with that in the model group, the abundance of *Muribaculum*, *Lactobacillus*, *Lachnospira*, *Akkermansia*, and *Helicobacter* in the RR group was increased by 1.86%, 1.88%, 1.06%, 2.63%, and 0.17%, respectively, whereas that of *Alistipes*, *Bacteroides*, and *Bacillus* was decreased by 6.75%, 2.67%, and 1.37%, respectively. Meanwhile, in the RRP group, the abundance of *Muribaculum, Bacteroides*, and *Akkermansia* was increased by 11.4%, 3.81%, and 4.63%, respectively, relative to the model group, whereas that of *Alistipes*, *Lactobacillus*, *Bacillus*, *Lachnospira*, and *Helicobacter* showed decreases of 8.80%, 4.39%, 2.99%, 2.01%, and 1.59%, respectively. The change in *Alistipes* abundance was statistically significant (*p* < 0.05) (Figure 2F).

Functional enrichment analysis of the gut microbiota (Figures 2G–I) revealed that RRP significantly remodeled microbial metabolic functions in mice. Key enriched pathways included those associated with endocrine and metabolic diseases, alongside terpenoid and polyketide metabolism, suggesting that RRP may influence energy homeostasis through the microbiota–host metabolic axis. The concurrent enrichment of nucleotide metabolism and signal transduction pathways is further indicative of microbiota-mediated regulation of cellular proliferation and communication. Meanwhile, the observed activation of antimicrobial drug resistance and cancer-specific pathways requires additional investigation to elucidate their potential association with host immunomodulation. Collectively, these findings demonstrated that microbiota modulation underpins the superior therapeutic efficacy of RRP in AD treatment.

In summary, interventions with either RR or RRP can increase the diversity and richness of microbiota in the gastrointestinal tract of APP/PS1 mice.

### 3.4 Differential analysis of the proteome of APP/PS1 mice treated with RR and RRP

**FIGURE 3:**
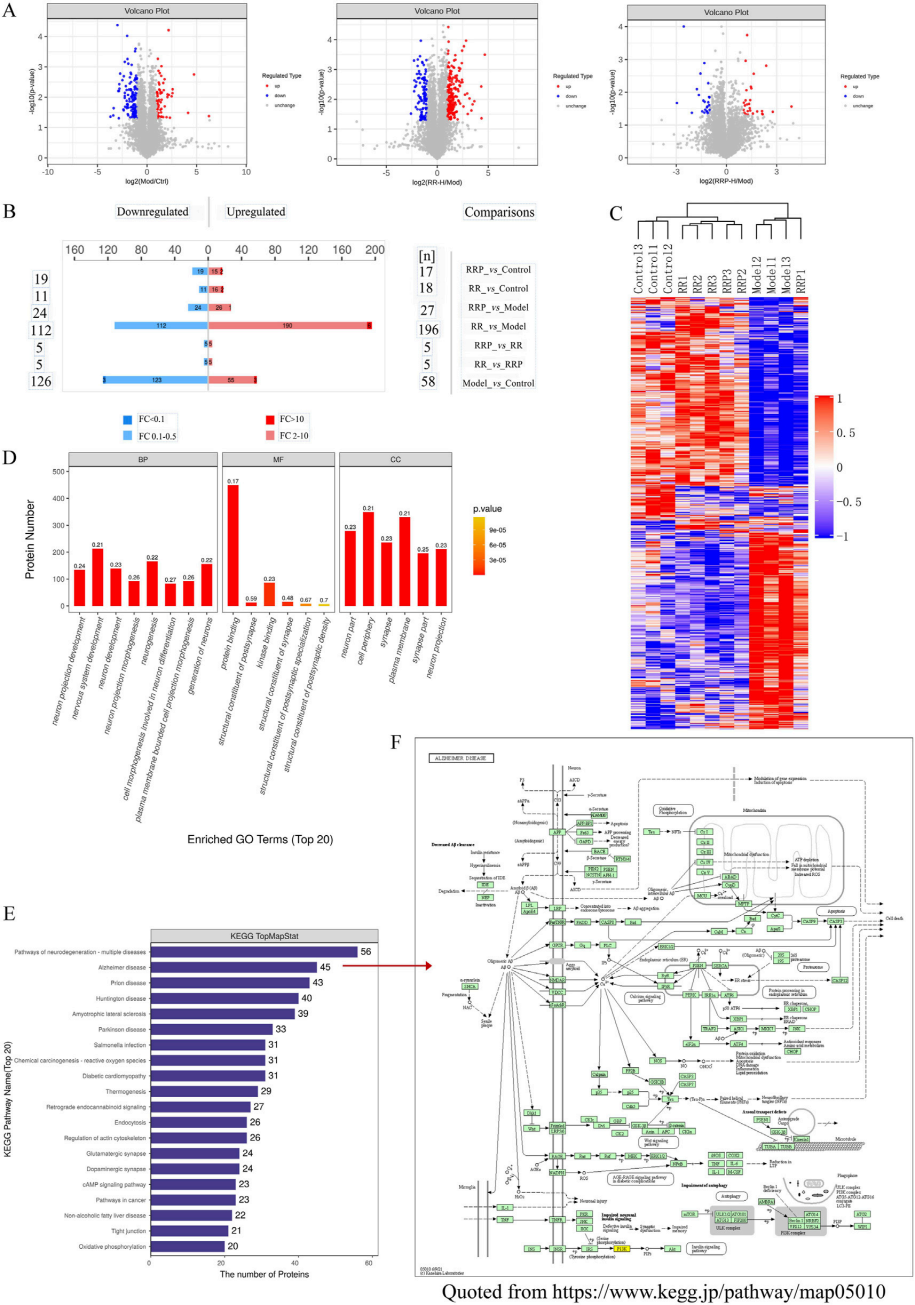
Differential proteomic effects of RR and RRP in APP/PS1 mice. **(A, B)** Volcano plots of differential proteins (*n* = 3). Blue shows the downregulated differentially expressed proteins, red shows the upregulated differentially expressed proteins, and gray shows proteins with no significant difference in expression. **(C)** Hierarchical clustering heatmap of the differentially abundant proteins. **(D)** GO enrichment analysis of the differentially abundant proteins. **(E)** KEGG enrichment analysis of the differentially abundant proteins. **(F)** Alzheimer’s disease pathway (https://www.kegg.jp/pathway/map05010). The data are expressed as the means ± SEM. *n* = 3 per group.

Differential analysis of proteomes from APP/PS1 mice treated with RR or RRP yielded 986,622 secondary spectra, 439,942 of which matched the spectra in the UniProtKB database (https://www.uniprot.org). A total of 52,981 peptides, including 50,901 unique peptides, were identified, along with 5,213 proteins, of which 5,169 were quantifiable, indicating the high quality of protein extraction. Proteins with differential expression among the groups were analyzed using volcano plots (Figure 3A) and heatmaps generated by hierarchical clustering. The results revealed that compared to the model group, the RR group contained 308 differentially expressed proteins (112 upregulated and 196 downregulated), while the RRP group contained 51 differentially expressed proteins (24 upregulated and 27 downregulated) (Figures 3B, C), signifying substantial changes in the protein content within the brain tissue of mice.

GO enrichment analysis was performed on all differentially expressed proteins identified in mouse brain tissue (Figure 3D). KEGG pathway annotation and quantification of the differentially abundant proteins indicated that the top 20 enriched pathways were largely involved in neurodegeneration, multiple diseases, AD, Parkinson’s disease (PD), prion disease, amyotrophic lateral sclerosis, Huntington’s disease, and *Salmonella* infection (Figure 3E). Notably, the differentially expressed proteins in the RR and RRP groups were associated with signaling pathways such as neuroinflammation, autophagy, and apoptosis (Figure 3F). The involvement of these pathways was validated in subsequent experiments.

### 3.5 Correlation analysis

**FIGURE 4:**
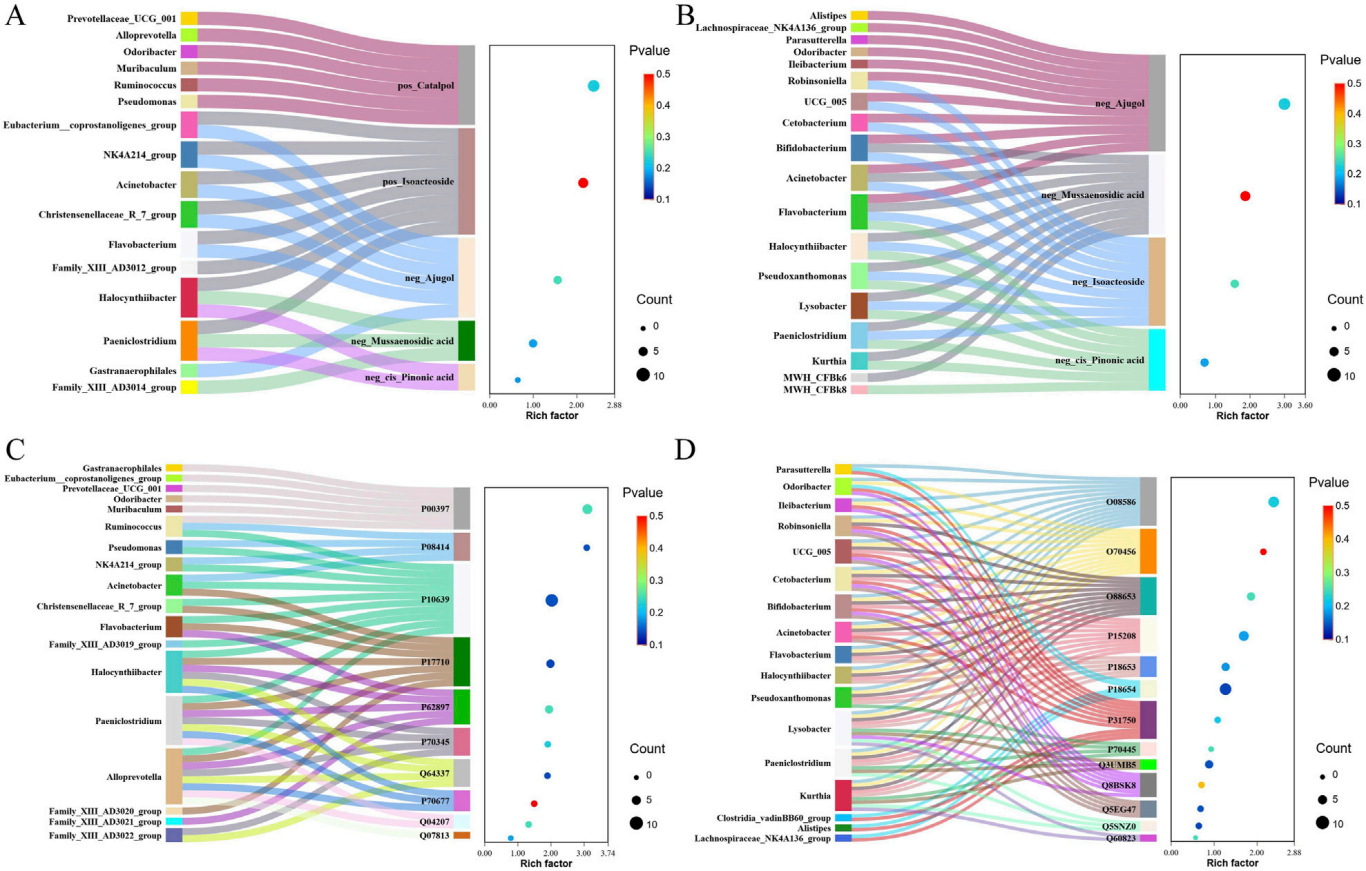
Correlation between differentially abundant microbiota, proteins, and compounds in the serum and brain tissue samples in the treatment of AD with RR and RRP. **(A, B)** Correlation coefficients between differentially abundant microbiota and compounds in the serum and brain tissue in the treatment of AD with RR and RRP. **(C, D)** Correlation coefficient between differentially abundant microbiota and differentially expressed proteins in the treatment of AD with RR and RRP.

Correlation coefficients between differentially abundant microbiota, proteins, and compounds in the serum and brain samples in the treatment of AD with RR and RRP were measured using the R language. The results showed that in the RR group, the strongest correlation among compounds and microbiota was observed between ajugol and *Eubacterium* (Figure 4A), whereas in RRP, the strongest correlations were found between isoacteoside and *Robinsoniella* and between isoacteoside and *Bifidobacterium* (Figure 4B). For proteins and microbiota, the strongest correlation in the RR group was identified between SQSTM1/p62 (Q64337) and *Alloprevotella* (Figure 4C), whereas in the RRP group, the strongest correlation was detected between S6 kinase beta-1 (Q8BSK8) and *Lysobacter* (Figure 4D).

### 3.6 Ajugol and isoacteoside enhanced the cognitive function and mitigated Aβ plaque accumulation in the hippocampal tissue of ICR mice

Ajugol was identified as a common compound in both the serum and brain tissues following the administration of either RR or RRP. Conversely, isoacteoside was detected exclusively in the brain tissue after RRP administration. Consequently, ajugol and isoacteoside were selected as representative bioactive markers for RR and RRP, respectively, for subsequent mechanistic investigations.

The protective effects offered by ajugol and isoacteoside against cognitive deficits in ICR mice were confirmed *via* behavioral experiments. In the Morris water maze test, compared with the controls, mice in the model group showed a markedly prolonged escape latency, along with considerable reductions in platform crossings, time spent in the target quadrant, and swimming speed. Additionally, mice in the model group spent substantially more time in the corner field and significantly less time moving in the central zone in the open-field test. This indicated that the mouse model of AD had been successfully established.

**FIGURE 5:**
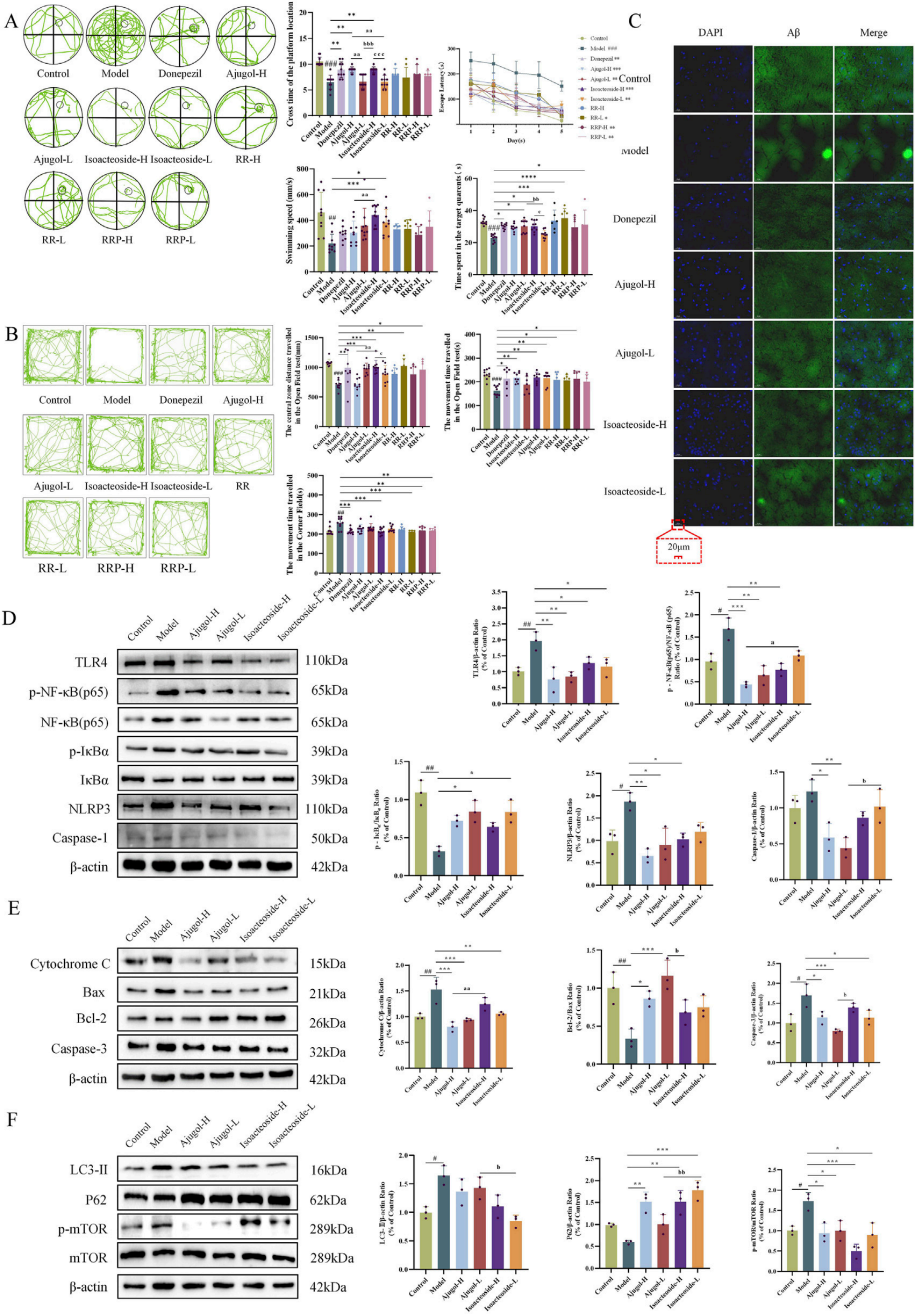
Effects of ajugol and isoacteoside on the physiological indexes, the TLR/NF-κB/NLRP3 signaling pathway, and the apoptosis- and autophagy-related pathways in ICR mice. **(A)** Morris water maze test in ICR mice, including a representative plot of the swimming path during the spatial exploration trial during five consecutive days, the number of times the mice crossed the platform location, the time spent in the target quadrant, the swimming speed, and the escape latency during training. **(B)** Open-field test in ICR mice, including a representative diagram of the open-field trial, the total distance traveled, the distance traveled in the central zone, and the time spent in the corner field; *n* = 10 per group. **(C)** Representative thioflavin-S staining of hippocampal tissue of ICR mice (×40). *n* = 3 per group. **(D–F)** Ajugol and isoacteoside significantly impacted the TLR/NF-κB/NLRP3 signaling pathway, along with the protein expression levels of cytochrome C, BAX, BCL-2, caspase-3, LC3-Ⅱ, P62, and p-mTOR/mTOR in the hippocampal tissues of ICR mice. D-galactose (60 mg/kg/day)/AlCl_3_ (100 mg/kg/day); ajugol-high/low (H/L) (100/50 mg/kg/day); isoacteoside-H/L (5/2.5 mg/kg/day). The data are expressed as the means ± SEM. *n* = 10 per group. ^#^ *p* < 0.05, ^##^ *p* < 0.01, and ^###^ *p* < 0.001 vs. the control group; ^*^ *p* < 0.05, ^**^ *p* < 0.01, and ^***^ *p* < 0.001 vs. the model group; ^a^ *p* < 0.05, ^aa^ *p* < 0.01, and ^aaa^ *p* < 0.001 vs. the ajugol-H group; ^b^ *p* < 0.05, ^bb^ *p* < 0.01, and ^bbb^ *p* < 0.001 vs. the ajugol-L group.

In the Morris water maze test, compared with the model group, mice in each treatment group exhibited a markedly shortened escape latency, along with significant increases in platform crossings, time spent in the target quadrant, and swimming speed (Figure 5A). In the open-field test, compared to the model group, mice in all the treatment groups showed a marked decrease in the time spent in the corner field, along with a significant increase in the distance traveled and time spent in the central zone (Figure 5B).

**FIGURE 6:**
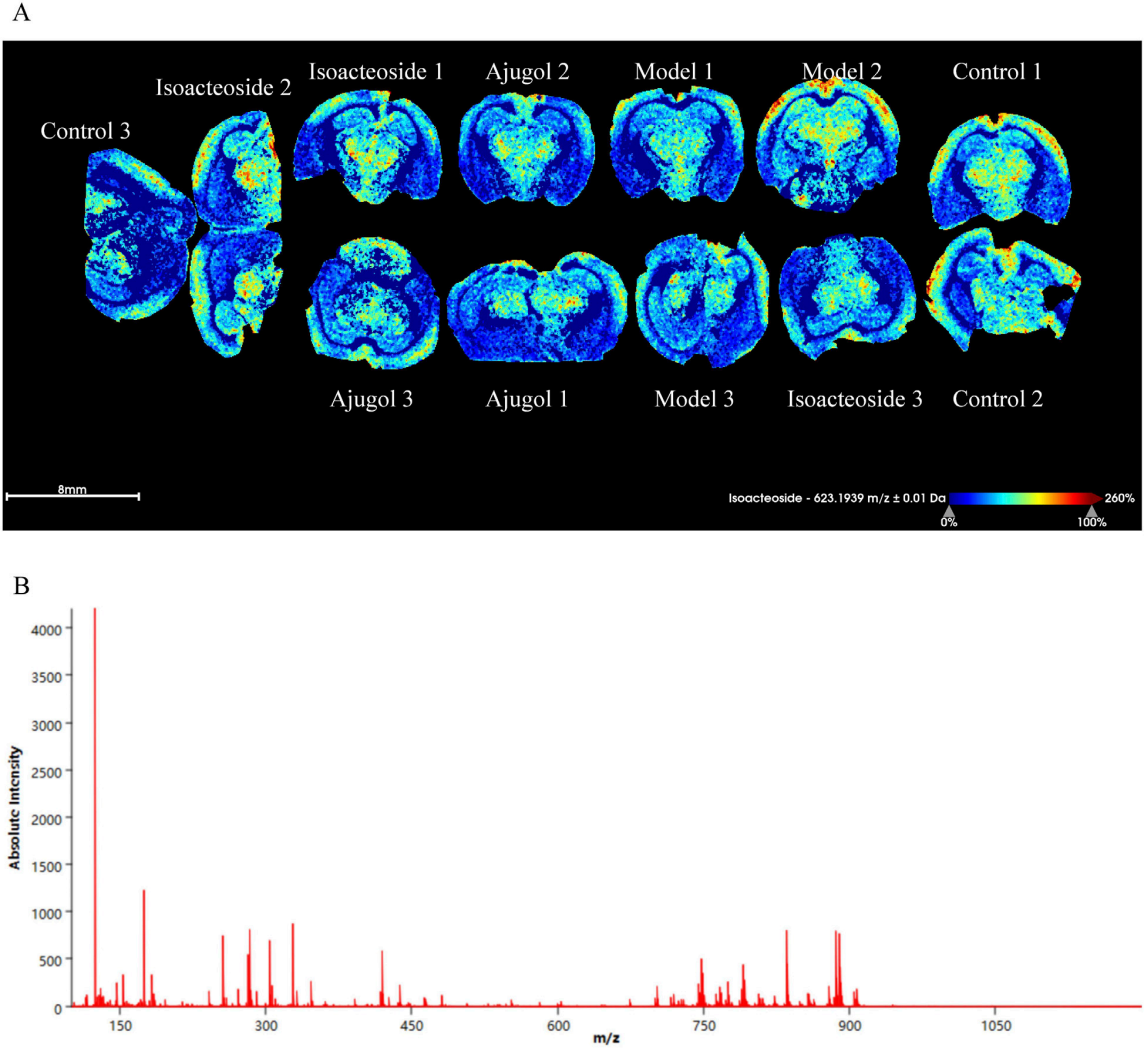
MALDI-MSI of ajugol and isoacteoside distribution in hippocampal tissue of ICR mice (*n* = 3 per group). **(A)** False-color imaging of brain slices labeled with isoacteoside and ajugol. **(B)** Mass spectrometry graph showing absolute intensity vs. m/z ratio.

Aβ plaque deposition is a hallmark feature of AD. Thioflavin-S staining of the hippocampal tissue for Aβ plaques revealed that compared with the control group, the plaque area (stained bright green) in the model group was increased, indicating a marked increase in Aβ protein expression. In contrast, each treatment group showed a significant reduction in plaque area compared with that in the model group (Figure 5C). MALDI–MSI showed that ajugol was not present in the hippocampal tissue, while isoacteoside was detected at −623.1939 *m*/*z* (Figure 6). These observations suggest that ajugol may participate in *in vivo* metabolism and decompose into other substances—a phenomenon worthy of further investigation.

### 3.7 Differences in the expression levels of proteins involved in neuroinflammation, apoptosis, and autophagy-related pathways in the ICR mouse model induced by ajugol and isoacteoside

In the ICR mouse model, the levels of the neuroinflammation pathway-related proteins TLR4, p-NF-κB (p65)/NF-κB (p65), NLRP3, and caspase-1 were significantly decreased in the treatment groups, whereas those of p-IκBɑ/IκBɑ were significantly increased. Notably, the levels of p-NF-κB (p65)/NF-κB (p65) were markedly higher in the isoacteoside-L group than those in the ajugol-H group (*p* < 0.05). Similarly, the isoacteoside-L group exhibited a markedly higher level of caspase-1 protein than that in the ajugol-L group (*p* < 0.05). These results revealed that ajugol in RR significantly influences protein expression in the TLR/NF-κB/NLRP3 pathway in ICR mice, likely accounting for its anti-AD effect (Figure 5D).

In ICR model mice, the expression levels of the apoptosis pathway-related proteins varied among the different treatment groups. Specifically, the protein contents of cytochrome C and caspase-3 were significantly decreased, whereas those of BCL-2 and BAX were significantly increased. Cytochrome C levels were markedly higher in the isoacteoside-H group than in the ajugol-H group (*p* < 0.01). In contrast, compared with the ajugol-L group, the contents of caspase-3 were markedly higher (*p* < 0.05) in the isoacteoside-H group, whereas those of BCL-2/BAX were significantly decreased (*p* < 0.05) (Figure 5E). The above results indicated that ajugol in RR has a more marked effect on the levels of proteins involved in the apoptosis pathway (downregulating the cytochrome C/caspase-3 ratio and upregulating the BCL-2/BAX protein ratio), which may underlie its anti-AD effects.

In the ICR mouse model, the levels of the autophagy pathway-related protein mTOR, along with its phosphorylated form, p-mTOR, were significantly decreased across all the treatment groups. The levels of LC3-Ⅱ were also decreased, although not significantly. In contrast, the expression of P62 was significantly increased. Notably, compared to the ajugol-L group, the expression of P62 in the isoacteoside-L group was greatly upregulated (*p* < 0.01), whereas that of LC3-Ⅱ showed the opposite trend (*p* < 0.05) (Figure 5F). These findings suggested that isoacteoside in RRP had a substantial effect on the expression of autophagy pathway-related proteins in ICR mice (upregulating the P62 protein ratio and downregulating LC3-Ⅱ and the p-mTOR/mTOR ratio), which may explain its anti-AD effects.

### 3.8 Ajugol and isoacteoside both attenuated Aβ toxicity in the BV2 microglial cell inflammation model

**FIGURE 7:**
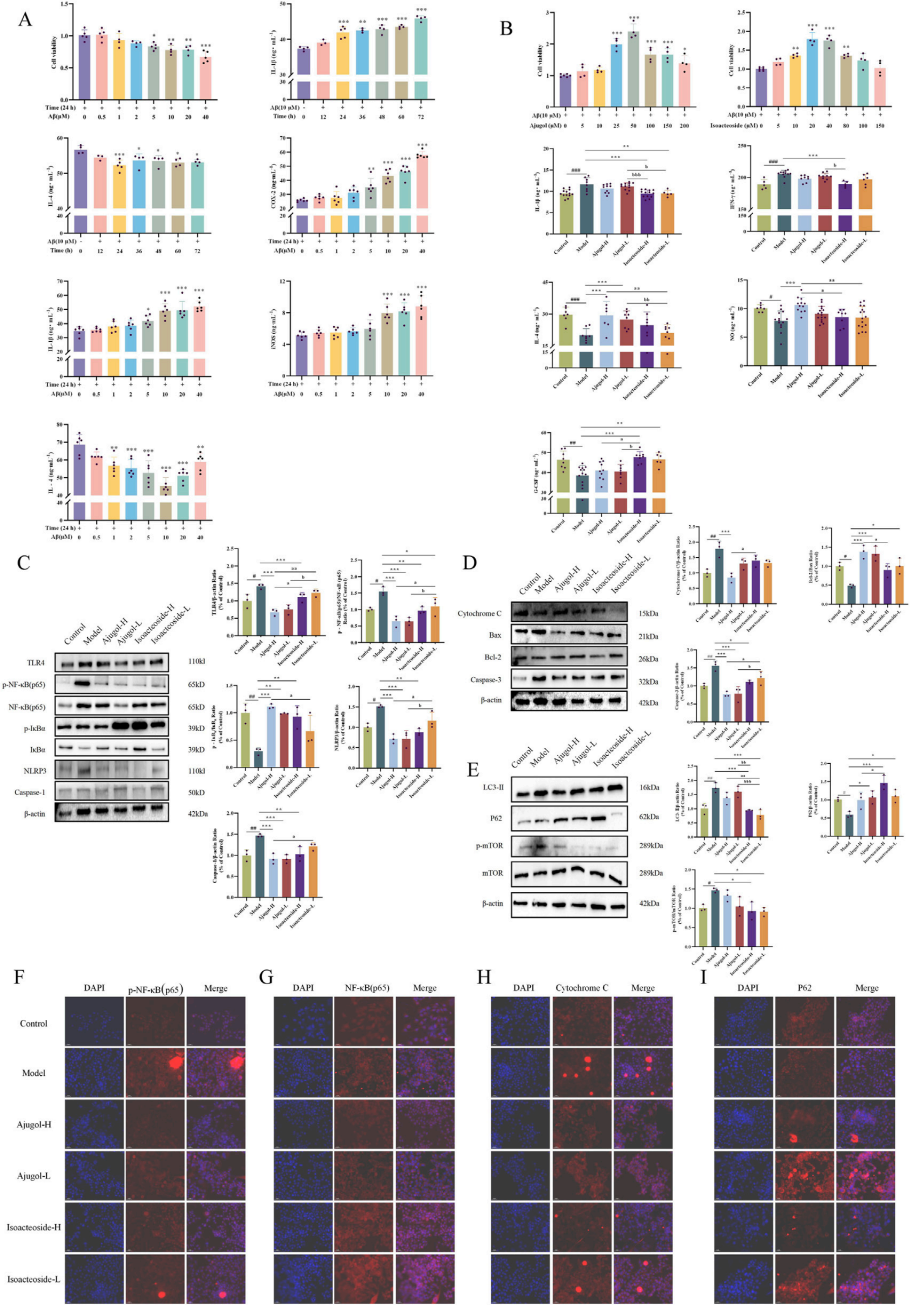
Effects of ajugol and isoacteoside on BV2 cell viability and regulation of the TLR/NF-κB/NLRP3, apoptotic, and autophagic pathways. **(A, B)** Effects of active compounds of RR and RRP on BV2 cell viability. **(C–E)** Effects of ajugol and isoacteoside on the TLR/NF-κB/NLRP3 signaling pathway and the protein levels of cytochrome C, BAX, BCL-2, caspase-3, LC3-Ⅱ, P62, and p-mTOR/mTOR in BV2 cells. **(F–I)** Representative immunofluorescence images of p-NF-κB (p65), NF-κB (p65), cytochrome C, and P62 (red). DAPI nuclear staining is shown in blue. Aβ (10 μM); ajugol-high/low (H/L) (50/25 μM); isoacteoside-H/L (40/20 μM). The data are expressed as the means ± SEM. *n* = 3 per group. ^#^ *p* < 0.05, ^##^ *p* < 0.01, and ^###^ *p* < 0.001 vs. the control group; ^*^ *p* < 0.05, ^**^ *p* < 0.01, and ^***^ *p* < 0.001 vs. the model group; ^a^ *p* < 0.05, ^aa^ *p* < 0.01, and ^aaa^ *p* < 0.001 vs. the ajugol-H group; ^b^ *p* < 0.05, ^bb^ *p* < 0.01, and ^bbb^ *p* < 0.001 vs. the ajugol-L group.

At concentrations ranging from 0.5 to 2 μM, Aβ had no apparent effect on the viability of BV2 cells after 24 h of treatment. However, cell viability significantly decreased at Aβ concentrations exceeding 5 μM. Accordingly, a concentration of 10 μM of Aβ and a duration of 24 h were selected for the subsequent modeling (Figure 7A).

Ajugol concentrations of 5 μM–10 μM did not affect BV2 cell viability. However, cell viability significantly declined when the ajugol concentration exceeded 25 μM. Therefore, in subsequent experiments, 50 μM as a high concentration and 25 μM as a low concentration were used for ajugol treatment in BV2 cells. Similarly, in the subsequent experiments, 40 μM was used as a high concentration for isoacteoside treatment, while 20 μM was applied as a low concentration for the treatment of BV2 cells (Figure 7B).

Compared with the model group, the levels of IL-1β were markedly decreased in all the treatment groups, whereas those of IL-4, NO, and G-CSF were notably elevated. The NO levels were markedly lower in the isoacteoside-H group than in the isoacteoside-L (*p* < 0.01) and ajugol-H (*p* < 0.05) groups, whereas the G-CSF levels increased markedly compared to those in the isoacteoside-H group (*p* < 0.05). Compared with those in the ajugol-L group, IL-1β levels exhibited a marked reduction in the isoacteoside-H group (*p* < 0.001) and isoacteoside-L group (*p* < 0.05), whereas IFN-γ was markedly reduced compared with that in the isoacteoside-H group (*p* < 0.05), IL-4 was notably reduced compared with that in the isoacteoside-L group (*p* < 0.01), and G-CSF contents were notably higher than those in the isoacteoside-H group (*p* < 0.05) (Figure 7B).

The above results suggested that both ajugol in RR and isoacteoside in RRP can modulate the contents of IL-1β, IFN-γ, IL-4, NO, and G-CSF in Aβ-exposed mouse BV2 microglial cells, thereby exerting anti-AD effects. Specifically, ajugol in RR mediates anti-inflammatory effects, whereas isoacteoside in RRP enhances the expression of neurotrophic factors, which improve the AD symptoms.

### 3.9 Comparative analysis of the effects of ajugol and isoacteoside on the expression of proteins involved in neuroinflammation, apoptosis, and autophagy in BV2 microglial inflammation models

Consistent with the setup in Section 3.7, the expression of proteins associated with neuroinflammatory, apoptotic, and autophagic pathways in the BV2 microglial cell model of inflammation was monitored using Western blotting. The results indicated that ajugol in RR had a more notable impact on the concentrations of proteins related to neuroinflammation in the BV2 cell model (Figure 7C). In addition, ajugol in RR had a marked effect on the expression of proteins in the apoptosis pathway in these cells (increasing the BCL-2/BAX ratio and reducing the cytochrome C/caspase-3 ratio) (Figure 7D). In RRP, isoacteoside had a more notable effect on the content of autophagy pathway-related proteins in the BV2 cell inflammation model (upregulating the P62 ratio and downregulating the ratio of LC3-Ⅱ and p-mTOR/mTOR) (Figure 7E), which was consistent with the *in vivo* validation results.

To verify the mechanism of action, IF was used to monitor the expression of each protein. Consistent with the above results, we found that ajugol in RR has a more significant regulatory effect on the contents of p-NF-κB (p65)/NF-κB (p65) (Figures 7F, G) and cytochrome C. Meanwhile, isoacteoside in RRP had a marked regulatory effect on P62 contents (Figure 7I).

**FIGURE 8:**
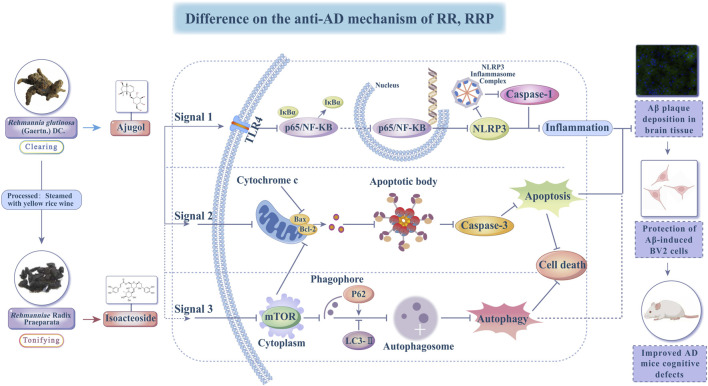
Diagram showing the mechanisms behind the effects of ajugol and isoacteoside on AD.

In summary, ajugol, the active compound in RR, and isoacteoside, the active compound in RRP, showed different effects on the expression of proteins connected with the neuroinflammatory, apoptotic, and autophagic pathways. Specifically, ajugol in RR has a more significant regulatory effect on the concentration of proteins in the TLR/NF-κB/NLRP3 and BCL-2/BAX/cytochrome C/caspase-3 pathways, whereas isoacteoside in RRP exerts a more significant regulatory effect on the expression of proteins in the LC3-Ⅱ/P62/p-mTOR/mTOR pathway (Figure 8).

## 4 Discussion

The pathological hallmarks of AD include extensive synaptic and neuronal loss, proliferation of inflammatory glial cells, and the formation of neurofibrillary tangles composed of Aβ and hyperphosphorylated tau. As a comprehensive therapeutic approach, complementary medicine has gained significant attention in AD management. The clinical application of TCM often requires processing, which can markedly alter the chemical composition and pharmacological efficacy of herbal preparations.

Although RR and RRP, both belonging to the food–medicine homologous resources, have long been used in TCM for AD intervention, differences in their bioactive compounds and the underlying mechanisms remain poorly understood. Recent studies suggest that their primary active constituents—such as catalpol, verbascoside, 5-HMF, and polysaccharides—exert neuroprotective effects through mechanisms including anti-oxidation, neuroprotection, anti-inflammation, immune modulation, and anti-apoptosis (Ya et al., 2017; Gao et al., 2018; Chen H. et al., 2022). Furthermore, miRNA-138-5p, which is highly expressed in the nervous system, plays a critical role in memory regulation and promotes axonal regeneration.

Catalpol can promote the secretion of miR-138-5p by neural stem cells, thereby effectively alleviating the symptoms of AD. Metabolomics allows the precise analysis of the functional levels and changes in metabolites in biological systems, providing a microscopic understanding of the complex mechanisms and scientific essence of TCM (Yang X. et al., 2024). In this research, UPLC–QE-MS/MS technology was used to authenticate chemical and bioactive compounds in the serum and brain tissue and the metabolites of RR and RRP. Consistent with previous studies, RR and RRP extracts were found to include the iridoid glycosides catalpol and aucuboside, terpenoids, flavonoids, lignans, and various polysaccharides (Chen H. et al., 2022; Bian et al., 2023; Jia et al., 2023). This finding confirms that RR and its active compound ajugol and RRP and its active compound isoacteoside may improve the pathology associated with Aβ plaque accumulation in the hippocampal tissue of AD mice and improve their spatial cognition.

Based on the fundamental principles of TCM, RR is known to clear heat, cool blood, and facilitate fluid production. Meanwhile, RRP has a significant effect on nourishing *Yin* and essence, replenishing blood, and tonifying pulp. Herbal medicines are commonly used for AD treatment. In the present work, we conducted a systematic network pharmacology investigation. The network pharmacology results suggested that the critical targets of RR were primarily associated with biological processes such as apoptosis and chemical carcinogenesis–reactive oxygen species signaling pathways. In contrast, the targets of RRP were noted to be related to the modulation of the TLR/NF-κB/NLRP3 signaling pathway and responses to nutritional metabolism and cell growth. This reveals that RR processing enhances the effects of nourishing *Yin* and replenishing the blood. Paudel et al. (2020) and Xie et al. (2025) reported that the TLR/NF-κB/NLRP3 signaling pathway mediates inflammation-related signal transduction and inflammatory factor release, leading to inflammatory responses that cause neuronal damage and neurodegenerative changes. Phagocytosis of Aβ induces the secretion of IL-1β through the NLRP3-dependent activation of caspase-1, highlighting the involvement of the NLRP3 inflammasome in the development of AD (Halle et al., 2008). This implies that the bioactive compounds of RR and RRP exert their ameliorative effects in AD by influencing inflammatory responses through the TLR/NF-κB/NLRP3 signaling pathway. Previous research by our group identified significant differences between RR and RRP in improving autophagy in the T2DM mouse model. Similarly, the results of the present study show that significant differences exist between RR and RRP in improving autophagy in mice with AD (Yan et al., 2023).

Significantly, the interplay between mTOR signaling and autophagy represents a pivotal axis in AD pathogenesis. The hyperactivation of mTOR (mTORC1) suppresses autophagic flux, leading to impaired clearance of neurotoxic Aβ aggregates and hyperphosphorylated tau, which are core pathological hallmarks of AD. Conversely, downregulation of mTOR activity triggers autophagy, promoting the degradation of misfolded proteins and mitigating AD progression (Subramanian et al., 2022; Cordos et al., 2025). Our findings align with this trilateral association: isoacteoside from RRP specifically modulates the LC3-II/P62/p-mTOR/mTOR pathway, indicating that its pro-autophagic effects likely occur *via* mTOR inhibition. This mechanistic synergy, wherein mTOR suppression activates autophagy to clear pathogenic proteins, establishes the molecular basis for isoacteoside’s efficacy in mitigating both the Aβ plaque burden and the cognitive deficits in AD models.

The pathogenesis of AD has long been a major focus and challenge in neuroscience research. A primary pathological hallmark of AD is the extensive extracellular deposition of Aβ, leading to amyloid plaque formation. The accumulation of Aβ can also trigger abnormal activation of BV2 microglial cells (Di Stadio and Angelini, 2019). As resident immune cells in the central nervous system, BV2 cells play a key role in neuroinflammation, an important contributor to AD progression (Cui et al., 2023; Mondal et al., 2024). BV2 cells exhibit two polarization states: M1 and M2. M1-polarized BV2 cells release pro-inflammatory cytokines and chemokines, contributing to neuronal damage, whereas M2-polarized BV2 cells secrete anti-inflammatory cytokines and neurotrophic factors, conferring neuroprotection (Jana et al., 2008).

In this study, Aβ was used to establish an *in vitro* inflammatory model in BV2 cells. Focusing on the TLR/NF-κB/NLRP3 signaling pathway, along with apoptosis and autophagy, we demonstrated that RR and its active component ajugol, along with RRP and its active component, isoacteoside, significantly increased the levels of anti-inflammatory IL-4 in Aβ-treated BV2 cells while markedly reducing those of pro-inflammatory mediators such as IL-1β. Furthermore, the differential regulation of TLR/NF-κB/NLRP3 signaling and the apoptosis/autophagy pathways appears to be a critical mechanism underlying the anti-AD effects of these compounds.

Several important limitations of this study remain unresolved, including the lack of investigation into BV2 microglial M1/M2 polarization and the absence of causal validation for the gut–brain axis interactions. Future research should incorporate single-cell RNA sequencing to elucidate the mechanisms of microglial reprogramming, utilize fecal microbiota transplantation to confirm the contribution of gut microbiota to autophagy enhancement, and explore the development of nano-formulated RR/RRP functional foods to improve blood–brain barrier penetration. Additionally, establishing human neuron-immune co-culture systems and optimizing the pharmacokinetics of active compounds will be essential steps toward accelerating the clinical translation of these food–medicine homology strategies for AD therapy.

Currently, most clinical therapeutic drugs for AD have significant drawbacks, including severe side effects and high costs, and their neuroprotective activity often remains unknown. In contrast, the food and medicine homology approach, characterized by holistic regulation, multi-targeting, and individualized medication (Deng et al., 2022; Wang et al., 2024), presents a viable option for treating AD. It offers efficacy, convenience, cost-effectiveness, and safety, and it significantly improves the clinical symptoms of AD. This approach represents a common strategy for treating chronic metabolic diseases such as AD.

## 5 Conclusion

This study systematically demonstrated that the function of RR, a food and medicine homology, shifts from “clearing” to “tonifying” when processed into RRP. Ajugol (from RR) and isoacteoside (from RRP) were identified as the key bioactive compounds responsible for their anti-AD effects. Ajugol primarily mitigates AD pathology by regulating the TLR/NF-κB/NLRP3 inflammasome pathway and the BCL-2/BAX/cytochrome C/caspase-3 apoptosis pathway, thereby reducing neuroinflammation and neuronal death. Conversely, isoacteoside exerts neuroprotection predominantly *via* the LC3-Ⅱ/P62/p-mTOR/mTOR autophagy pathway, enhancing the clearance of Aβ aggregates. Both compounds significantly attenuated cognitive impairment, reduced Aβ plaque accumulation in AD models, and suppressed microglial cytotoxicity. These findings lay the foundation for the development and application of RRP, thus establishing a basis for developing complementary medicines and functional foods.

## Data Availability

The datasets presented in this study can be found in online repositories. The names of the repository/repositories and accession number(s) can be found in the article/Supplementary Material.
