# Supplementary material for: Systems pharmacology identifies ajugol-mediated NF-κB/caspase-3 inhibition and isoacteoside-driven p62/mTOR-mediated autophagy as key mechanisms of Rehmanniae Radix and its processed form in Alzheimer’s treatment
Source: Front Pharmacol. 2025 Aug 29;16:1644847. doi: 10.3389/fphar.2025.1644847 (PMC12426409; doi:10.3389/fphar.2025.1644847)
Supplement: Supplementary file 1 [file DataSheet1.pdf]

Supporting Information for

Original article

Systems pharmacology identifies ajugol-mediated NF-κB/caspase-3 inhibition and isoacteoside-driven p62/mTOR-mediated autophagy as key mechanisms of Rehmanniae Radix and its processed form in Alzheimer's treatment

Table of Contents

Table S1 Results of chemical composition identification of BPC icon peak of RR

Table S2 Results of chemical composition identification of BPC icon peak of RRP

Table S3 Binding Energy for targets with their drugs

Table S4 The contents of Catalpol, Ajugol, 5-HMF, Isoacteoside from *Rehmannia glutinosa* (Gaertn.) DC. Sample and *Rehmanniae* Radix Praeparata Sample.

Figure S1 HPLC of reference substance, *Rehmannia glutinosa* (Gaertn.) DC. Sample and *Rehmanniae* Radix Praeparata. (A) Catalpol; (B) Ajugol; (C) 5-HMF; (D) Isoacteoside.

Figure S1(A)-1 Base peak chromatogram of the supernatant with RR extracts detected by UHPLC-QE-MS/MS in positive-ion mode.

Figure S1(A)-2 Base peak chromatogram of the supernatant with RR extracts detected by UHPLC-QE-MS/MS in negative-ion mode.

Figure S1(A)-3 Base peak chromatogram of the supernatant with RRP extracts detected by UHPLC-QE-MS/MS in positive-ion mode.

Figure S1(A)-4 Base peak chromatogram of the supernatant with RRP extracts detected by UHPLC-QE-MS/MS in negative-ion mode.

Figure S1(A)-5 Identification of prototypic compounds of RR and RRP absorbed into blood/brain of APP/PS1 mice.

Figure S1(B)-1 Full scan MS spectrum of RR component (Ajugol) at -393.1404 m/z from LC-MS dataset of the blood sample.

Figure S1(B)-2 Full scan MS spectrum of RRP component (Ajugol) at -393.1404 m/z from LC-MS dataset of the blood sample.

Figure S1(B)-3 Full scan MS spectrum of RRP component (Isoacteoside) at -623.1939 m/z from LC-MS dataset of the blood sample.

Figure S1(B)-4 Full scan MS spectrum of RR component (Ajugol) at -393.1404 m/z from LC-MS dataset of the brain sample.

Figure S1(B)-5 Full scan MS spectrum of RRP component (Ajugol) at -393.1404 m/z from LC-MS dataset of the brain sample.

Figure S1(B)-6 Full scan MS spectrum of RRP component (Isoacteoside) at

-623.1939 m/z from LC-MS dataset of the brain sample.

Figure S2 The core targe (Top20), GO and KEGG enrichment analysis diagram of RR and RRP in the treatment of AD

Figure S3 The results of RR, RRP active components (Verbascoside) respectively with seven kinds of proteins (TLR4, NFKBIA and NLRP3, BCL2, CASP3, P62, MTOR) molecular docking.

Figure S4 Effects of RR and RRP on gut microbiota in APP/PS1 mice.

Supplementary information 2 The original images of the western blotting of the expression level in brains of each group for three independent repeats.

Supplementary information 3 The original images of the western blotting of the expression level in BV2 cells of each group for three independent repeats.

Table S1 Results of chemical composition identification of BPC icon peak of RR

No.	m/z	RT/min	ppm	Compound name	Score	Into Blood or None	Into Tissue or None
1	130.0500	1.10	0.4	Glutamic acid	0.9999	None	None
2	385.1102	1.18	6.6	Sesamoside	0.9987	Into Blood	None
3	268.1039	1.20	0.6	Adenosine	0.9998	None	None
4	132.1018	1.33	1	L-Norleucine	0.9942	None	None
5	283.1398	1.58	NA	NA	NA	Into Blood	None
6	166.0858	2.04	17.2	Phenylalanine	0.9998	None	None
7	168.0655	2.35	0	[2-(Acetylamino)phenoxy]acetic acid	0.9867	None	None
8	213.1234	2.80	19.2	Epiligulyl oxide	0.8273	Into Blood	Into Tissue
9	126.0549	2.85	0.1	4-Amino-1,2-benzenediol	0.9584	None	None
10	129.0545	3.19	0.2	Dimethyl glutarate	0.8484	None	None
11	148.0758	3.56	0.9	2-Methylindol-6-ol	0.9987	None	None
12	217.0971	3.91	16.8	L-1,2,3,4-Tetrahydro-beta-carboline-3-carboxylic acid	0.8245	Into Blood	None
13	231.1118	4.10	0.6	Tetrahydroharman-3-carboxylic acid	0.949	Into Blood	None
14	240.1017	4.58	16.3	2,4-Dinitro-N-butylaniline	0.9746	None	None
15	163.0385	5.03	1.9	Butein	0.9851	None	None
16	128.0342	1.21	8.5	Pidolic acid	0.9998	None	None
17	117.0182	1.36	9.4	Succinic acid	0.9988	Into Blood	None
18	393.1404	2.79	1.1	Ajugol	0.8846	Into Blood	Into Tissue
19	375.1297	3.19	0.4	Mussaenosidic acid	0.9371	Into Blood	None
20	785.2514	4.30	1.2	Purpureaside C	0.9962	None	None
21	799.2671	4.66	NA	NA	NA	None	None
22	435.2234	5.00	3	3-Hydroxy-3-methyl-5-oxo-5-(((2R,3S,4S,5R,6R)-3,4,5-trihydroxy-6-(octan-3-yloxy)tetrahyd	0.8257	Into Blood	None

				ro-2H-pyran-2-yl)methoxy)pentanoic acid			
23	623.1981	5.17	0.4	Verbascoside	0.9964	None	None
24	183.1020	5.20	4.2	Hexylsuccinic acid	0.9874	Into Blood	Into Tissue
25	623.1984	5.45	0.4	ZINC67902872	0.9754	None	None
26	183.1018	7.74	4.2	cis-Pinonic acid	0.9929	Into Blood	Into Tissue

Table S2 Results of chemical composition identification of BPC icon peak of RRP

No.	m/z	RT/min	ppm	Compound name	Score	Into Blood or None	Into Tissue or None
1	305.1339	1.04	14.7	8-Hydroxy-3-(7-hydroxyheptyl)-6-oxo-3,4-dihydroisochromene-7-carboxylic acid	0.9208	None	None
2	237.0868	1.17	18.5	Apomorphine	0.8697	None	None
3	365.1050	1.27	0.7	Melibiose	0.9972	Into Blood	None
4	145.0494	1.86	0.8	(3E)-3-Hexenedioic acid	0.9494	None	None
5	130.0498	1.96	0.7	Glutamic acid	0.9998	Into Blood	Into Tissue
6	127.0389	2.51	NA	NA	NA	None	None
7	129.0546	3.28	0.2	Monomethyl glutarate	0.8772	None	None
8	217.0966	3.95	17.1	L-1,2,3,4-Tetrahydro-beta-carboline-3-carboxylic acid	0.8246	None	None
9	177.0539	4.57	1.1	Homoeriodictyol	0.9833	None	None
10	163.0385	5.07	2.5	Butein	0.987	None	None
11	163.0386	5.34	1.9	Rosmarinic acid	0.9836	None	None
12	147.0439	6.34	2.2	MDPPP	0.9942	None	None
13	373.1140	2.34	0.1	Ketologanic acid	0.7527	Into Blood	None
14	141.0182	2.54	6.1	5-(Hydroxymethyl)-2-furoic acid	0.9887	Into Blood	None
15	393.1403	2.87	0.8	Ajugol	0.8899	Into Blood	Into Tissue
16	269.0672	2.99	0.4	Phenol glucuronide	0.8706	None	None

17	375.1296	3.23	0.2	Mussaenosidic acid	0.9376	Into Blood	None
18	192.0660	3.53	3	4-(Acetylmethylamino) benzoic acid	0.9784	None	Into Tissue
19	175.0601	3.92	4	alpha-Isopropylmalate	0.9973	Into Blood	Into Tissue
20	129.0546	4.02	7.9	4-Methyl-2-oxovaleric acid	0.9977	None	None
21	435.1508	4.17	0.7	Loganin	0.9304	Into Blood	None
22	785.2514	4.34	1.4	Purpureaside C	0.9987	None	None
23	210.0767	4.44	0.5	3-Methoxytyrosine	0.9706	Into Blood	None
24	357.1192	4.63	0.4	Juniperoside III	0.789	Into Blood	None
25	435.2235	5.05	0.5	3-Hydroxy-3-methyl-5-oxo-5-(((2R,3S,4S,5R,6R)-3,4,5-trihydroxy-6-(octan-3-yloxy)tetrahydro-2H-pyran-2-yl)methoxy)pentanoic acid	0.7251	Into Blood	Into Tissue
26	623.1982	5.21	0.6	Isoacteoside	0.9933	Into Blood	Into Tissue
27	183.1020	5.27	3.9	cis-Pinonic acid	0.9918	Into Blood	None
28	425.1948	5.36	19.5	3-(N-Maleimidopropionyl)biocytin	0.9528	Into Blood	None
29	623.1982	5.49	0.2	ZINC67902872	0.9776	Into Blood	Into Tissue
30	637.2140	6.23	0.2	6-O-.alpha.-L-(3-O-trans-Cinnamoyl)rhamnopyranosylcatalpol	0.7866	None	None
31	183.1019	7.83	4	(9E,11Z)-8-Hydroxyoctadeca-9,11-dienoic acid	0.9862	Into Blood	None

Table S3 Binding Energy for targets with their drugs

Source	Drug	Target	Binding Energy（kcal/mol）
RR	Ajugol	TLR4	-7.261
		NFKBIA	-7.799
		NLRP3	-6.648
		BCL2	-5.893
		CASP3	-7.421

		P62	-6.179
		MTOR	-6.834
RRP	Verbascoside	TLR4	-6.423
		NFKBIA	-7.344
		NLRP3	-8.614
		BCL2	-6.537
		CASP3	-7.829
		P62	-6.365
		MTOR	-6.721
RRP	Isoacteoside	TLR4	-9.668
		NFKBIA	-9.031
		NLRP3	-9.668
		BCL2	-6.392
		CASP3	-8.868
		P62	-8.110
		MTOR	-8.973

Table S4. The contents of Catalpol, Ajugol, 5-HMF, Isoacteoside from *Rehmannia glutinosa* (Gaertn.) DC. Sample and *Rehmanniae* Radix Praeparata Sample.

Peak no.	RT (min)	Compound	Formula	Content (mg/g)
1	8.963	Catalpol	$C_7H_8O_2$	0.506
2	3.860	Ajugol	$C_{14}H_{20}O_7$	13.144
3	4.957	5-HMF	$C_8H_{10}O_2$	0.898
4	8.457	Isoacteoside	$C_{27}H_{30}O_{16}$	1.166

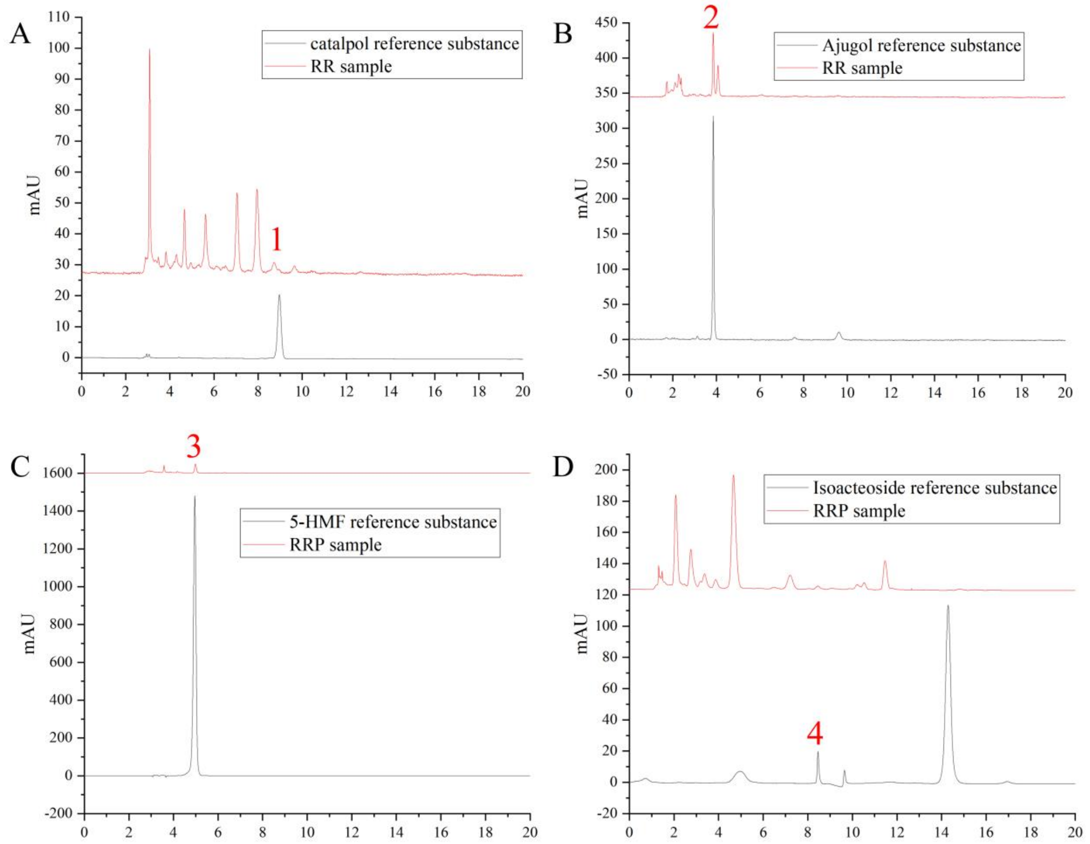

Figure S1. HPLC of reference substance, *Rehmannia glutinosa* (Gaertn.) DC. Sample and *Rehmanniae* Radix Praeparata Sample. (A) Catalpol; (B) Ajugol; (C) 5-HMF; (D) Isoacteoside.

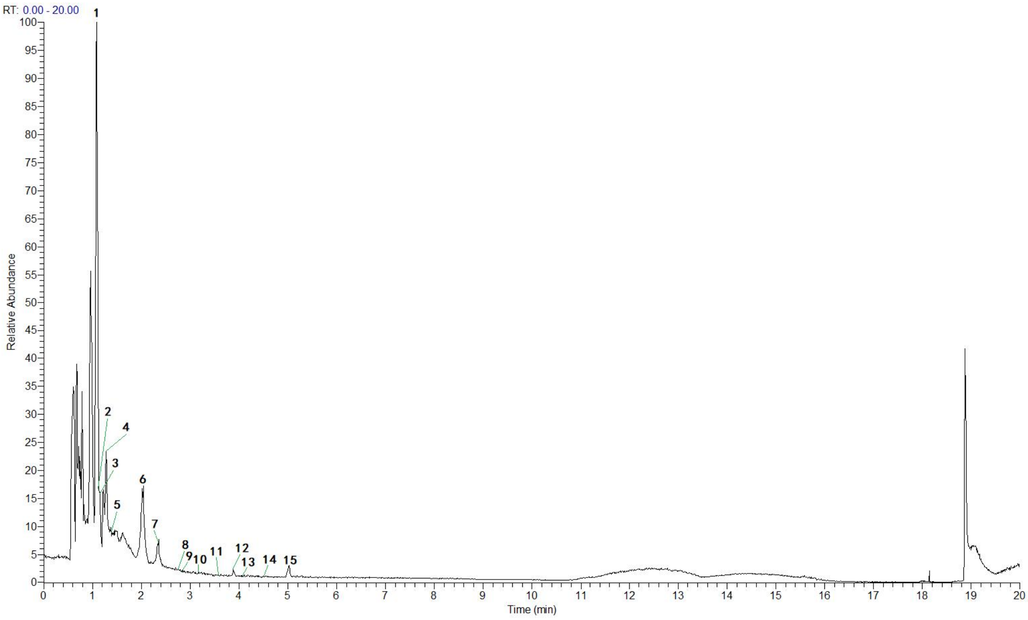

Figure S1(A)-1 Base peak chromatogram of the supernatant with RR extracts

detected by UHPLC-QE-MS/MS in positive-ion mode.

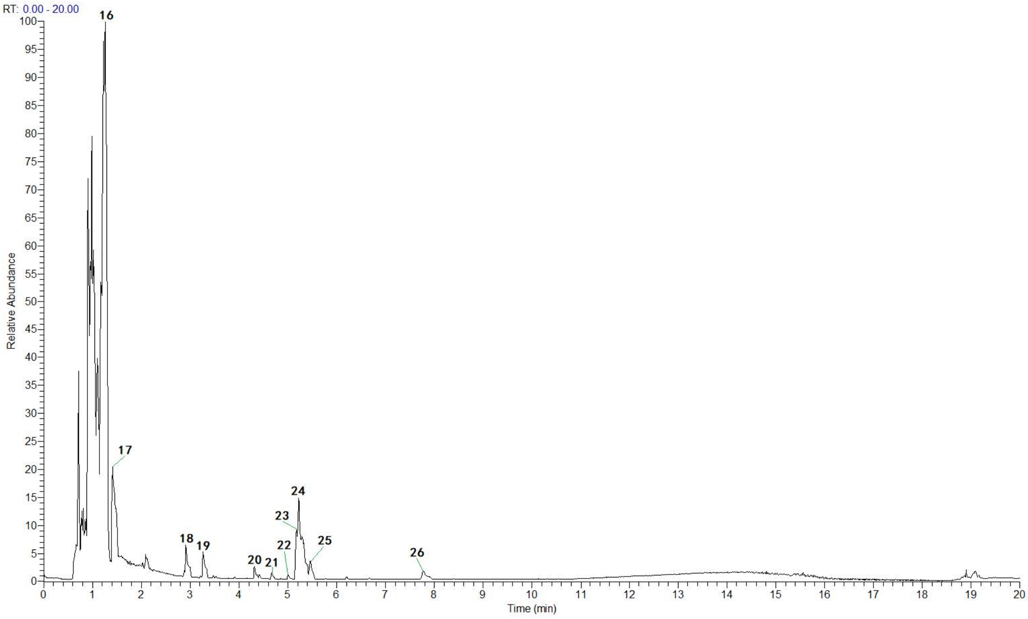

Figure S1(A)-2 Base peak chromatogram of the supernatant with RR extracts detected by UHPLC-QE-MS/MS in negative-ion mode.

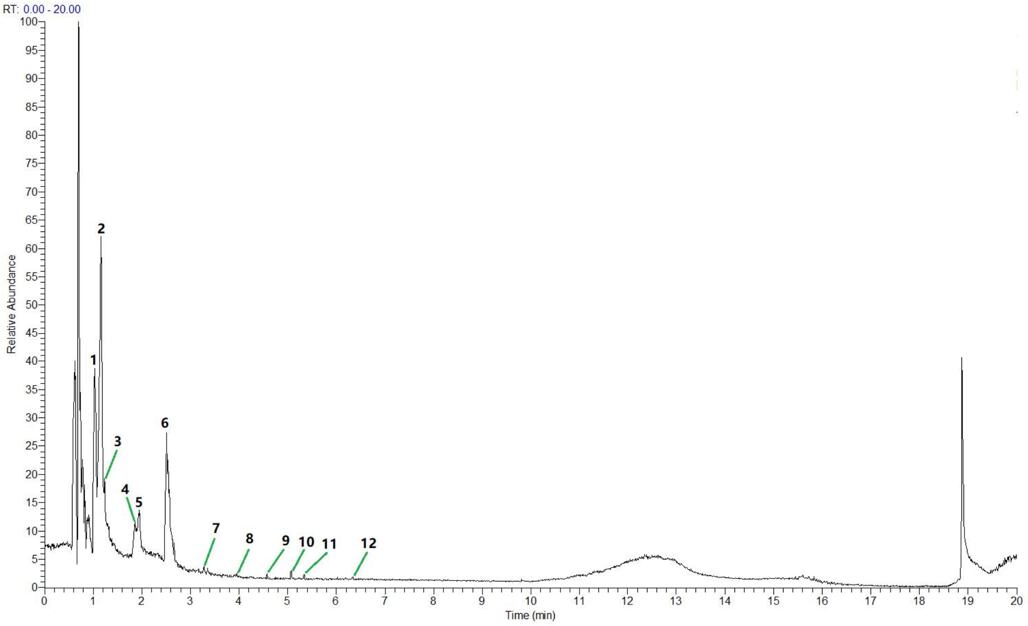

Figure S1(A)-3 Base peak chromatogram of the supernatant with RRP extracts detected by UHPLC-QE-MS/MS in positive-ion mode.

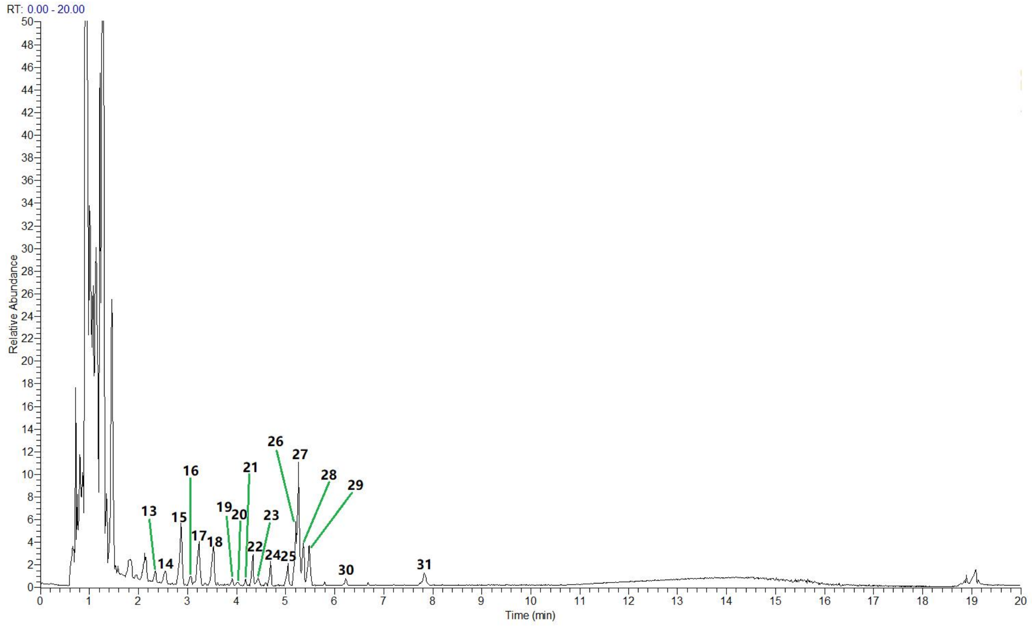

Figure S1(A)-4 Base peak chromatogram of the supernatant with RRP extracts detected by UHPLC-QE-MS/MS in negative-ion mode.

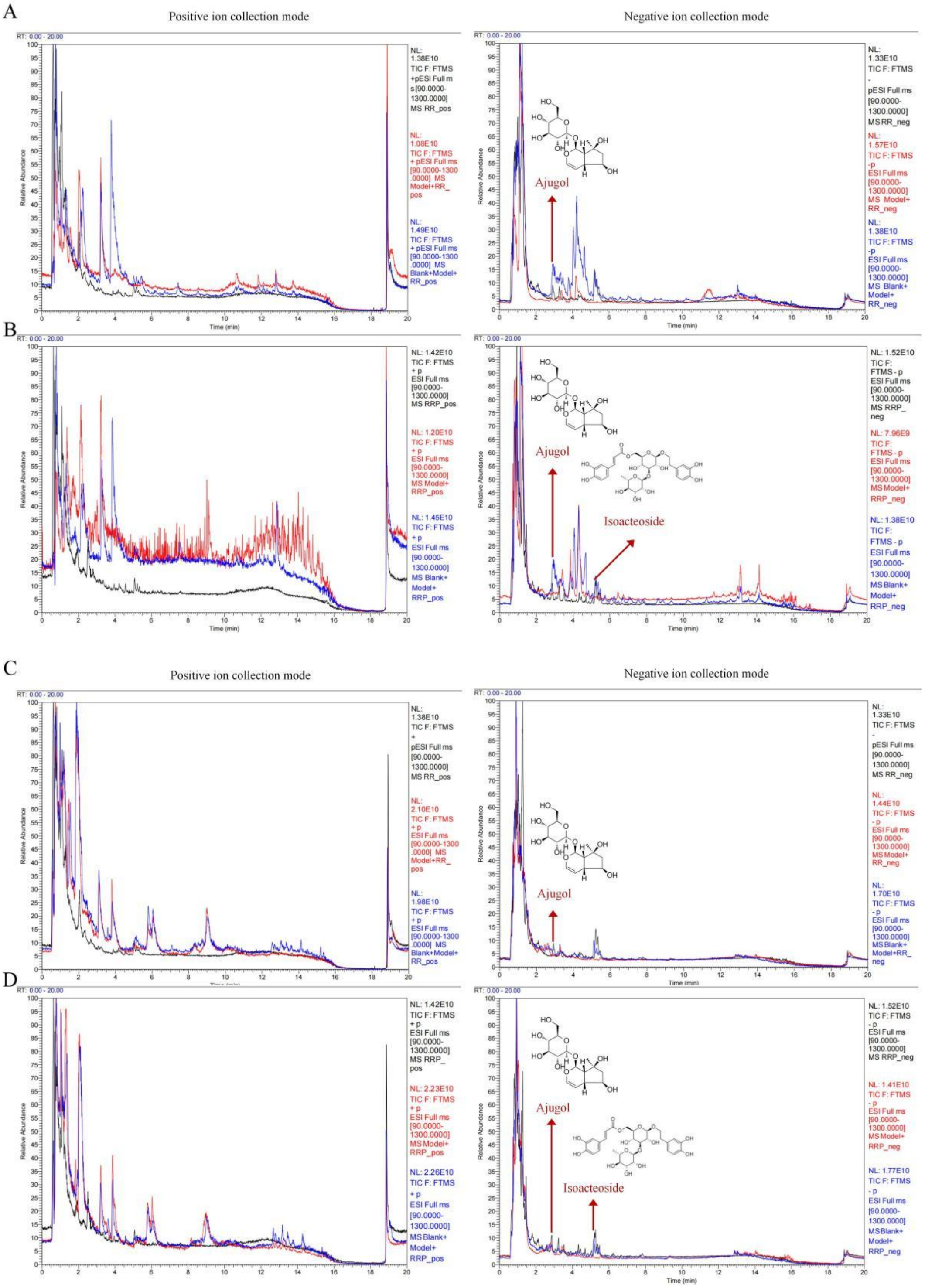

Figure S1(A)-5 Identification of prototypic compounds of RR and RRP absorbed into blood/brain of APP/PS1 mice. **A** Total ion chromatograms of prototype compounds of RR absorbed into the blood detected by UPLC-QE-MS/MS in positive-ion and negative-ion mode. **B** Total ion chromatograms of prototype compounds of RRP absorbed into the blood detected by UPLC-QE-MS/MS in positive-ion and

negative-ion mode. **C** Total ion chromatograms of prototype compounds of RR absorbed into the brain detected by UPLC-QE-MS/MS in positive-ion and negative-ion mode. **D** Total ion chromatograms of prototype compounds of RRP absorbed into the brain detected by UPLC-QE-MS/MS in positive-ion and negative-ion mode. The experiment data were expressed as means±SEM. *n* =3 in each group.

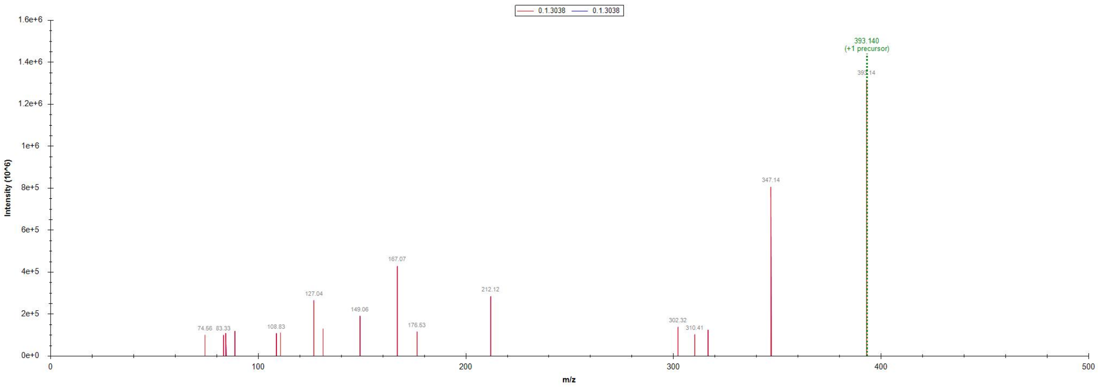

Figure S1(B)-1 Full scan MS spectrum of RR component (Ajugol) at -393.1404 m/z from LC-MS dataset of the blood sample.

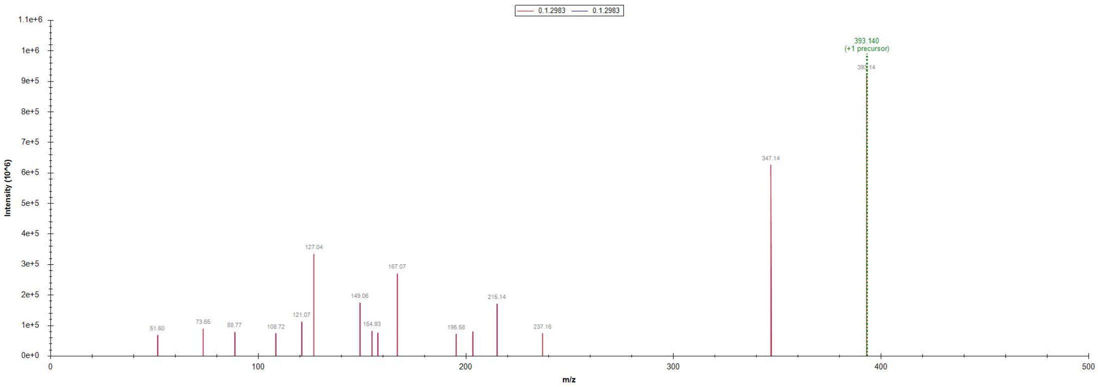

Figure S1(B)-2 Full scan MS spectrum of RRP component (Ajugol) at -393.1404 m/z from LC-MS dataset of the blood sample.

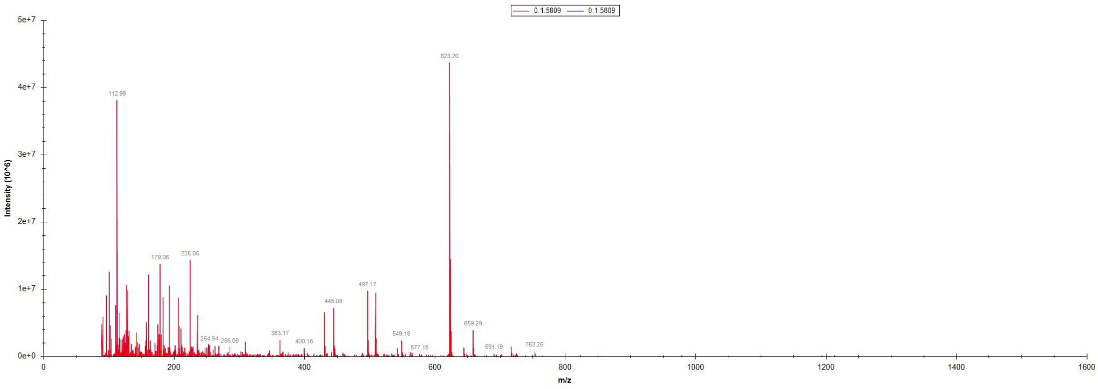

Figure S1(B)-3 Full scan MS spectrum of RRP component (Isoacteoside) at -623.1939 m/z from LC-MS dataset of the blood sample.

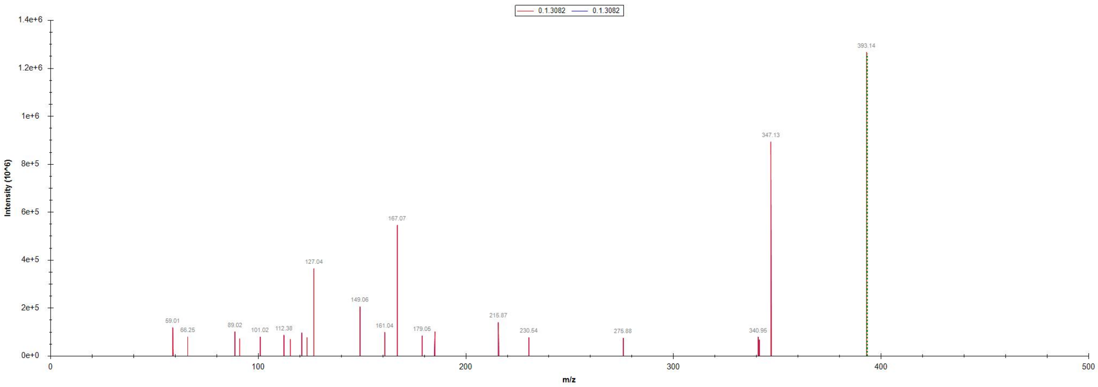

Figure S1(B)-4 Full scan MS spectrum of RR component (Ajugol) at -393.1404 m/z from LC-MS dataset of the brain sample.

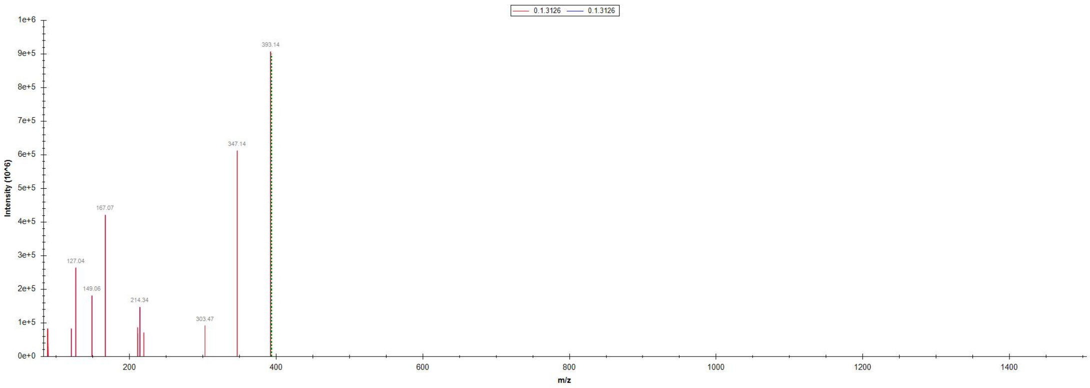

Figure S1(B)-5 Full scan MS spectrum of RRP component (Ajugol) at -393.1404 m/z from LC-MS dataset of the brain sample.

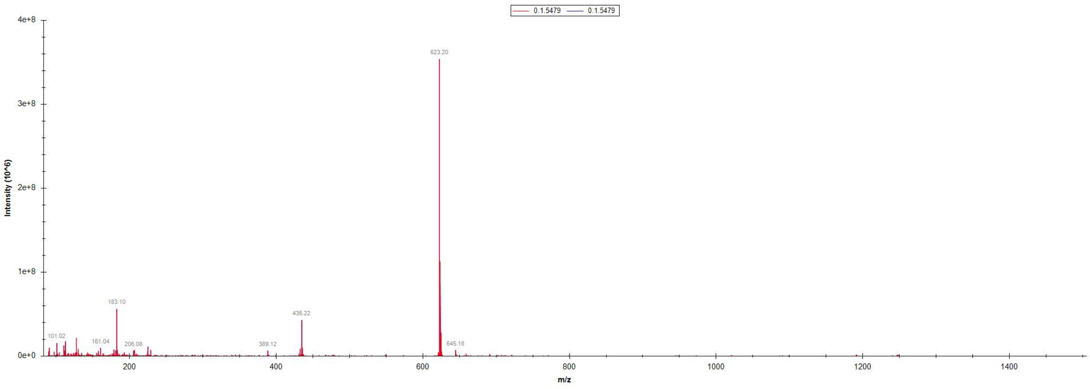

Figure S1(B)-6 Full scan MS spectrum of RRP component (Isoacteoside) at -623.1939 m/z from LC-MS dataset of the brain sample.

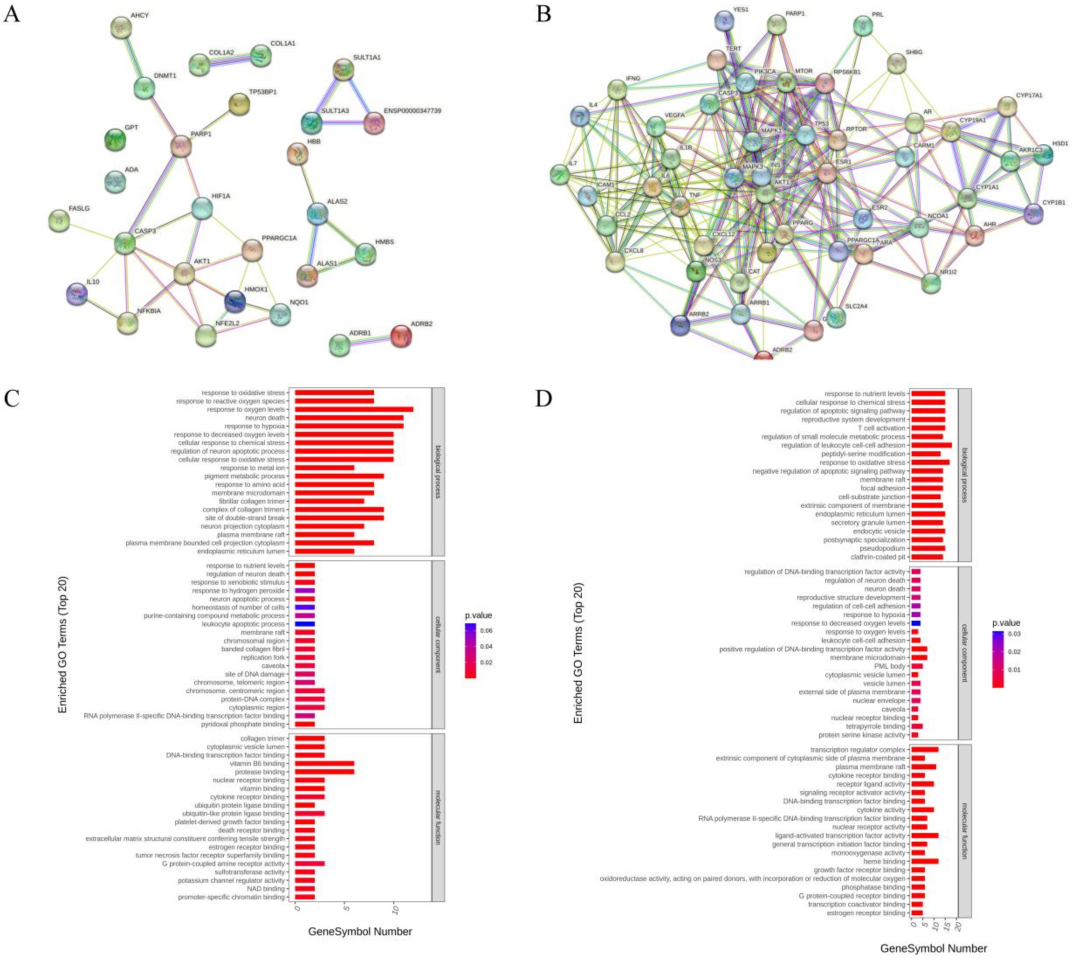

Figure S2 Top20 target, GO and KEGG enrichment analysis diagram of RR and RRP in the treatment of AD

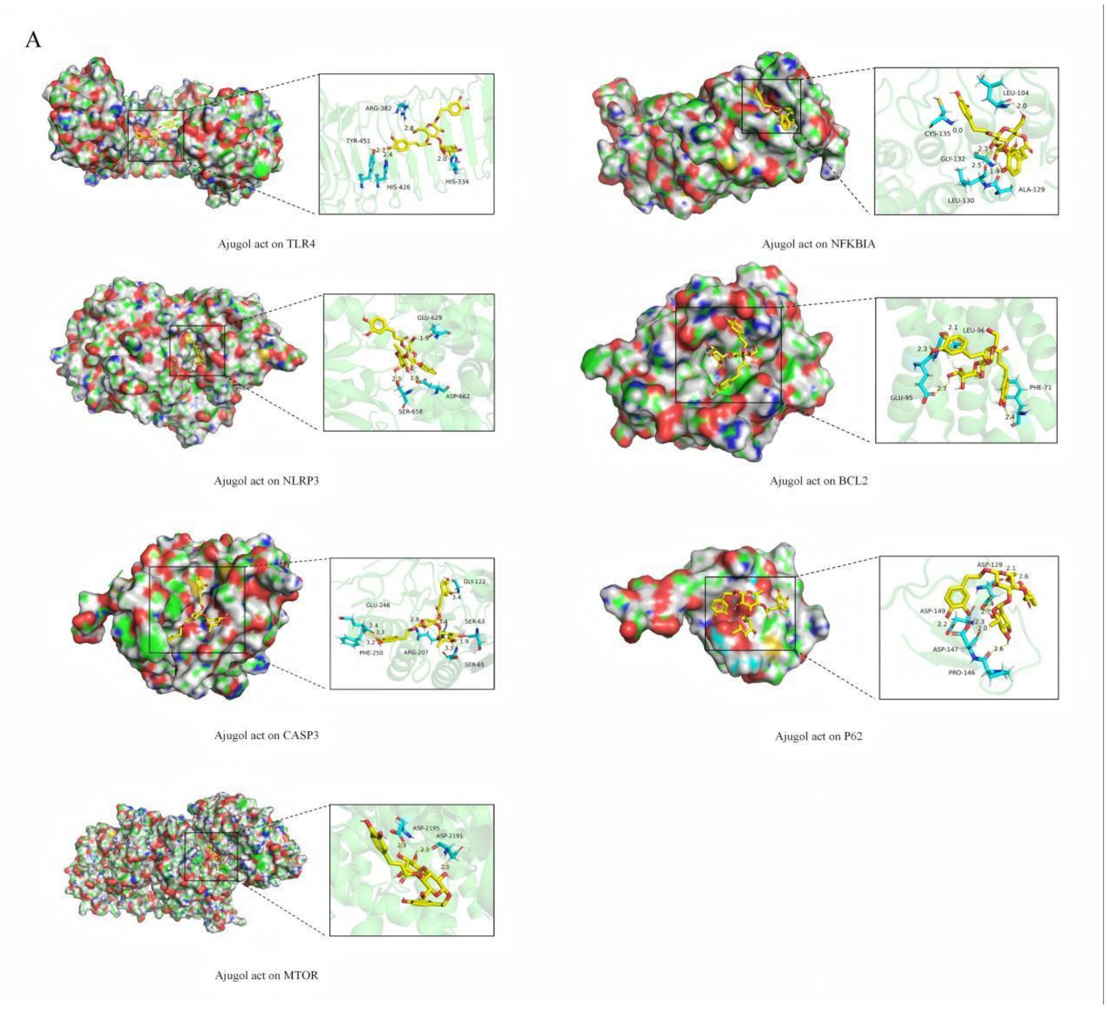

Figure S3 The results of RR, RRP active components (Verbascoside) respectively with seven kinds of proteins (TLR4, NFKBIA and NLRP3, BCL2, CASP3, P62, MTOR) molecular docking.

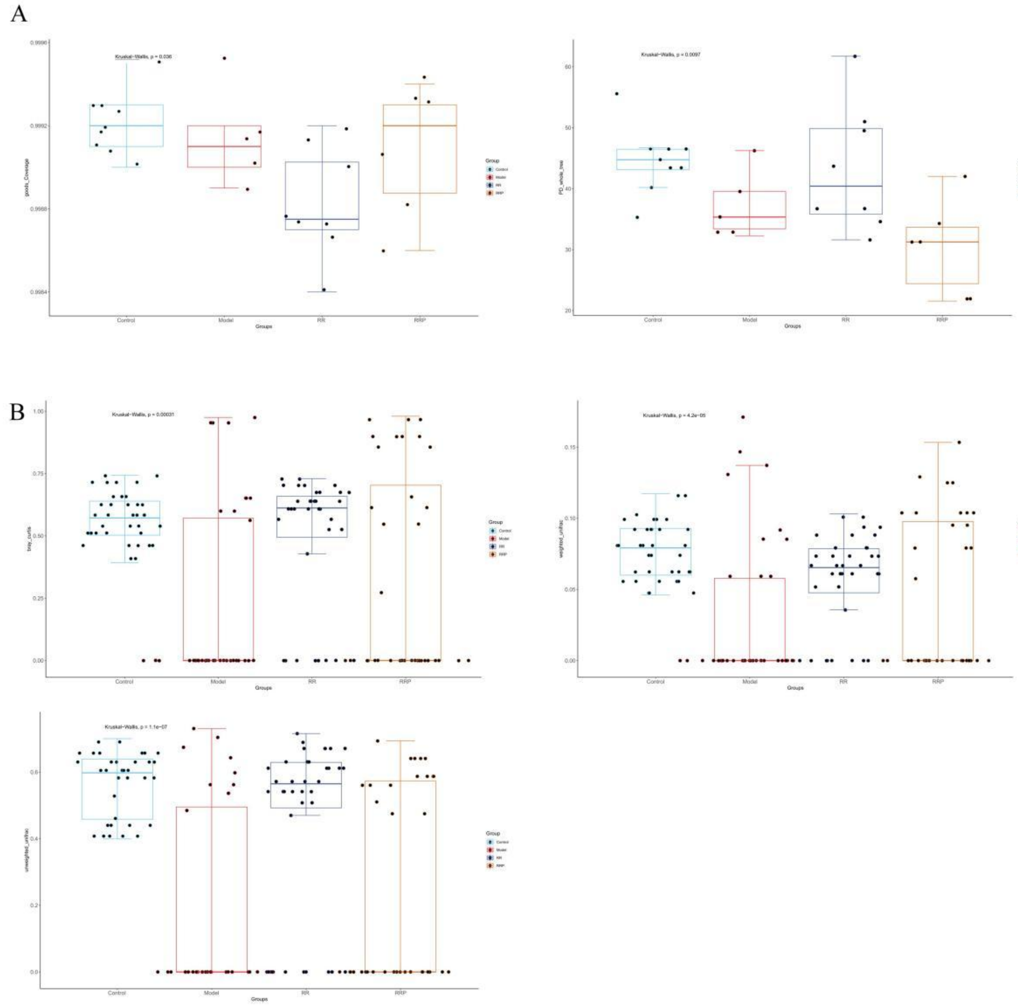

Figure S4 Effects of RR and RRP on gut microbiota in APP/PS1 mice. **A** α diversity analysis of each group (From left to right are the goods_Coverage index and PD_whole_tree index). **B** β diversity analysis of each group (From left to right are the Bray Curtis index, Unweighted UnifracBeta index and Weighted Unifrac index). The experiment data were expressed as means±SEM. *n* =3 in each group.

Supplementary information 2 The original images of the western blotting of the expression level in brains of each group for three independent repeats.

The 1st time:

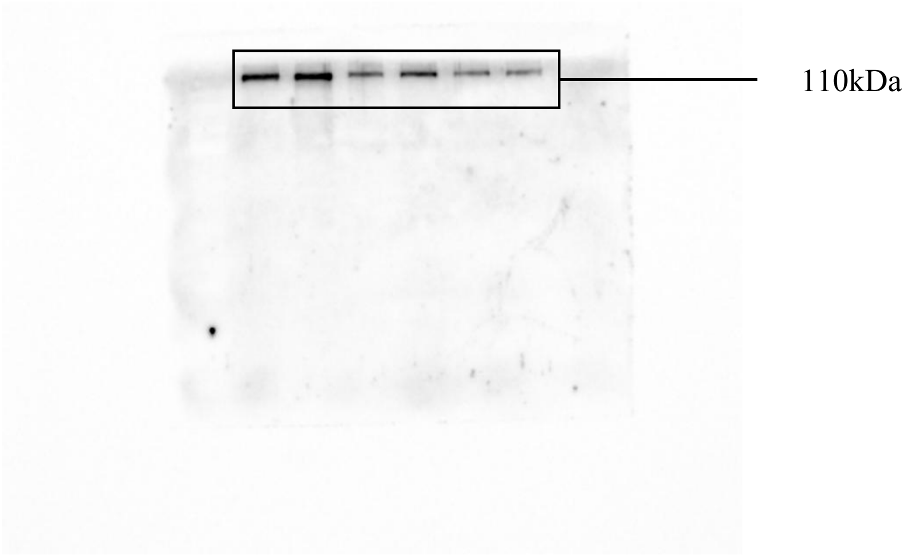

The 2nd time:

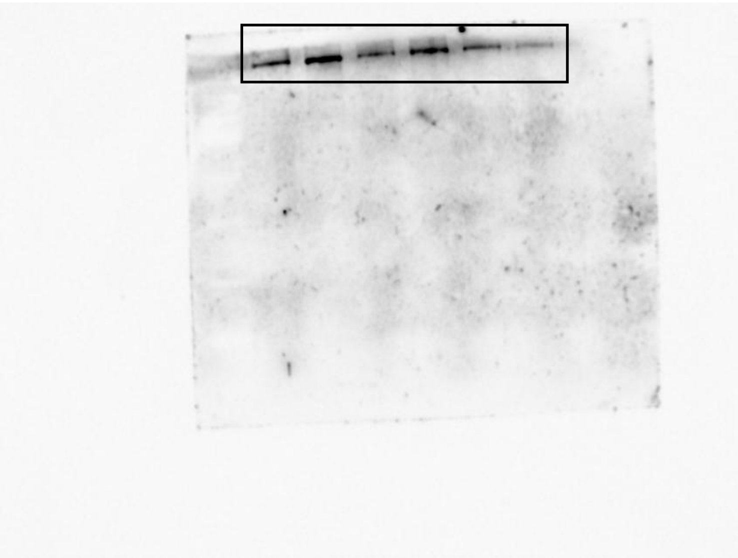

The 3 rd time:

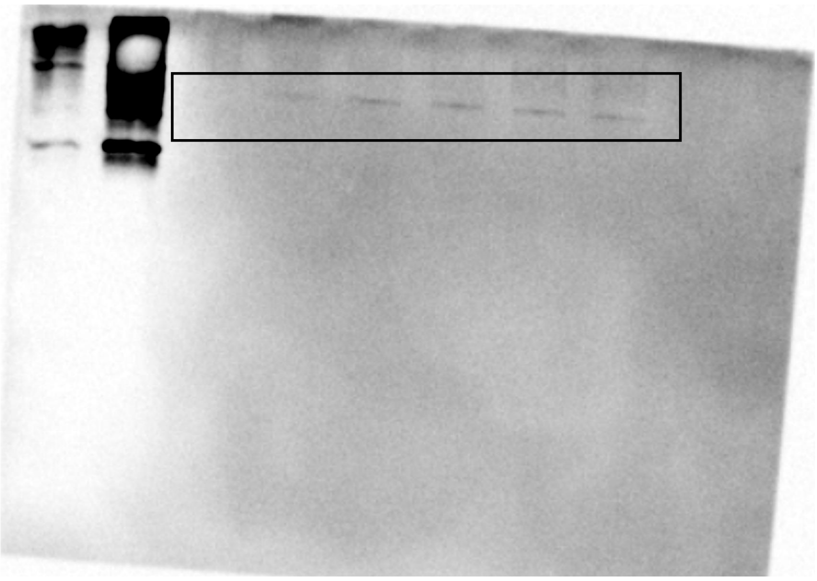

Figure 1(A)-1 The original images of the western blotting of the expression level of TLR4 in brains of each group for three independent repeats. (From left to right in the black text box are Control group, Model group, Ajugol-H group, Ajugol-Lgroup, Isoacteoside-H group, Isoacteoside-L group, respectively. The first western blotting band is presented as a representative image in the manuscript.)

The 1st time:

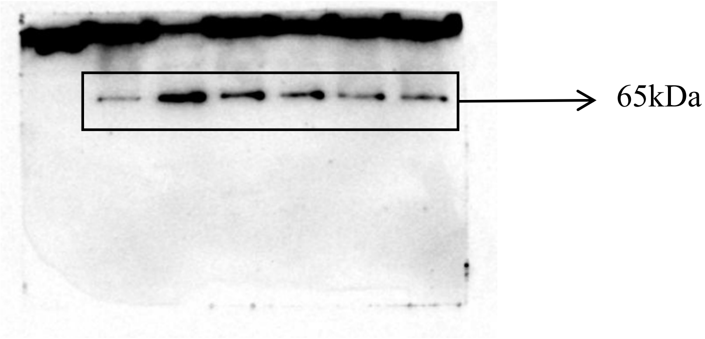

The 2nd time:

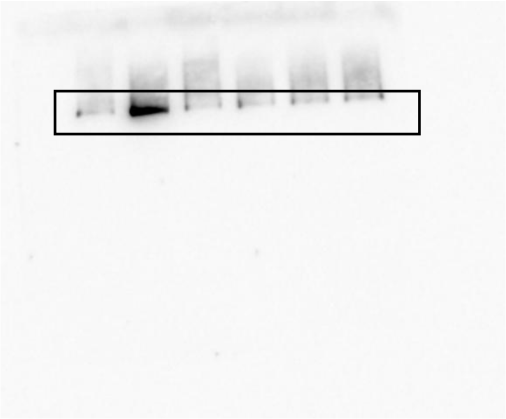

The 3 rd time:

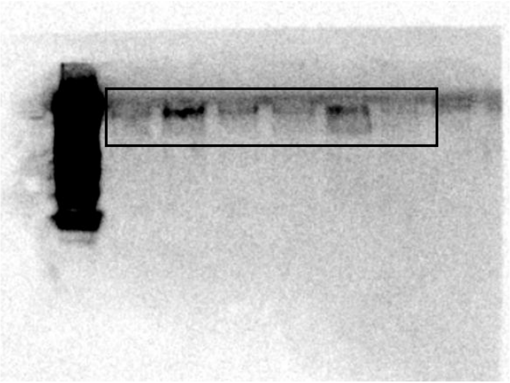

Figure 1(A)-2 The original images of the western blotting of the expression level of p-NF-κB（p65） in brains of each group for three independent repeats. (From left to right in the black text box are Control group, Model group, Ajugol-H group, Ajugol-Lgroup, Isoacteoside-H group, Isoacteoside-L group, respectively. The first western blotting band is presented as a representative image in the manuscript.)

The 1st time:

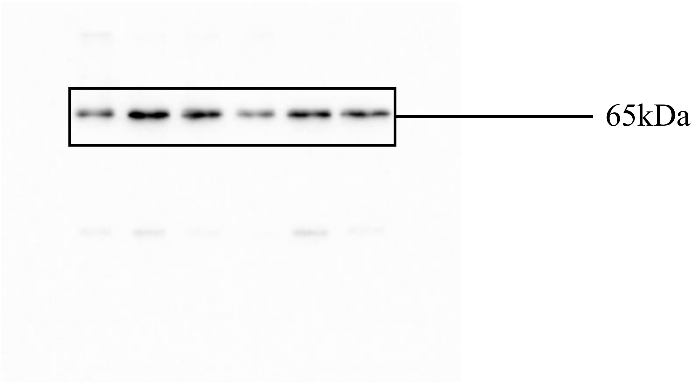

The 2nd time:

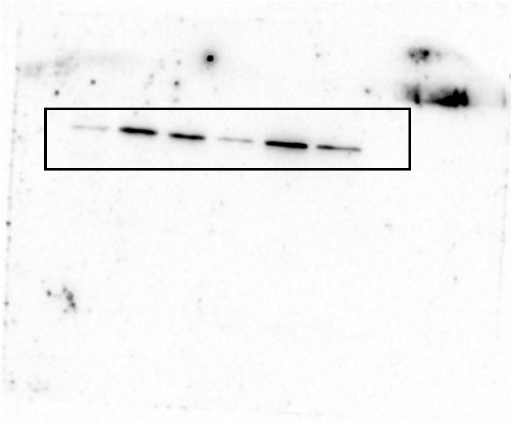

The 3 rd time:

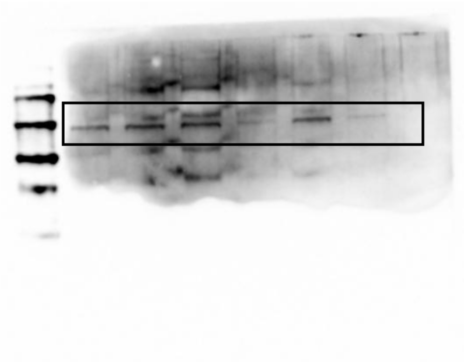

Figure 1(A)-3 The original images of the western blotting of the expression level of NF-κB（p65） in brains of each group for three independent repeats. (From left to right in the black text box are Control group, Model group, Ajugol-H group, Ajugol-Lgroup, Isoacteoside-H group, Isoacteoside-L group, respectively. The first western blotting band is presented as a representative image in the manuscript.)

The 1st time:

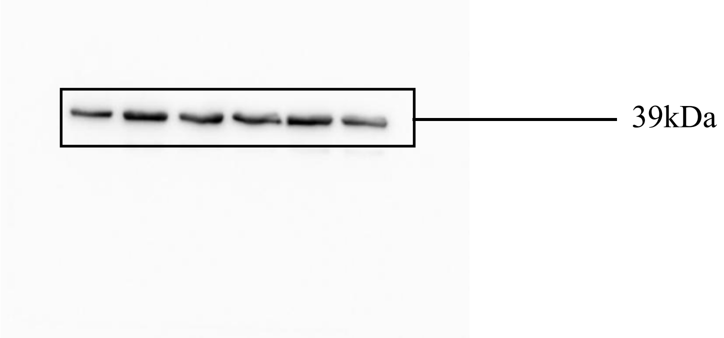

The 2nd time:

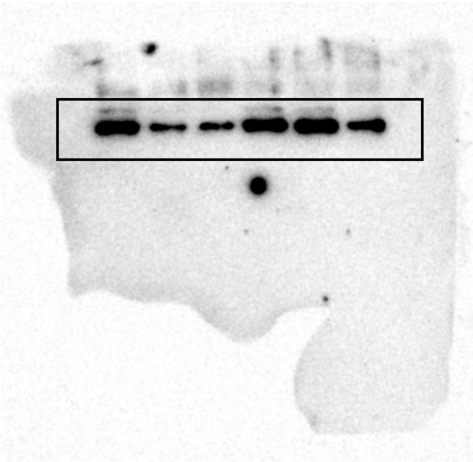

The 3 rd time:

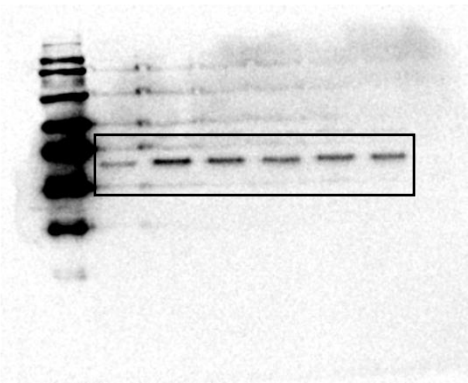

Figure 1(A)-4 The original images of the western blotting of the expression level of p-IκBα in brains of each group for three independent repeats. (From left to right in the black text box are Control group, Model group, Ajugol-H group, Ajugol-Lgroup, Isoacteoside-H group, Isoacteoside-L group, respectively. The first western blotting band is presented as a representative image in the manuscript.)

The 1st time:

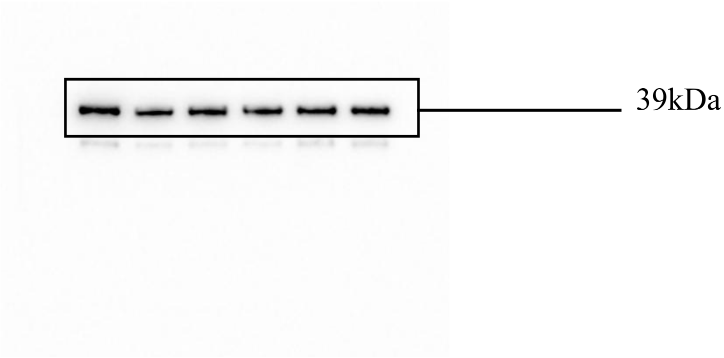

The 2nd time:

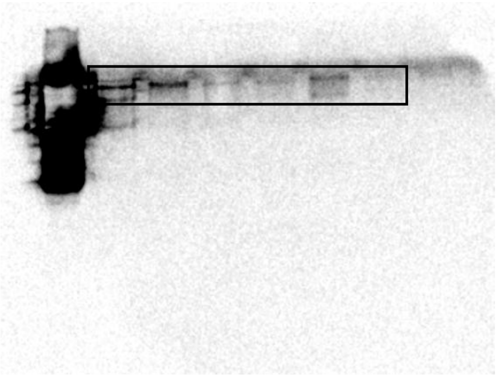

The 3 rd time:

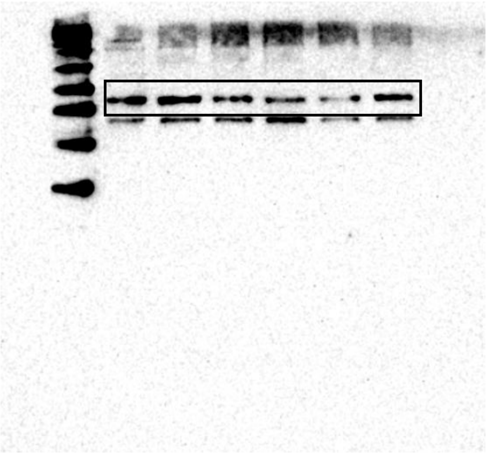

Figure 1(A)-5 The original images of the western blotting of the expression level of IκBα in brains of each group for three independent repeats. (From left to right in the black text box are Control group, Model group, Ajugol-H group, Ajugol-Lgroup, Isoacteoside-H group, Isoacteoside-L group, respectively. The first western blotting band is presented as a representative image in the manuscript.)

The 1st time:

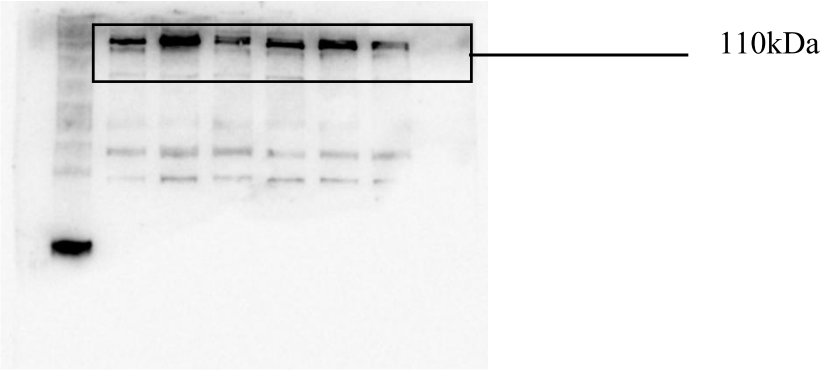

The 2nd time:

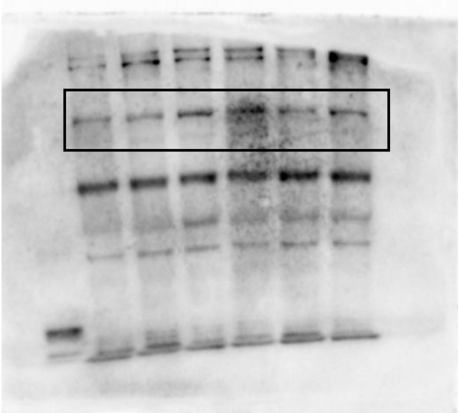

The 3 rd time:

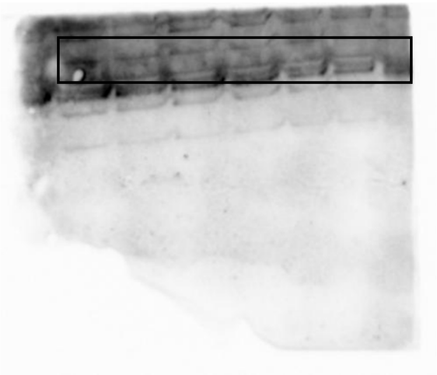

Figure 1(A)-6 The original images of the western blotting of the expression level of NLRP3 in brains of each group for three independent repeats. (From left to right in the black text box are Control group, Model group, Ajugol-H group, Ajugol-Lgroup, Isoacteoside-H group, Isoacteoside-L group, respectively. The first western blotting band is presented as a representative image in the manuscript.)

The 1st time:

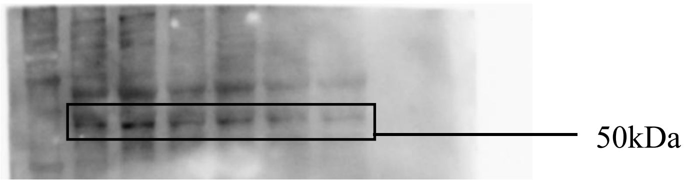

The 2nd time:

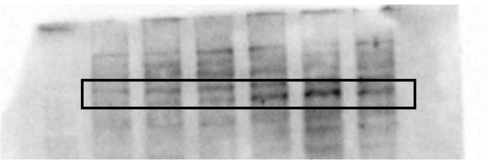

The 3 rd time:

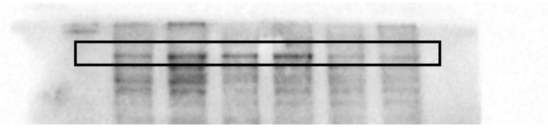

Figure 1(A)-7 The original images of the western blotting of the expression level of Caspase-1 in brains of each group for three independent repeats. (From left to right in the black text box are Control group, Model group, Ajugol-H group, Ajugol-Lgroup, Isoacteoside-H group, Isoacteoside-L group, respectively. The first western blotting band is presented as a representative image in the manuscript.)

The 1st time:

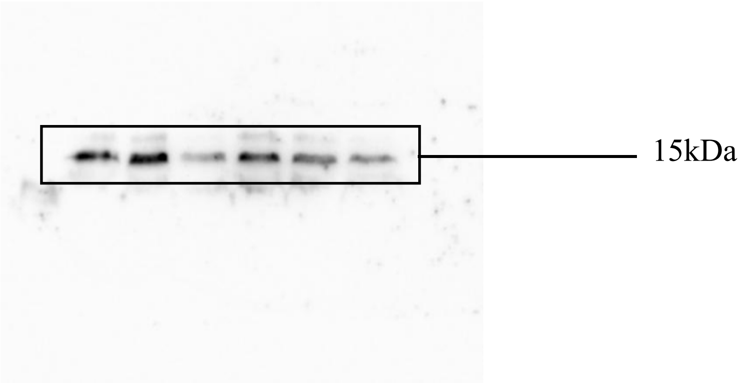

The 2nd time:

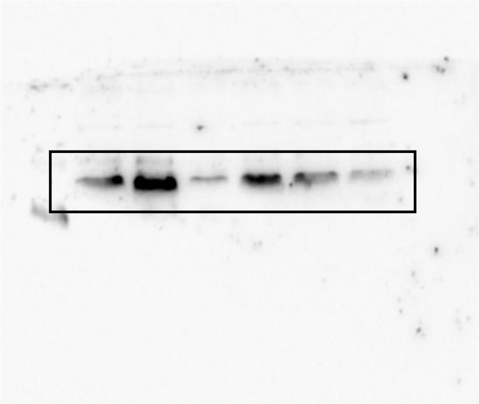

The 3 rd time:

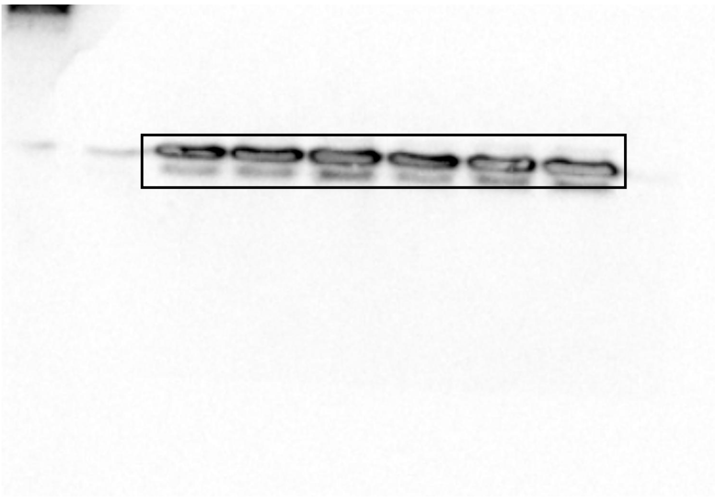

Figure 1(A)-8 The original images of the western blotting of the expression level of cytochrome C in brains of each group for three independent repeats. (From left to right in the black text box are Control group, Model group, Ajugol-H group, Ajugol-Lgroup, Isoacteoside-H group, Isoacteoside-L group, respectively. The first western blotting band is presented as a representative image in the manuscript.)

The 1st time:

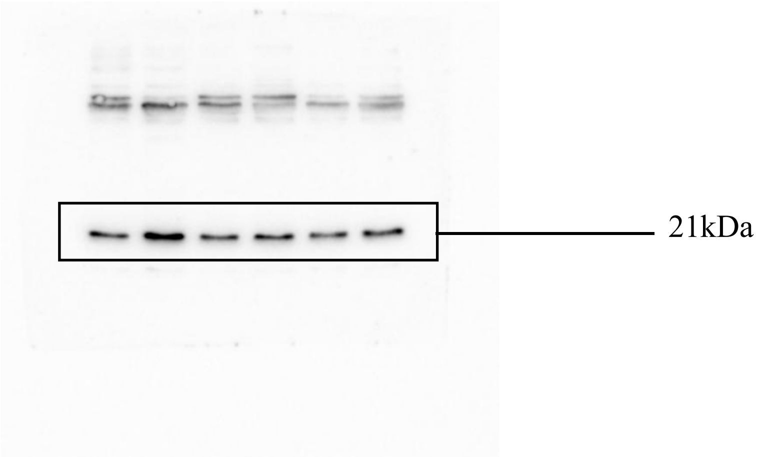

The 2nd time:

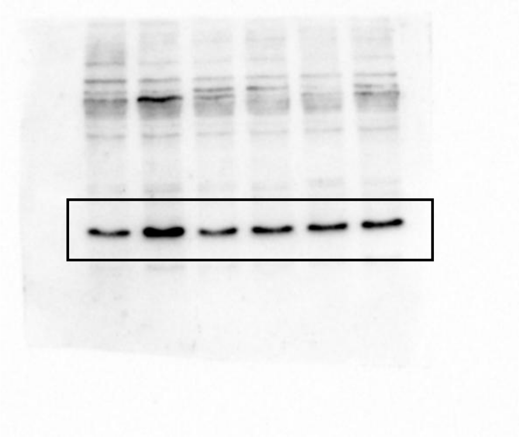

The 3 rd time:

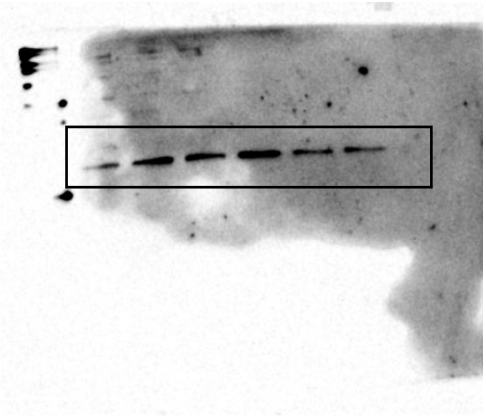

Figure 1(A)-9 The original images of the western blotting of the expression level of BAX in brains of each group for three independent repeats. (From left to right in the black text box are Control group, Model group, Ajugol-H group, Ajugol-Lgroup, Isoacteoside-H group, Isoacteoside-L group, respectively. The first western blotting band is presented as a representative image in the manuscript.)

The 1st time:

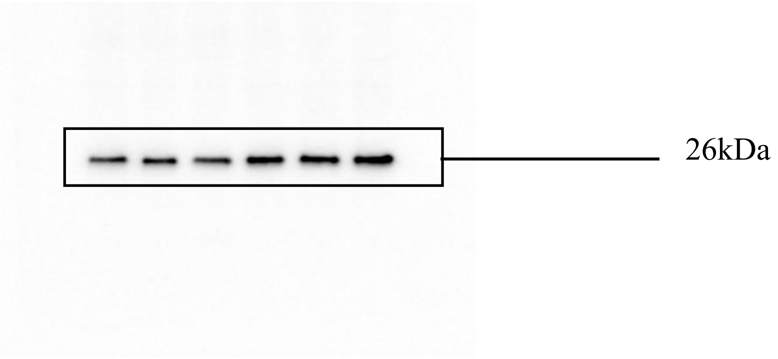

The 2nd time:

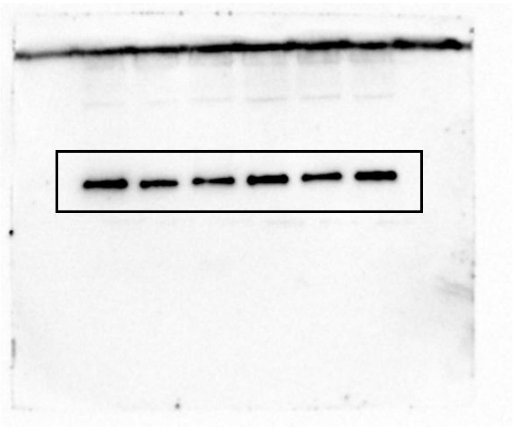

The 3 rd time:

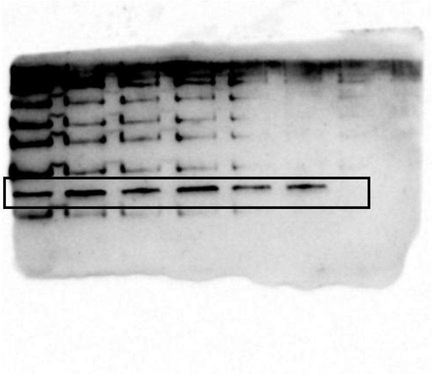

Figure 1(A)-10 The original images of the western blotting of the expression level of BCL-2 in brains of each group for three independent repeats. (From left to right in the black text box are Control group, Model group, Ajugol-H group, Ajugol-Lgroup, Isoacteoside-H group, Isoacteoside-L group, respectively. The first western blotting band is presented as a representative image in the manuscript.)

The 1st time:

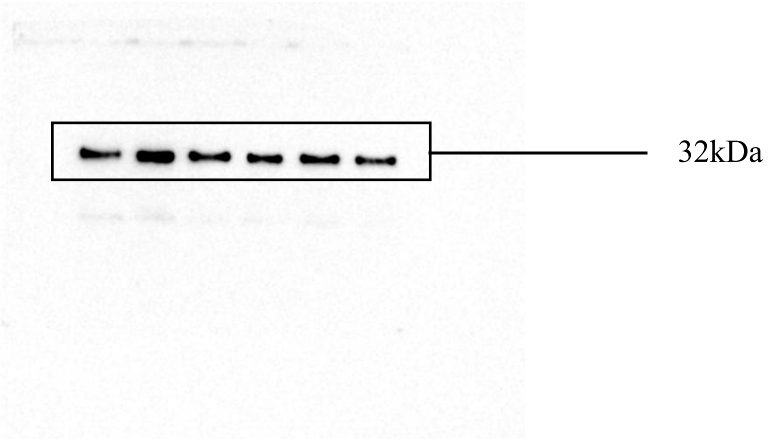

The 2nd time:

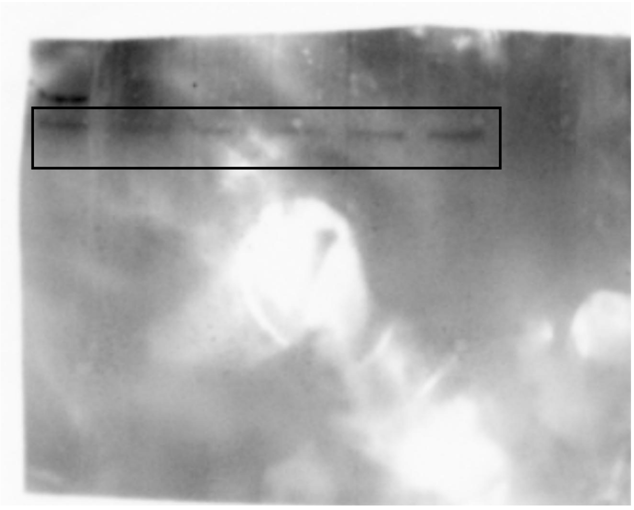

The 3 rd time:

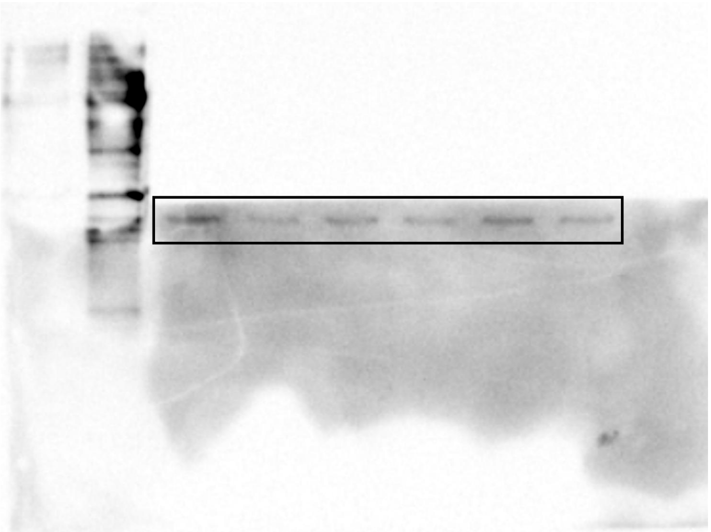

Figure 1(A)-11 The original images of the western blotting of the expression level of

caspase-3 in brains of each group for three independent repeats. (From left to right in the black text box are Control group, Model group, Ajugol-H group, Ajugol-Lgroup, Isoacteoside-H group, Isoacteoside-L group, respectively. The first western blotting band is presented as a representative image in the manuscript.)

The 1st time:

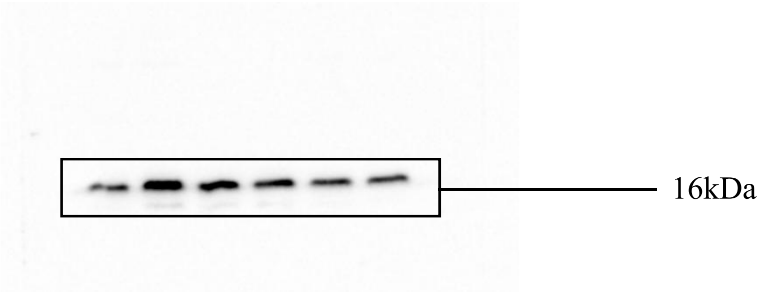

16kDa

The 2nd time:

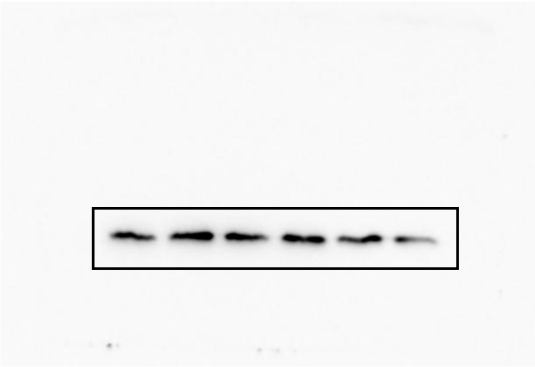

The 3 rd time:

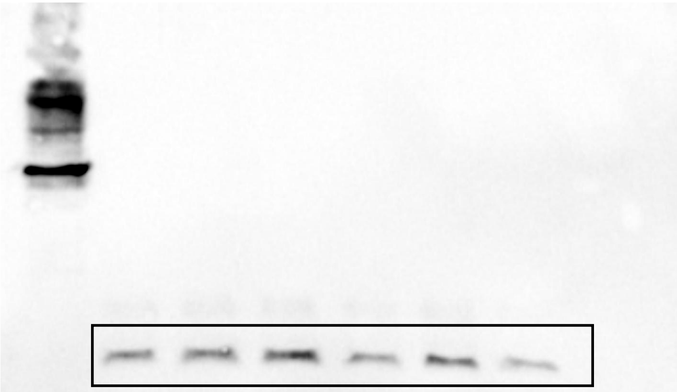

Figure 1(A)-12 The original images of the western blotting of the expression level of LC3-II in brains of each group for three independent repeats. (From left to right in the

black text box are Control group, Model group, Ajugol-H group, Ajugol-Lgroup, Isoacteoside-H group, Isoacteoside-L group, respectively. The first western blotting band is presented as a representative image in the manuscript.)

The 1st time:

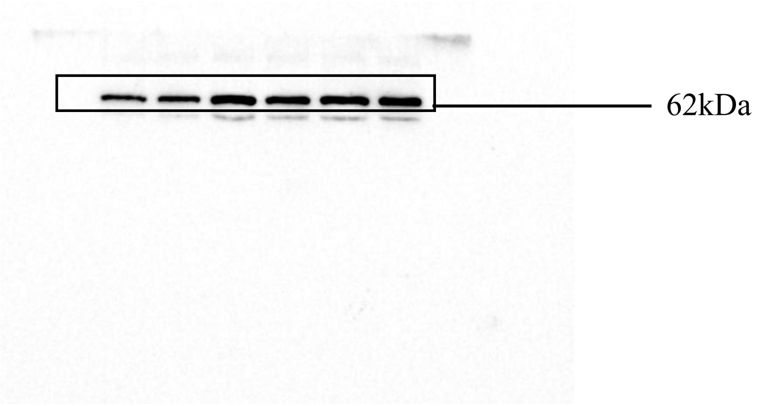

The 2nd time:

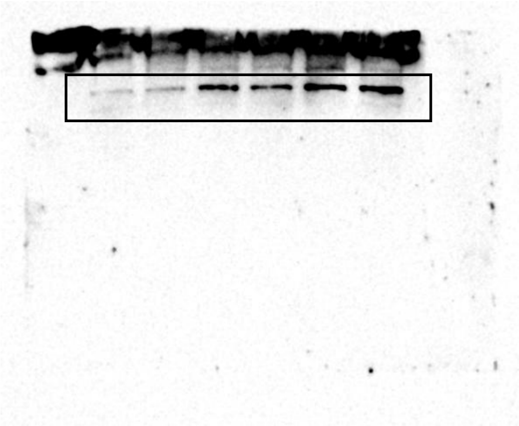

The 3 rd time:

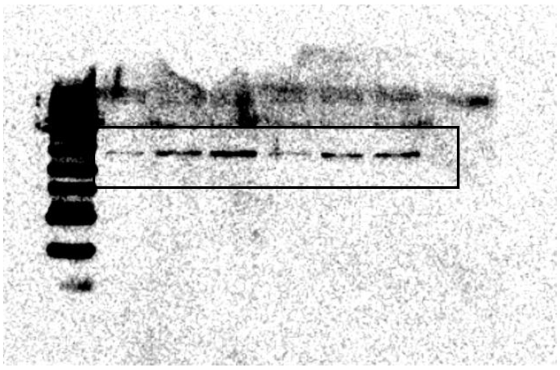

Figure 1(A)-13 The original images of the western blotting of the expression level of P62 in brains of each group for three independent repeats. (From left to right in the black text box are Control group, Model group, Ajugol-H group, Ajugol-Lgroup,

Isoacteoside-H group, Isoacteoside-L group, respectively. The first western blotting band is presented as a representative image in the manuscript.)

The 1st time:

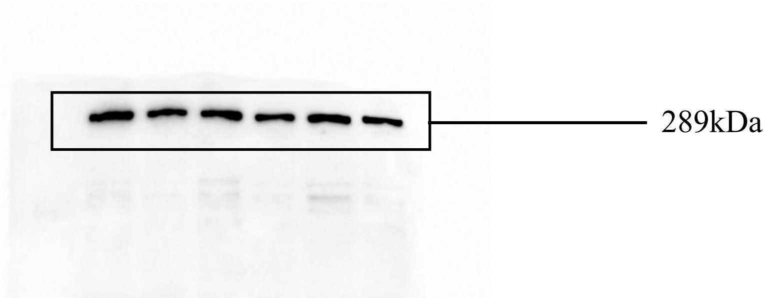

The 2nd time:

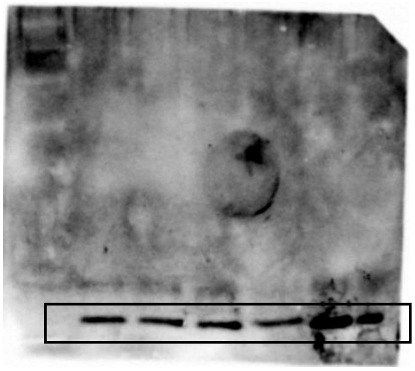

The 3 rd time:

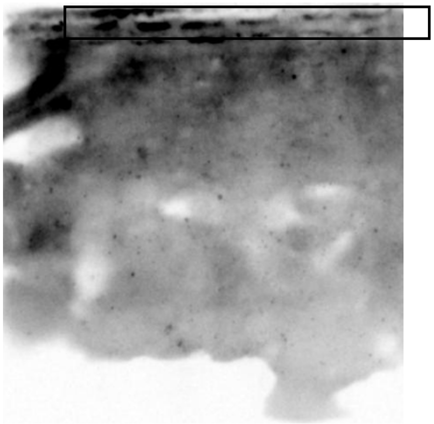

Figure 1(A)-14 The original images of the western blotting of the expression level of p-mTOR in brains of each group for three independent repeats. (From left to right in the black text box are Control group, Model group, Ajugol-H group, Ajugol-Lgroup, Isoacteoside-H group, Isoacteoside-L group, respectively. The first western blotting

band is presented as a representative image in the manuscript.)

The 1st time:

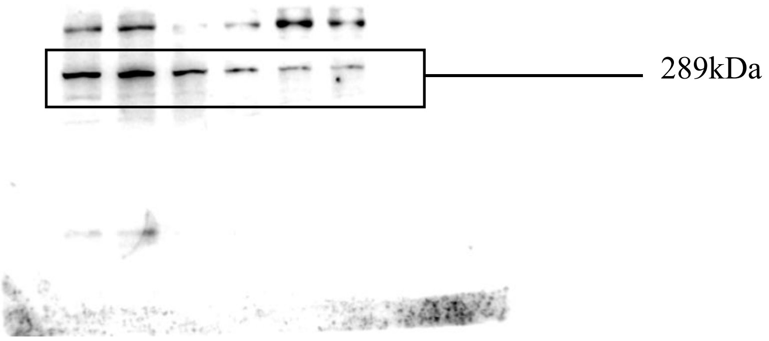

The 2nd time:

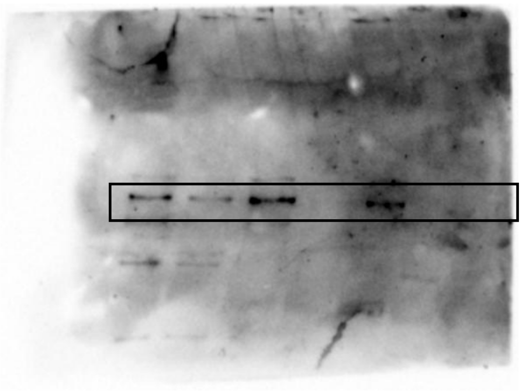

The 3rd time:

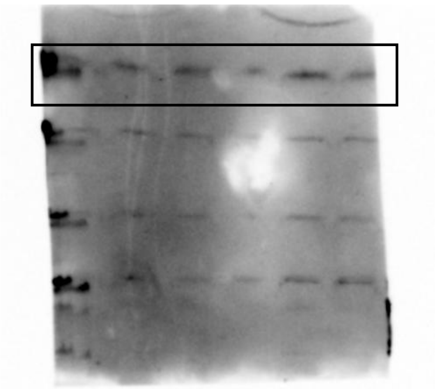

Figure 1(A)-15 The original images of the western blotting of the expression level of mTOR in brains of each group for three independent repeats. (From left to right in the black text box are Control group, Model group, Ajugol-H group, Ajugol-Lgroup,

Isoacteoside-H group, Isoacteoside-L group, respectively. The first western blotting band is presented as a representative image in the manuscript.)

The 1st time:

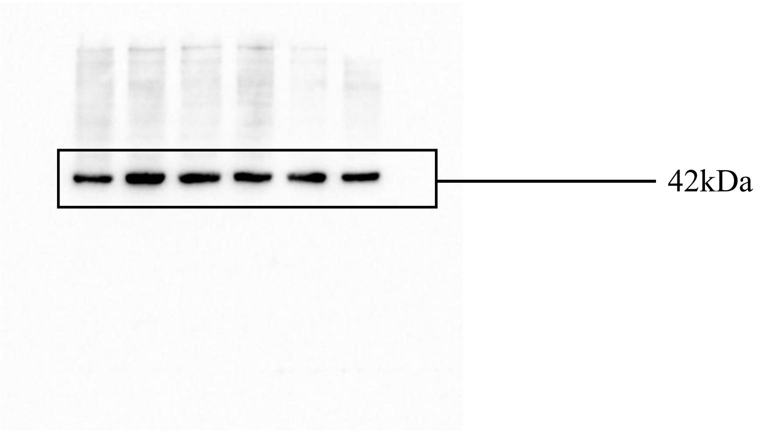

The 2nd time:

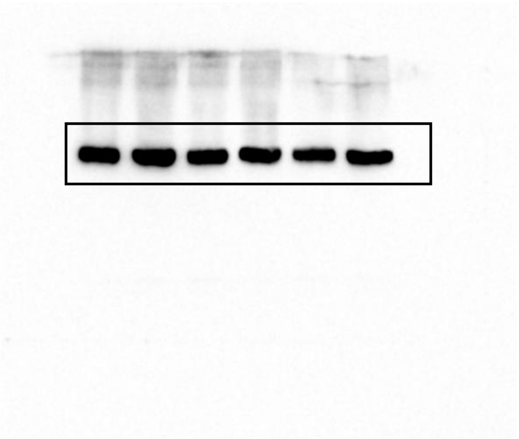

The 3 rd time:

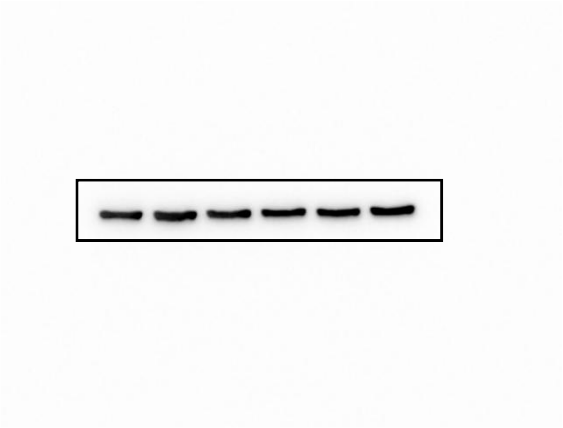

Figure 1(A)-16 The original images of the western blotting of the expression level of β-actin in brains of each group for three independent repeats. (From left to right in the black text box are Control group, Model group, Ajugol-H group, Ajugol-Lgroup, Isoacteoside-H group, Isoacteoside-L group, respectively. The first western blotting band is presented as a representative image in the manuscript.)

Supplementary information 3 The original images of the western blotting of the expression level in BV2 cells of each group for three independent repeats.

The 1st time:

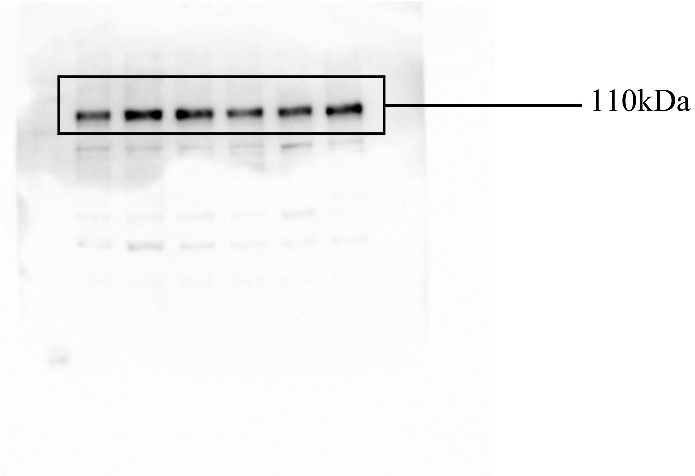

The 2nd time:

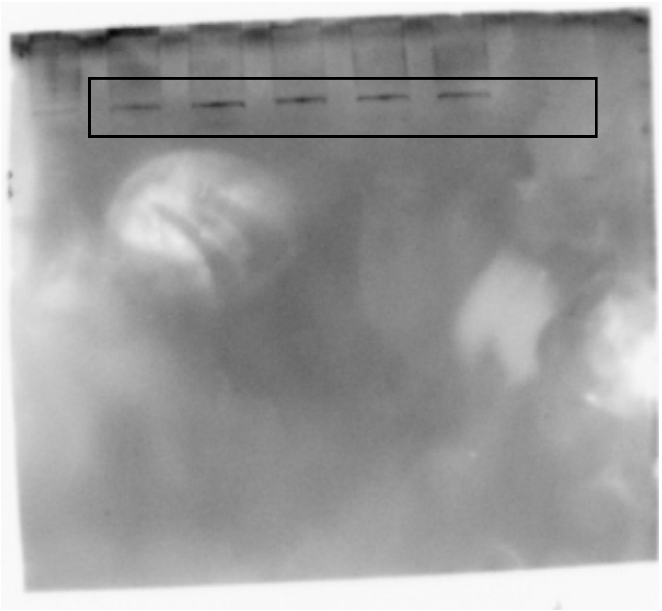

The 3 rd time:

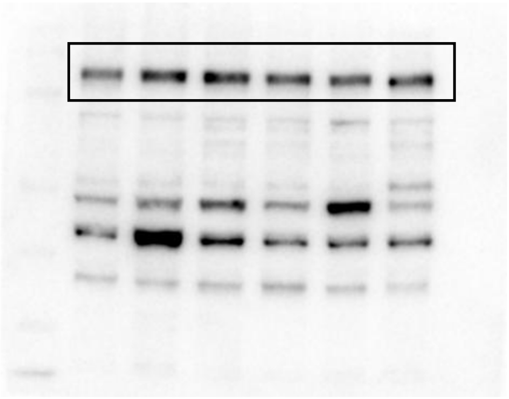

Figure 1(A)-1 The original images of the western blotting of the expression level of TLR4 in BV2 cells of each group for three independent repeats. (From left to right in the black text box are Control group, Model group, Ajugol-H group, Ajugol-Lgroup, Isoacteoside-H group, Isoacteoside-L group, respectively. The first western blotting band is presented as a representative image in the manuscript.)

The 1st time:

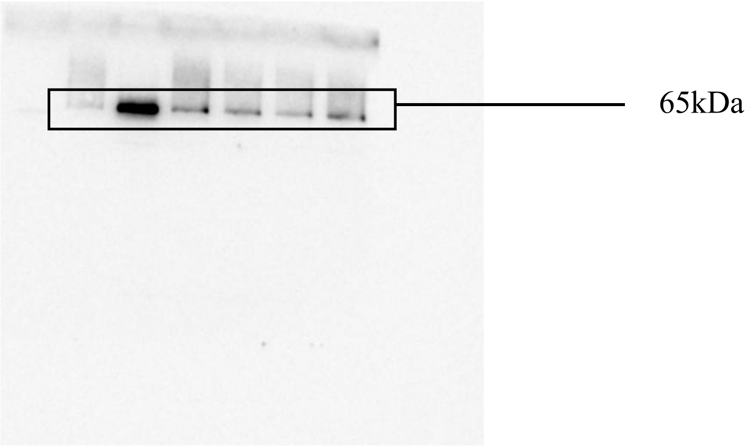

65kDa

The 2nd time:

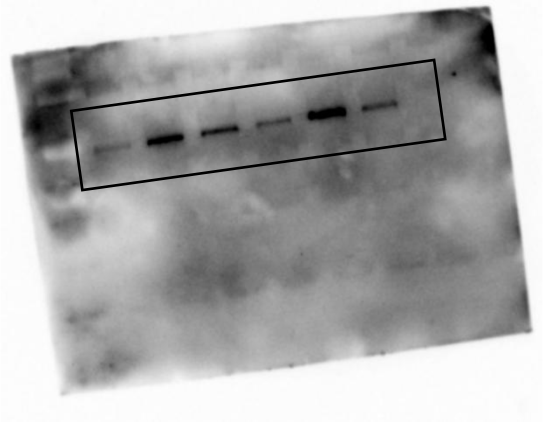

The 3 rd time:

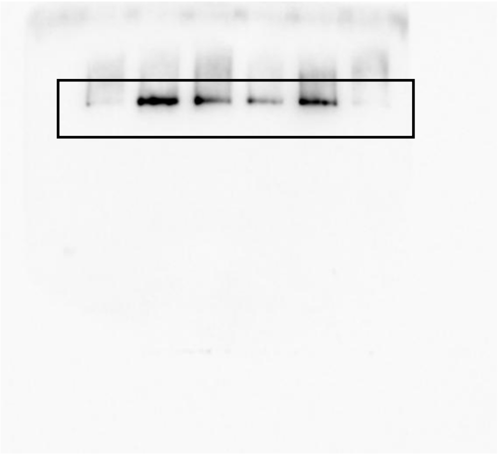

Figure 1(A)-2 The original images of the western blotting of the expression level of p-NF-κB(p65) in BV2 cells of each group for three independent repeats. (From left to right in the black text box are Control group, Model group, Ajugol-H group, Ajugol-Lgroup, Isoacteoside-H group, Isoacteoside-L group, respectively. The first western blotting band is presented as a representative image in the manuscript.)

The 1st time:

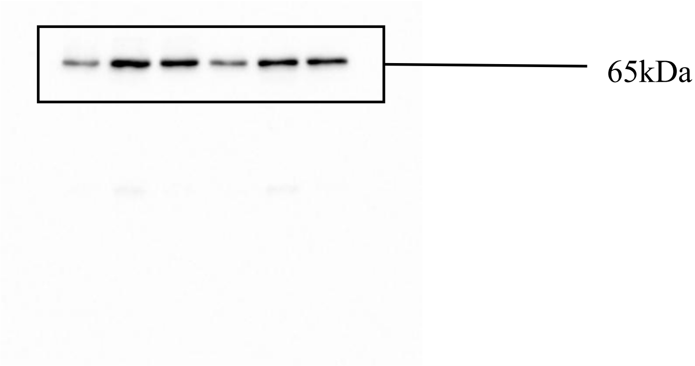

The 2nd time:

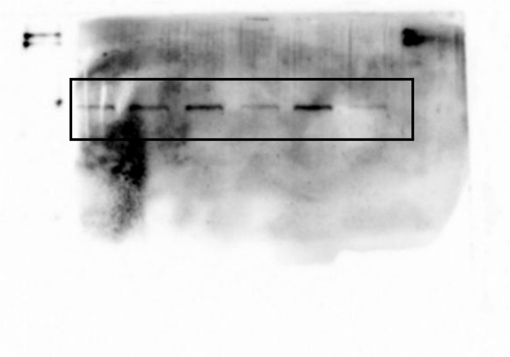

The 3 rd time:

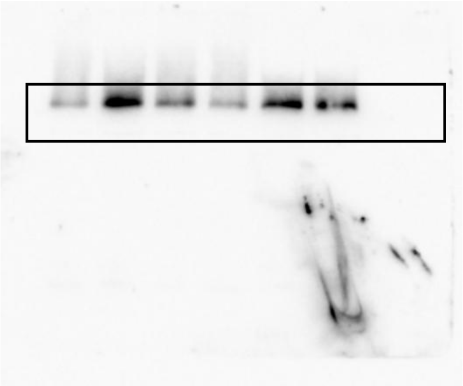

Figure 1(A)-3 The original images of the western blotting of the expression level of NF-κB(p65) in BV2 cells of each group for three independent repeats. (From left to right in the black text box are Control group, Model group, Ajugol-H group, Ajugol-Lgroup, Isoacteoside-H group, Isoacteoside-L group, respectively. The first western blotting band is presented as a representative image in the manuscript.)

The 1st time:

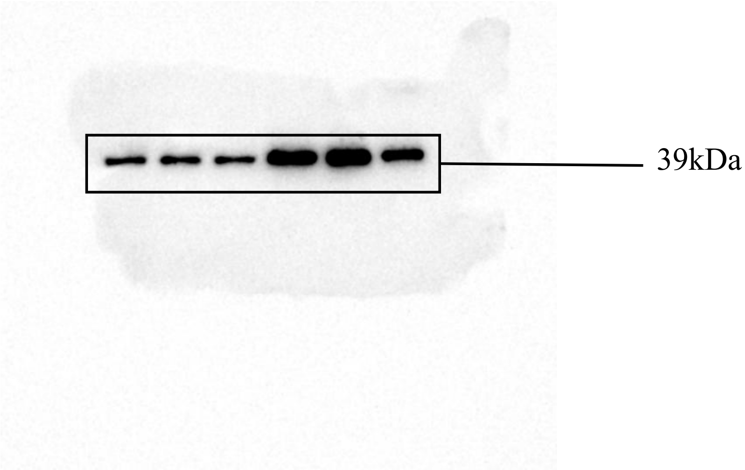

The 2nd time:

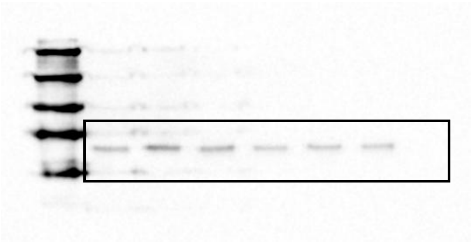

The 3 rd time:

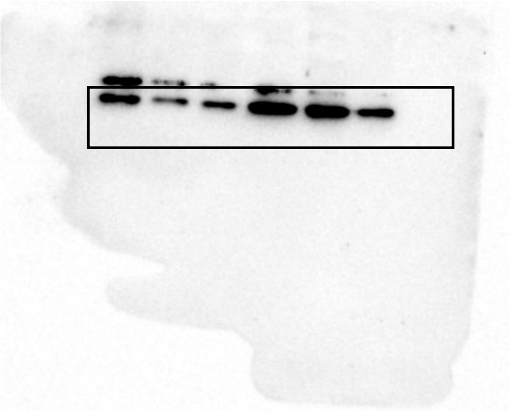

Figure 1(A)-4 The original images of the western blotting of the expression level of p-IκBα in BV2 cells of each group for three independent repeats. (From left to right in the black text box are Control group, Model group, Ajugol-H group, Ajugol-Lgroup, Isoacteoside-H group, Isoacteoside-L group, respectively. The first western blotting band is presented as a representative image in the manuscript.)

The 1st time:

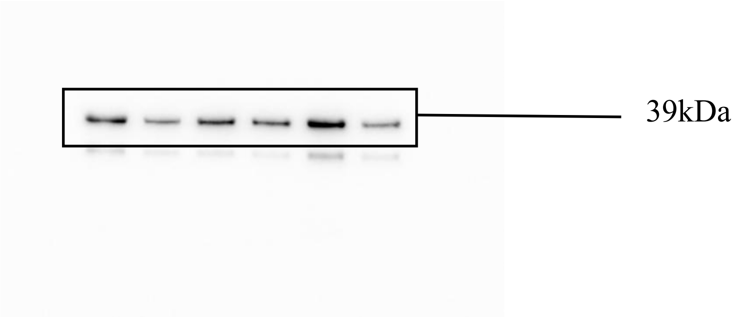

The 2nd time:

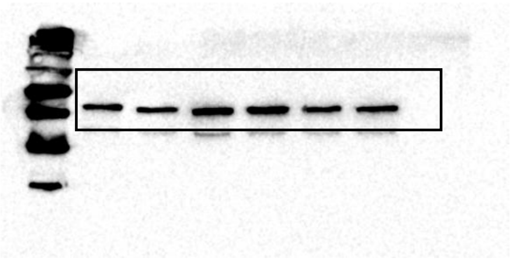

The 3 rd time:

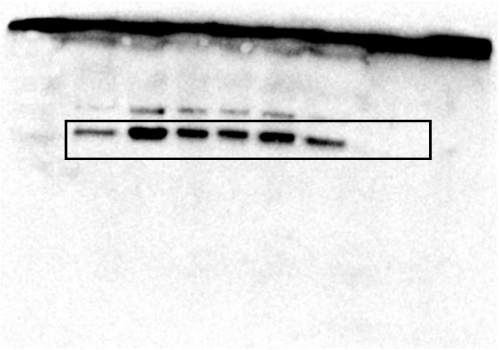

Figure 1(A)-5 The original images of the western blotting of the expression level of IκBα in BV2 cells of each group for three independent repeats. (From left to right in the black text box are Control group, Model group, Ajugol-H group, Ajugol-Lgroup, Isoacteoside-H group, Isoacteoside-L group, respectively. The first western blotting band is presented as a representative image in the manuscript.)

The 1st time:

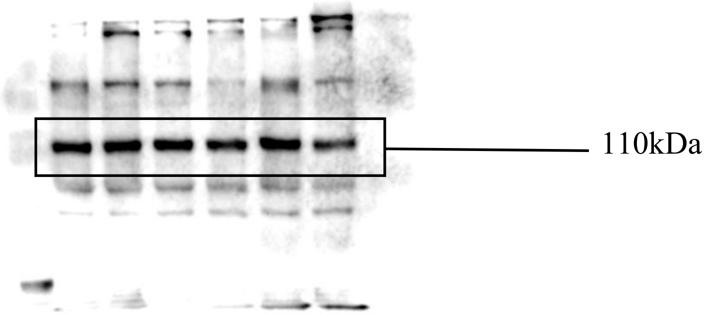

The 2nd time:？

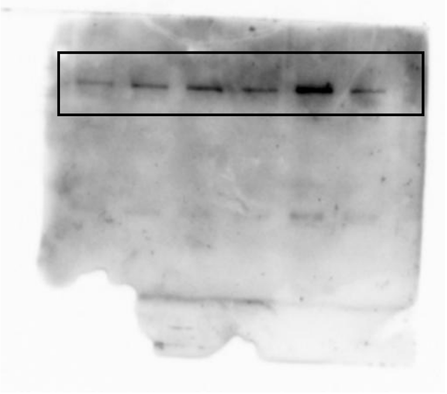

The 3 rd time:

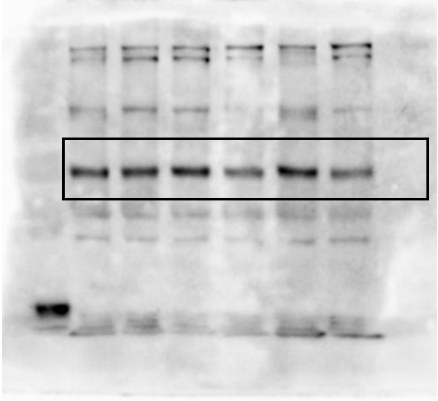

Figure 1(A)-6 The original images of the western blotting of the expression level of NLRP3 in BV2 cells of each group for three independent repeats. (From left to right in the black text box are Control group, Model group, Ajugol-H group, Ajugol-Lgroup, Isoacteoside-H group, Isoacteoside-L group, respectively. The first western blotting band is presented as a representative image in the manuscript.)

The 1st time:

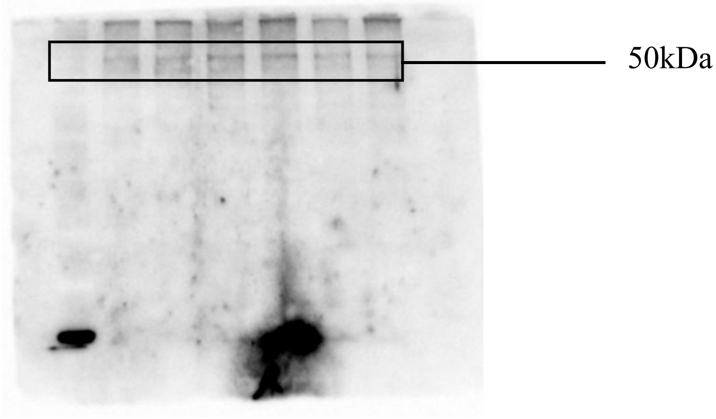

The 2nd time:

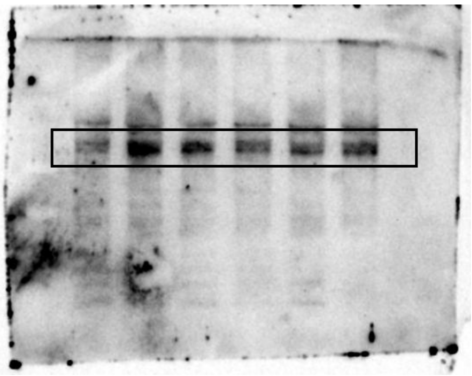

The 3 rd time:

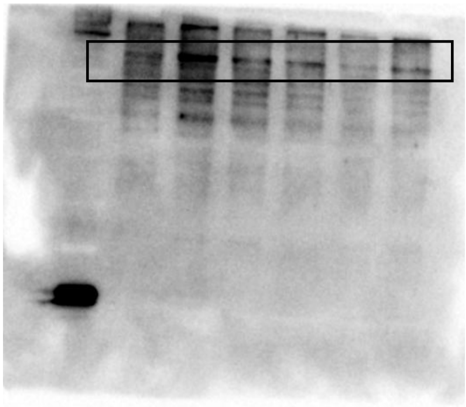

Figure 1(A)-7 The original images of the western blotting of the expression level of Caspase-1 in BV2 cells of each group for three independent repeats. (From left to right in the black text box are Control group, Model group, Ajugol-H group, Ajugol-Lgroup, Isoacteoside-H group, Isoacteoside-L group, respectively. The first western blotting band is presented as a representative image in the manuscript.)

The 1st time:

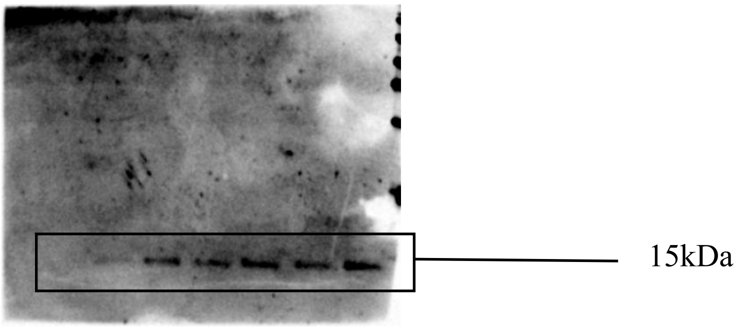

The 2nd time:

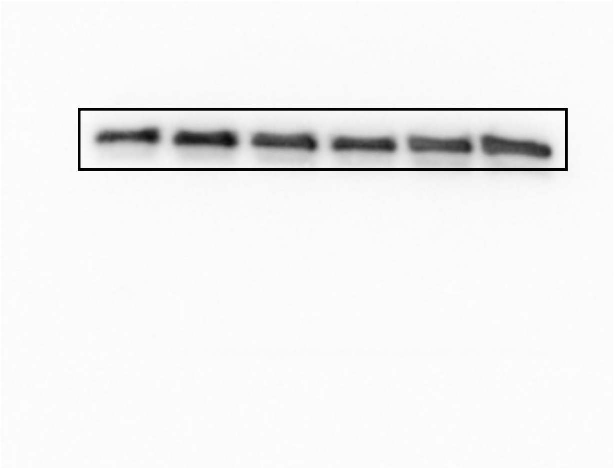

The 3 rd time:

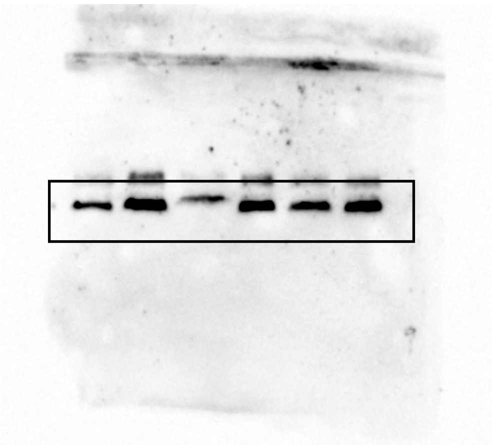

Figure 1(A)-8 The original images of the western blotting of the expression level of cytochrome C in BV2 cells of each group for three independent repeats. (From left to right in the black text box are Control group, Model group, Ajugol-H group, Ajugol-Lgroup, Isoacteoside-H group, Isoacteoside-L group, respectively. The first western blotting band is presented as a representative image in the manuscript.)

The 1st time:

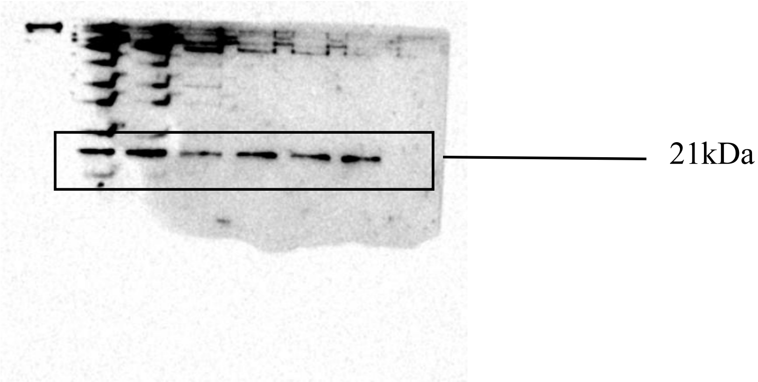

The 2nd time:

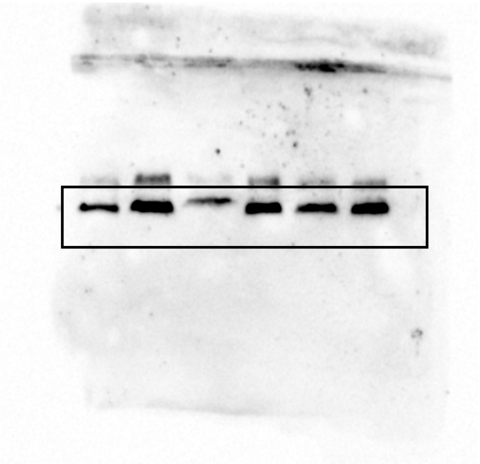

The 3 rd time:

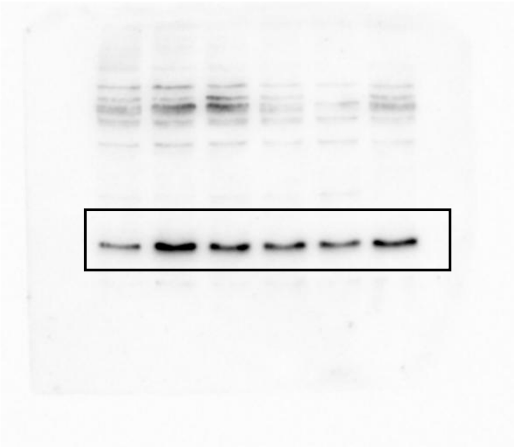

Figure 1(A)-9 The original images of the western blotting of the expression level of BAX in BV2 cells of each group for three independent repeats. (From left to right in the black text box are Control group, Model group, Ajugol-H group, Ajugol-Lgroup, Isoacteoside-H group, Isoacteoside-L group, respectively. The first western blotting band is presented as a representative image in the manuscript.)

The 1st time:

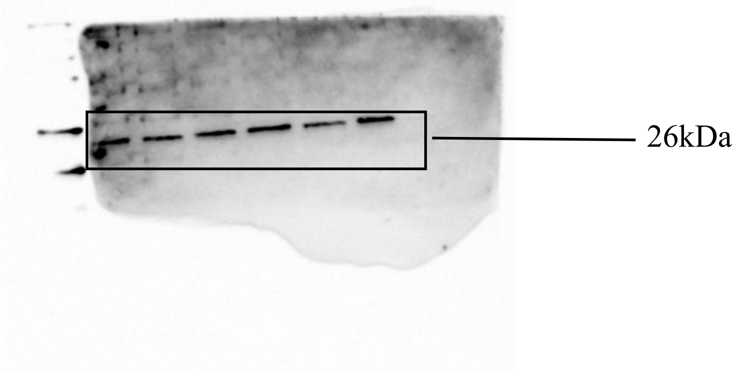

The 2nd time:

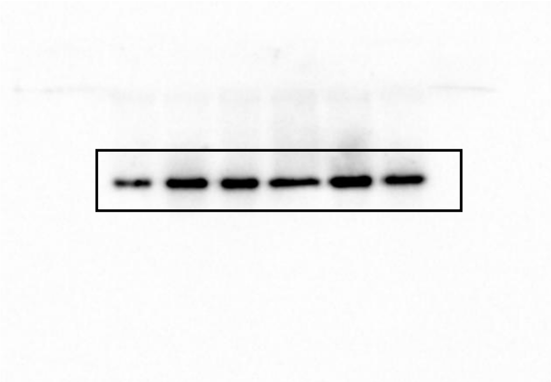

The 3 rd time:

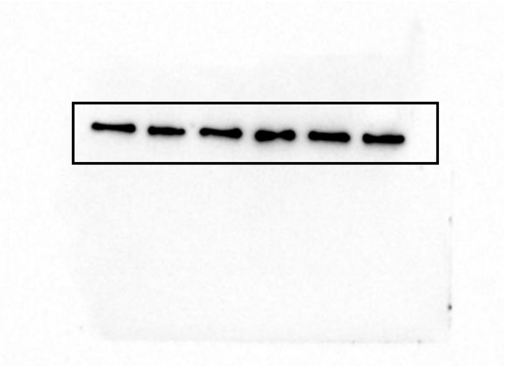

Figure 1(A)-10 The original images of the western blotting of the expression level of BCL-2 in BV2 cells of each group for three independent repeats. (From left to right in the black text box are Control group, Model group, Ajugol-H group, Ajugol-Lgroup, Isoacteoside-H group, Isoacteoside-L group, respectively. The first western blotting band is presented as a representative image in the manuscript.)

The 1st time:

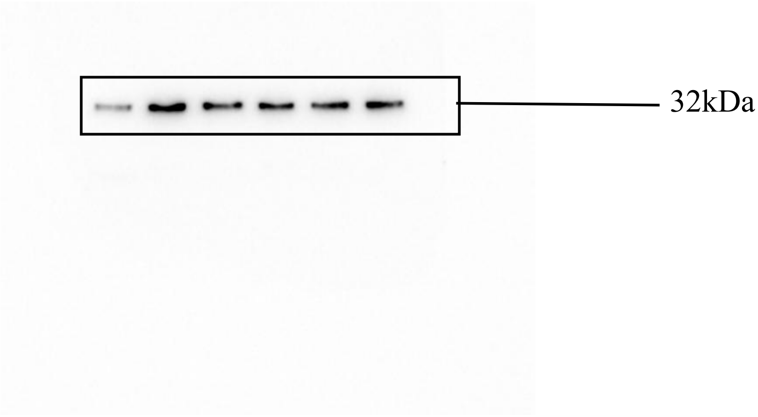

32kDa

The 2nd time:

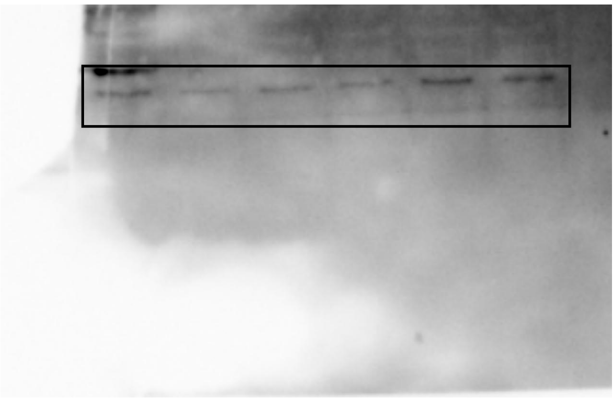

The 3 rd time:

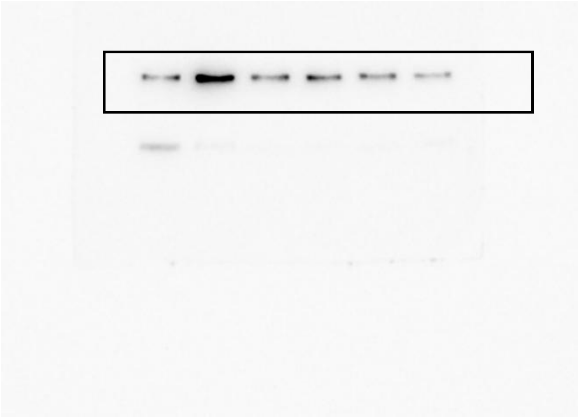

Figure 1(A)-11 The original images of the western blotting of the expression level of caspase-3 in BV2 cells of each group for three independent repeats. (From left to right in the black text box are Control group, Model group, Ajugol-H group, Ajugol-Lgroup, Isoacteoside-H group, Isoacteoside-L group, respectively. The first western blotting band is presented as a representative image in the manuscript.)

The 1st time:

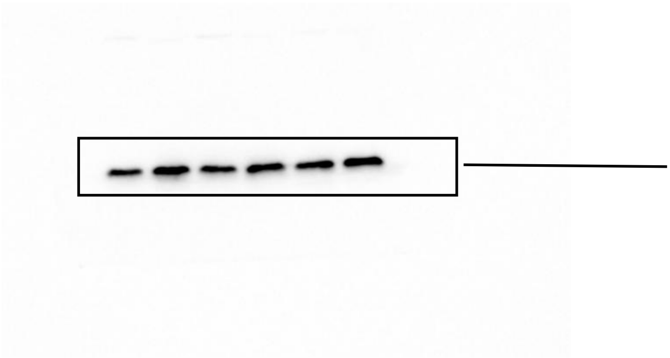

The 2nd time:

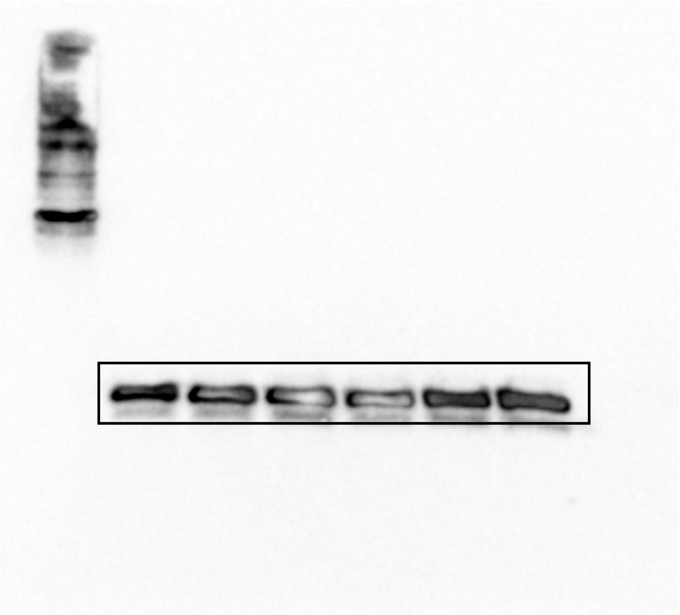

The 3 rd time:

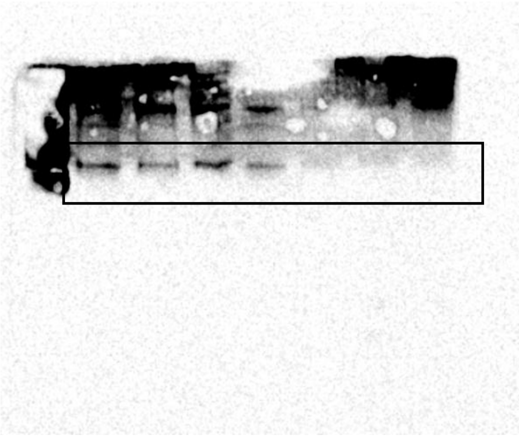

Figure 1(A)-12 The original images of the western blotting of the expression level of LC3-II in BV2 cells of each group for three independent repeats. (From left to right in the black text box are Control group, Model group, Ajugol-H group, Ajugol-Lgroup, Isoacteoside-H group, Isoacteoside-L group, respectively. The first western blotting band is presented as a representative image in the manuscript.)

The 1st time:

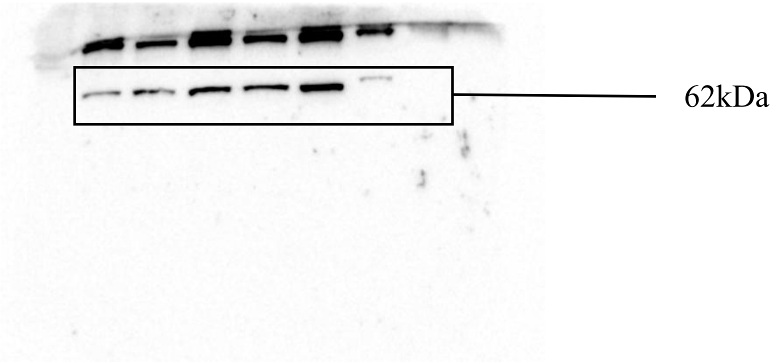

The 2nd time:

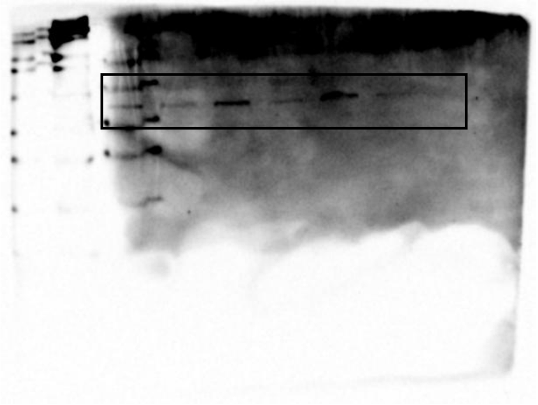

The 3 rd time:

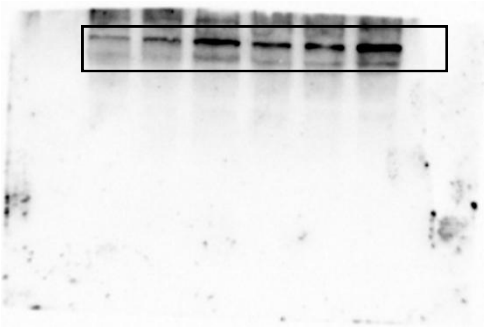

Figure 1(A)-13 The original images of the western blotting of the expression level of P62 in BV2 cells of each group for three independent repeats. (From left to right in the black text box are Control group, Model group, Ajugol-H group, Ajugol-Lgroup, Isoacteoside-H group, Isoacteoside-L group, respectively. The first western blotting band is presented as a representative image in the manuscript.)

The 1st time:

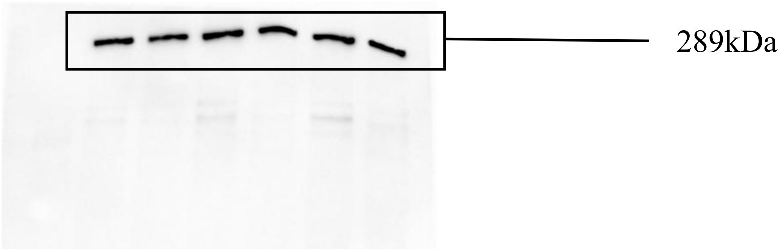

The 2nd time:

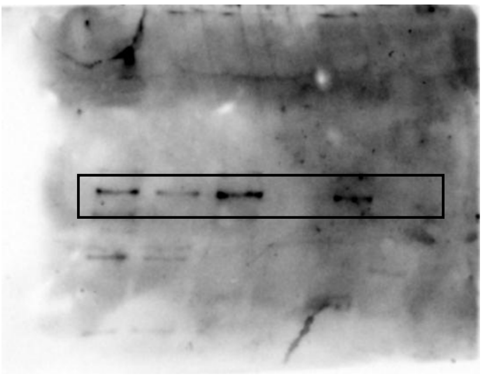

The 3 rd time:

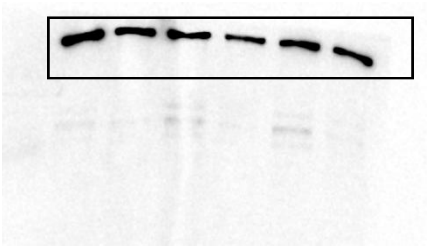

Figure 1(A)-14 The original images of the western blotting of the expression level of p-mTOR in BV2 cells of each group for three independent repeats. (From left to right in the black text box are Control group, Model group, Ajugol-H group, Ajugol-Lgroup, Isoacteoside-H group, Isoacteoside-L group, respectively. The first western blotting band is presented as a representative image in the manuscript.)

The 1st time:

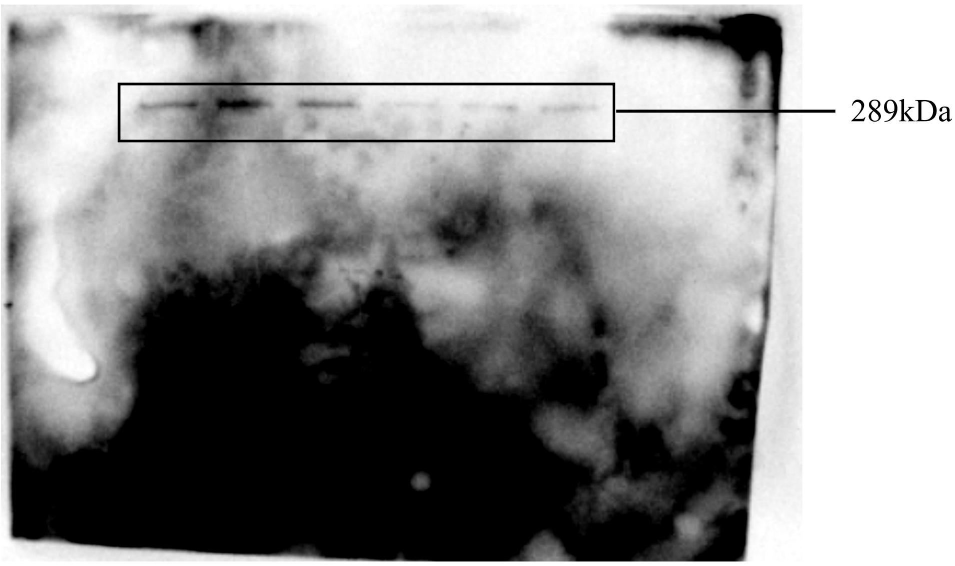

The 2nd time:

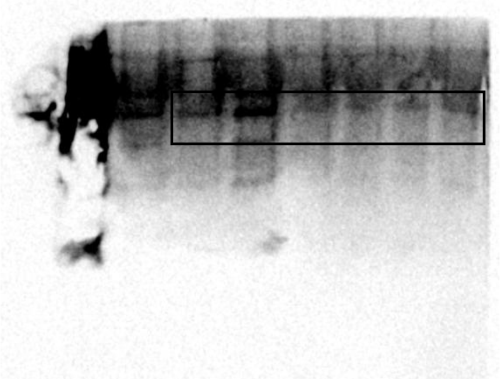

The 3 rd time:

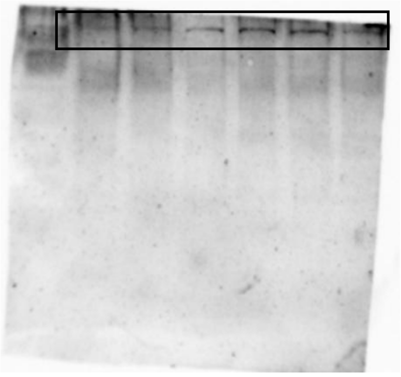

Figure 1(A)-15 The original images of the western blotting of the expression level of mTOR in BV2 cells of each group for three independent repeats. (From left to right in the black text box are Control group, Model group, Ajugol-H group, Ajugol-Lgroup, Isoacteoside-H group, Isoacteoside-L group, respectively. The first western blotting

band is presented as a representative image in the manuscript.)

The 1st time:

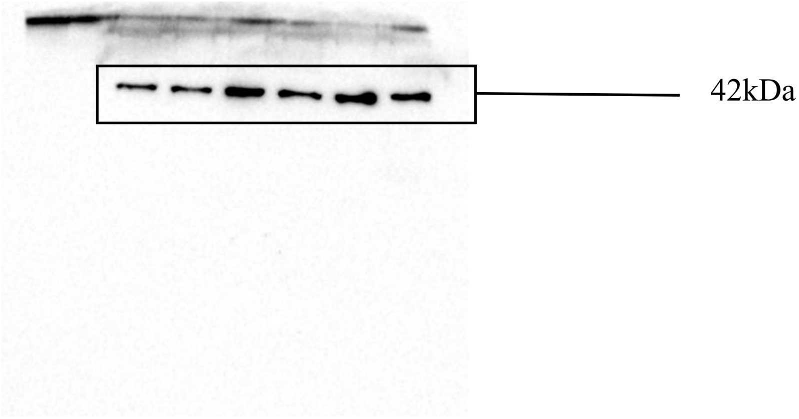

The 2nd time:

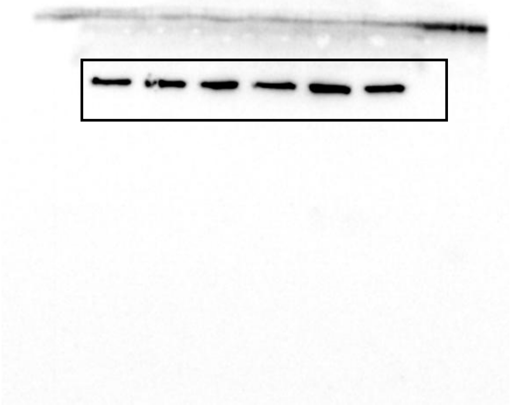

The 3 rd time:

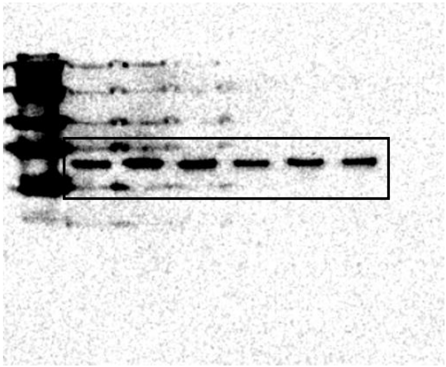

Figure 1(A)-16 The original images of the western blotting of the expression level of

β-actin in BV2 cells of each group for three independent repeats. (From left to right in the black text box are Control group, Model group, Ajugol-H group, Ajugol-Lgroup, Isoacteoside-H group, Isoacteoside-L group, respectively. The first western blotting band is presented as a representative image in the manuscript.)
